# Advances in Mechanochemical Methods for One‐Pot Multistep Organic Synthesis

**DOI:** 10.1002/chem.202500798

**Published:** 2025-05-24

**Authors:** Nina Biedermann, Michael Schnürch

**Affiliations:** ^1^ Institute of Applied Synthetic Chemistry TU Wien Getreidemarkt 9/163 Vienna 1060 Austria

**Keywords:** ball milling, liquid‐assisted grinding, mechanochemistry, solvent free, sustainable synthesis

## Abstract

Mechanochemical synthesis has emerged as a powerful and more sustainable alternative to conventional solution‐based methods, offering advantages such as no or only minimal solvent use, reduced reaction times, and simplified operational conditions. The integration of multiple steps into a single reaction vessel further enhances these benefits by eliminating workup and purification steps, reducing waste, and often improving overall efficiency. This review highlights recent advancements in mechanochemical one‐pot multistep reactions in organic synthesis, focusing on protocols with sequential one‐pot operation. Diverse transformations are covered, including heterocycle formation, functional group interconversions, and the synthesis of active pharmaceutical ingredients, while discussing both the operational and environmental advantages of these methodologies, along with their remaining challenges. Overall, mechanochemical one‐pot synthesis has the potential to streamline transformations and therefore contribute to more sustainable approaches in modern organic synthetic chemistry.

## Graphical abstract

This review highlights recent advances in mechanochemical methods for multistep organic synthesis, focusing on sequential one‐pot approaches performed by grinding or ball milling. The presented transformations are discussed in terms of efficiency, applicability, and sustainability, assessing both the challenges and potential of solvent‐free one‐pot approaches to contribute to greener strategies in organic synthesis.

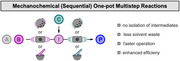

## 1 Introduction

Chemical synthesis has continually evolved to meet increasing demands for efficiency, sustainability, and cost‐effectiveness.^[^ ^1^ ^]^ Traditional synthesis methods, which are often solution‐based, have numerous drawbacks including the high consumption of organic solvents, the use of reactive and toxic reagents, long reaction times, and elaborate, costly purification processes.

In recent years, mechanochemistry, along with other alternative energy input techniques such as microwave‐ or ultrasound‐assisted chemistry,^[^ ^2^ ^]^ has emerged as a promising alternative to traditional methods. Mechanochemistry utilizes mechanical impact and friction, generated by grinding, ball milling, or extrusion, to facilitate reactions in the solid state without or with only minute amounts of solvent. This alternative way of energy input has opened new reaction pathways in molecular synthesis and, in general often results in rapid reaction kinetics.^[^ ^3^, ^4^, ^5^, ^6^ ^]^ Still, the primary fascination for mechanochemical synthesis lies in the reduced use of solvents and the associated waste. Consequently, mechanochemistry offers a more environmentally sustainable and greener form of synthesis, making it highly attractive to both academia and, especially, industry, where less solvent‐intense isolation procedures such as distillation and recrystallization are predominant in contrast to chromatographic methods on a small scale.^[^ ^7^, ^8^, ^9^, ^10^, ^11^, ^12^ ^]^

Another important advancement in modern synthetic chemistry is the development of one‐pot synthesis. These types of reactions, where multiple steps are performed in a single reaction vessel, offer considerable advantages over classical multistep syntheses. The primary benefit of this approach is the reduction in the number of consecutive reaction steps, which minimizes or eliminates the need for waste intensive, laborious, and time‐consuming intermediate purifications. This significantly shortens the overall synthesis process, reduces the amount of waste generated, and in many cases results in increased overall yields, contributing toward greener and more sustainable chemistry.^[^ ^13^, ^14^, ^15^, ^16^ ^]^

**Figure 1:**
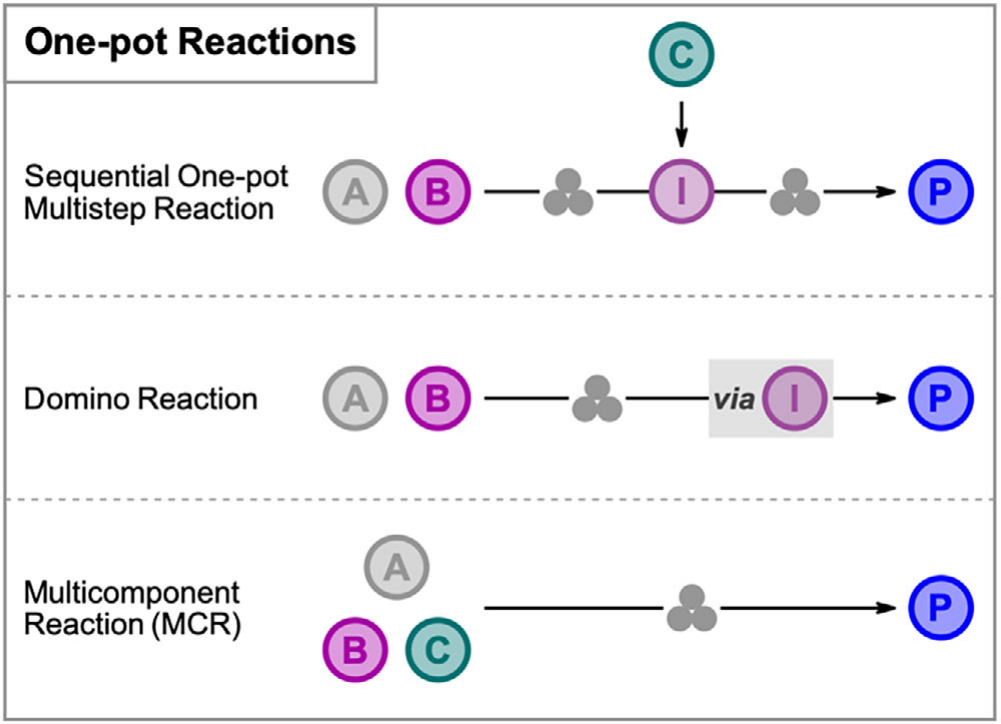
Conceptional overview of three types of mechanochemical one‐pot reactions.

The combination of mechanochemistry and one‐pot synthesis holds the potential to make an even larger contribution in this direction since this synergistic approach enables the more environmentally friendly and resource‐efficient production of chemical compounds. Different types of one‐pot reactions are distinguished according to their operation procedures: (1) Sequential one‐pot multistep reactions, where reactions are carried out in a stepwise fashion with direct transformation of intermediate products by sequential addition of reactants and reagents. (2) Domino reactions, in literature also often described as cascade or tandem reactions, where two or more transformations take place under the same conditions, without the addition of reactants, reagents, and catalysts, and subsequent reactions result as a consequence of the functionality formed in the previous step. (3) Multicomponent reactions, where three or more reactants react in a single‐operation reaction as, for example, the Ugi and Groebke‐Blackburn‐Bienayme reactions (Figure 1).^[^ ^16^ ^]^

**Figure 2:**
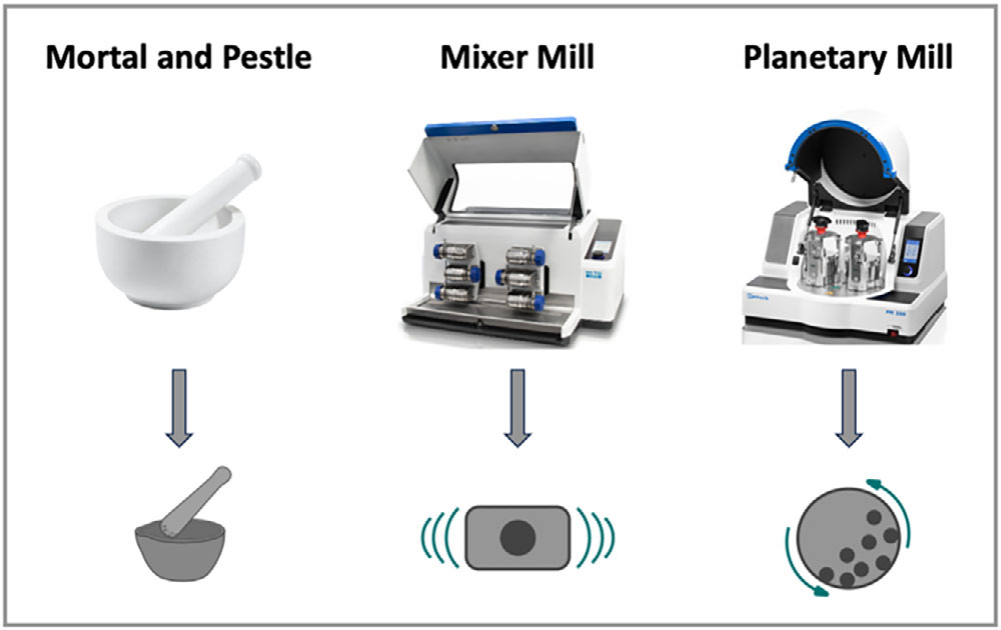
Most commonly used devices on a laboratory scale to conduct mechanochemical reactions and the symbols presenting each device throughout this review. Images reproduced with permission from the company Retsch (mixer mill and planetary mill) and stock.adobe.com (mortar and pestle), licensed for use.

On a laboratory scale, the most common instruments include simple mortar and pestle setups for manual grinding, a historical technique used since the Stone Age,^[^ ^17^ ^]^ and mixer mills and planetary ball mills for automated milling (Figure 2). The latter ones differ in their motion types, energy input, and also scale. Mixer mills operate through back‐and‐forth linear shaking at frequencies typically between 15 and 35 Hz, generating mechanical impact by collision of the reactants and balls with the jar wall, and are suitable for milligram‐ to gram‐scale synthesis. In contrast, planetary mills generate centrifugal forces through the rotation of jars, often providing higher impact energies due to the combined impact and shearing forces, and are better suited for larger‐scale reactions from gram to kilogram.^[^ ^18^, ^19^, ^20^, ^21^ ^]^ The choice of milling jar and ball materials – commonly stainless steel (SS), zirconia (ZrO_2_), PTFE (Teflon), or agate – can directly influence the reaction outcome through differences in hardness, density, and the potential for abrasion or contamination. In general, the selection of milling jars (material and size) and balls (number, material, and size) must be tailored to the desired mechanical energy transfer, compatibility with reagents, and to minimize potential contamination. Jar volumes typically range from 7 to 250 mL, and ball sizes from 1 mm to centimeters. The number, size, and material of the milling balls directly affect the energy input and mixing efficiency.^[^ ^22^, ^23^ ^]^

In addition to these instrumental parameters, the use of additives such as grinding auxiliaries (GAs) or liquids in liquid‐assisted grinding (LAG) can significantly impact reaction performance regarding kinetics, efficiency, and also selectivity. GA, which are inert solids such as salts or silica, are added to improve mixing or prevent agglomeration. LAG involves the addition of small amounts of liquid (commonly less than 1 µL per mg of solid reactants) to enhance reactivity or selectivity.^[^ ^24^, ^25^ ^]^

Overall, these operational details, including milling instrumentation, jar and ball selection, milling frequency, time, and the presence of additives drastically affect reaction rates, yields, selectivity, and product profiles.

This review will highlight recent advances in the exciting field of mechanochemical one‐pot multistep reactions in organic synthesis, including literature published from 2018 to the present. Our primary focus is on sequential one‐pot multistep reactions performed by grinding or ball milling (mixer or planetary mills), where intermediates are thermodynamically stable and could, in principle, be isolated or may offer potential for future mechanochemical transformations. Additionally, a few notable examples of one‐pot transformations with in situ formation of intermediates (domino‐type reactions) will also be discussed. Various transformations including the formation of (bis‐)heterocycles, the introduction of key functional groups, such as fluorine‐containing groups, and the formation of amide bonds (besides peptide synthesis) are presented. Furthermore, we will highlight the synthesis of important active pharmaceutical ingredients (APIs) including mechanochemical one‐pot reaction steps and provide an insight into the challenges and potential of combining various reactions in a one‐pot mechanochemical setting. Mechanochemical coupling reactions for the formation of amide bonds and peptide coupling which are performed via one‐pot condensation of an amine with a carboxylic acid via an active ester using coupling reagents are excluded from this review since similar existing solution‐based approaches also mainly proceed in a one‐pot fashion without isolation of the activated ester intermediate.^[^ ^26^, ^27^ ^]^ With this review, our goal is not only to present interesting mechanochemical protocols (MC) in a concise way but also to discuss them in comparison to similar solution‐based protocols (SC) from a critical point of view and evaluate the mechanochemical methods in terms of their contribution to the development of greener, more sustainable reactions. For the latter, green chemistry metrics will be discussed if the corresponding data is provided in the original publication, including atom economy/efficiency (AE), environmental factor (E‐factor), process mass intensity (PMI), reaction mass efficiency (RME), molar efficiency (Mol.E), carbon efficiency (CE), and EcoScale.^[^ ^28^ ^]^ In this context, it has to be mentioned that the provided calculations deviate from the established formulas in some cases but were at least applied identical for the mechanochemical and the solution phase process. Recalculation of these values with the correct formula was beyond the scope of this review. However, we want to point out that green metrics provided in literature require critical evaluation, not only in the context of this review, but generally.

For a few transformations we will also provide an insight into the proposed underlying mechanism. Our focus here lies on reactions with a mechanochemical mechanism, transformations that represent a very different approach compared to similar solution‐based pathways, or feature interesting mechanistic pathways. However, mechanisms for well‐known transformations that were translated into mechanochemical processes from classical solution chemistry without probing whether there is a change in mechanism or where mechanisms were proposed without sufficient experimental data proving a tentative mechanistic pathway are excluded. This is to avoid reproduction of such mechanisms and them becoming “common knowledge” even though sufficient evidence is lacking. Additionally, most reports do not discuss the reaction mechanism.

Noteworthy, a mini‐review article on mechanochemical and microwave multistep organic reactions has been recently published by Margetić,^[^ ^29^ ^]^ which also included mechanochemical one‐pot multistep reactions. However, only a limited number of synthetic protocols from recent years is discussed. For completeness, these transformations are included in this review, along with various other one‐pot protocols not covered in the aforementioned article.

## 2 Synthesis of (Bis‐)Heterocycles

### 2.1 Indole and Quinoline Scaffolds

Indole and its hydrogenated variant, indoline, are privileged scaffolds incorporated in diverse medicinally relevant compounds.^[^ ^30^ ^]^ Many indole‐ or indoline‐based anti‐cancer drugs have been developed and successfully implemented in the treatment of cancer as, for example, alectinib or vinblastine which are both so‐called cancer growth blockers.^[^ ^31^, ^32^ ^]^ Therefore, more sustainable synthetic pathways are of high interest. Next to indole and indolines, quinoline and hydrogenated variants are common scaffolds in many natural and synthetic compounds of medicinal significance but are also widely applied in materials science.^[^ ^33^, ^34^ ^]^

**Scheme 1:**
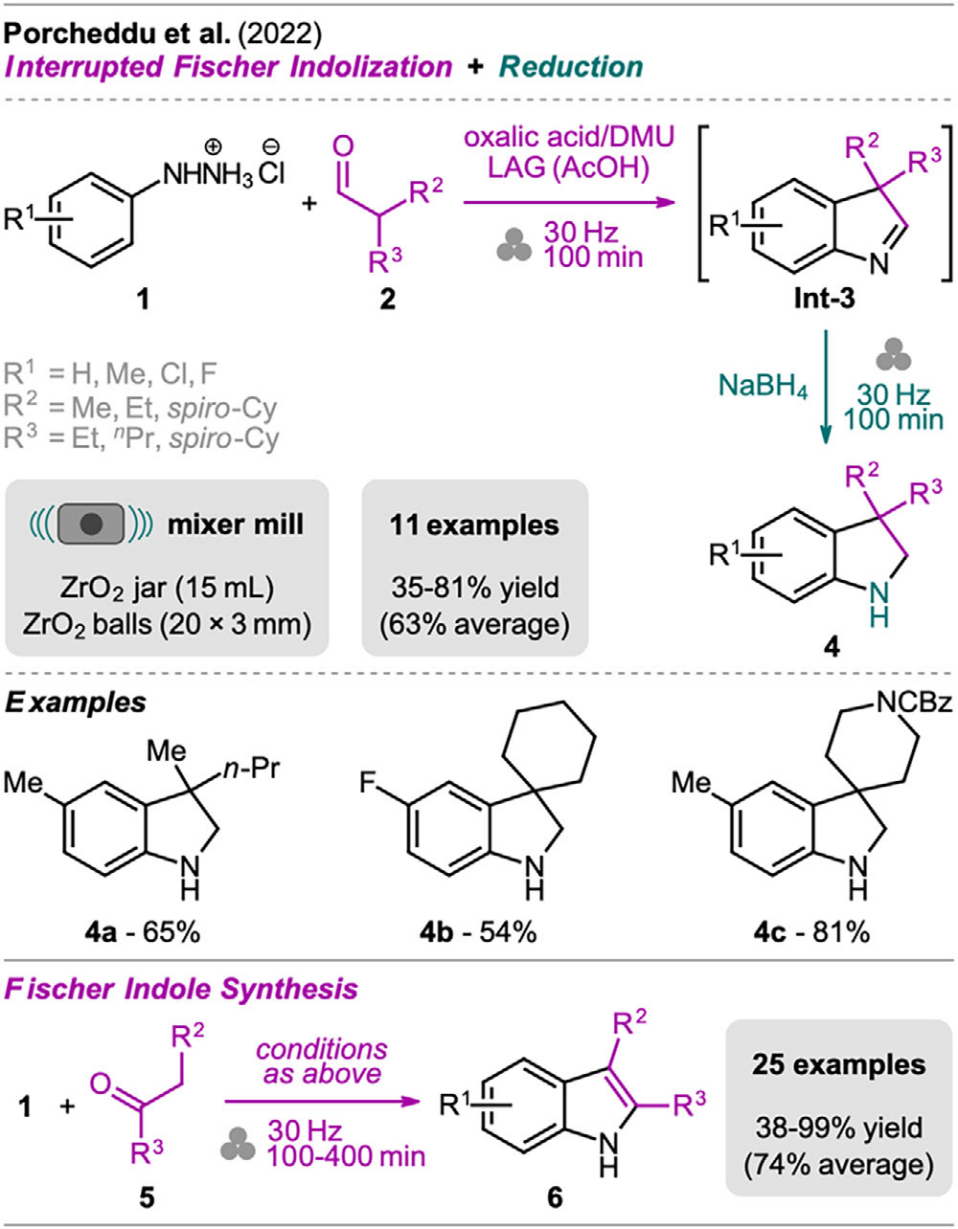
Synthesis of indolines via an interrupted Fischer indolization and reduction sequence (top) and synthesis of indoles under the same conditions used for the interrupted Fischer indolization (bottom).

A simple protocol for the synthesis of 3,3‐disubstituted indolines has been reported by Porcheddu et al. (Scheme 1, top), combining the Fischer indole synthesis and the reduction step in a one‐pot two‐step sequence.^[^ ^35^ ^]^ The synthesis was started with an interrupted Fischer indolization of aryl hydrazine hydrochloride **1** and 2,2‐disubstituted aldehyde **2** with an excess of oxalic acid and dimethylurea (DMU), both solids, under LAG conditions using acetic acid (AcOH). Subsequently, the indolenine intermediate **Int‐3** was treated with sodium borohydride in a second ball milling step to obtain the indoline compound **4**.

Exploring the applicability of this protocol, methyl‐ and halo‐substituted aryls **1** together with cyclohexanecarbaldehyde, Cbz‐protected 4‐formylpiperidine, or 2‐methylpentanal, for example, gave the indolines **4** in moderate to good yields. Notably, 1,2‐alkyl migration toward the thermodynamically more stable indole ring was not observed under the mechanochemical conditions, which is often triggered by classical solution‐based protocols carried out in acetic acid.^[^ ^36^, ^37^ ^]^

The mechanochemical protocol has also been evaluated in terms of greenness by calculation of the E‐factor (214) and Eco‐scale, (52.3) and comparison to a one‐pot solution protocol (E‐factor: 278, Eco‐scale: 45.0).^[^ ^38^ ^]^ Both values show an improvement for the mechanochemical approach, but the E‐factor of the mechanochemical process is still high due to the necessity of an aq. workup and column chromatography (CC) (E‐factor without workup and CC: 5.0 (MC) vs. 58.9 (SC)). However, in the solution‐based approach, elevated temperatures and the use of 1,2‐dichloroethane (DCE), a legally restricted solvent, are reported, further highlighting the superiority of the mechanochemical approach.

In addition to the synthesis of indolines, the authors presented the mechanochemical Fischer synthesis of indoles **6** from aryl hydrazine **1** and various ketones **5** under the same conditions used for the interrupted Fischer indolization toward indolines (Scheme 1, bottom).^[^ ^35^ ^]^

**Scheme 2:**
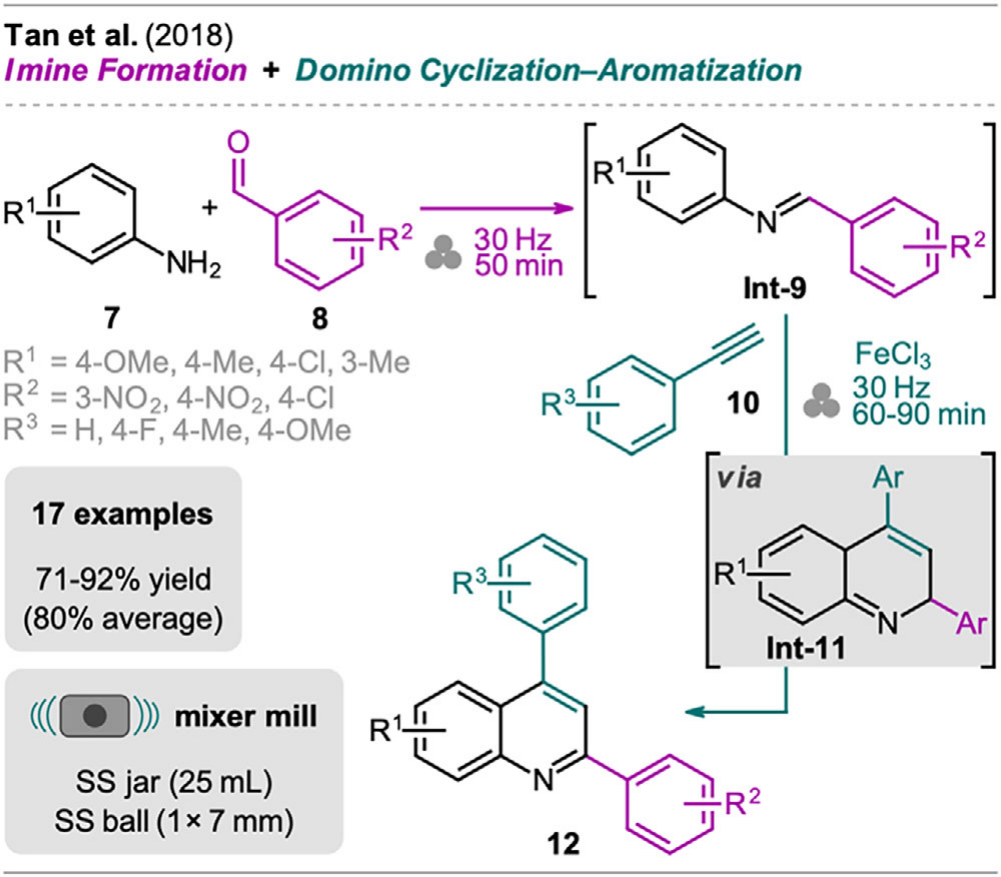
Synthesis of quinolines via an imine formation and domino cyclization‐aromatization sequence.

A sequential one‐pot synthesis of 2,4‐diarylsubstituted quinolines starting from anilines, benzaldehydes, and phenylacetylenes was developed by Tan et al. (Scheme 2).^[^ ^39^ ^]^ As the first step, milling of aniline **7** with benzaldehyde **8** was performed in a mixer mill without additives to form the imine intermediate **Int‐9**. Subsequently, phenylacetylene (**10**) was added together with an excess of a strong Lewis acid (FeCl_3_) to achieve the domino cyclization–aromatization toward the substituted quinoline **12** via **Int‐11**. An excess of FeCl_3_, which serves as a one‐electron oxidant in its hydrated form, was found to be crucial since catalytic amounts gave almost complete conversion of **Int‐9** but mainly to the dihydroquinoline intermediate **Int‐11** due to incomplete oxidation. Other strong Lewis acids (e.g., ZnCl_2_ or AlCl_3_) did not succeed in the cyclization–aromatization domino reaction, resulting in **Int‐11** as the main product (not isolated).

In the course of exploring the substrate scope, reactions of anilines with strong EDGs and acetylenes with strong EWGs proceeded more smoothly, resulting in higher yields. Highlighting the practicability of this protocol, extraction and column chromatography were not necessary. Pure quinolines **12** were obtained by washing with water and recrystallization from EtOH/H_2_O mixtures.

Additionally, the reaction also proceeded when performed in a one‐pot multicomponent fashion, without preformation of the imine intermediate **Int‐9**, achieving slightly higher yields in the range from 74 to 95% (83% average) compared to the sequential two‐step protocol.

The synthesis of similar quinolines has, for example, been reported under montmorillonite, a mineral of the group of phyllosilicates,^[^ ^40^ ^]^ or potassium dodecatungstocobaltate trihydrate catalysis^[^ ^41^ ^]^ via microwave‐assisted (MW) multicomponent reaction under neat conditions with reaction times of max. 15 minutes. Comparing these two methods to the MC protocol, the MW‐assisted approaches clearly stand out in terms of reaction time. However, when it comes to scalability and energy efficiency, the MC approach can still be seen as a valuable synthetic method, probably interesting for industrial application due to the simple operational set‐up.

**Scheme 3:**
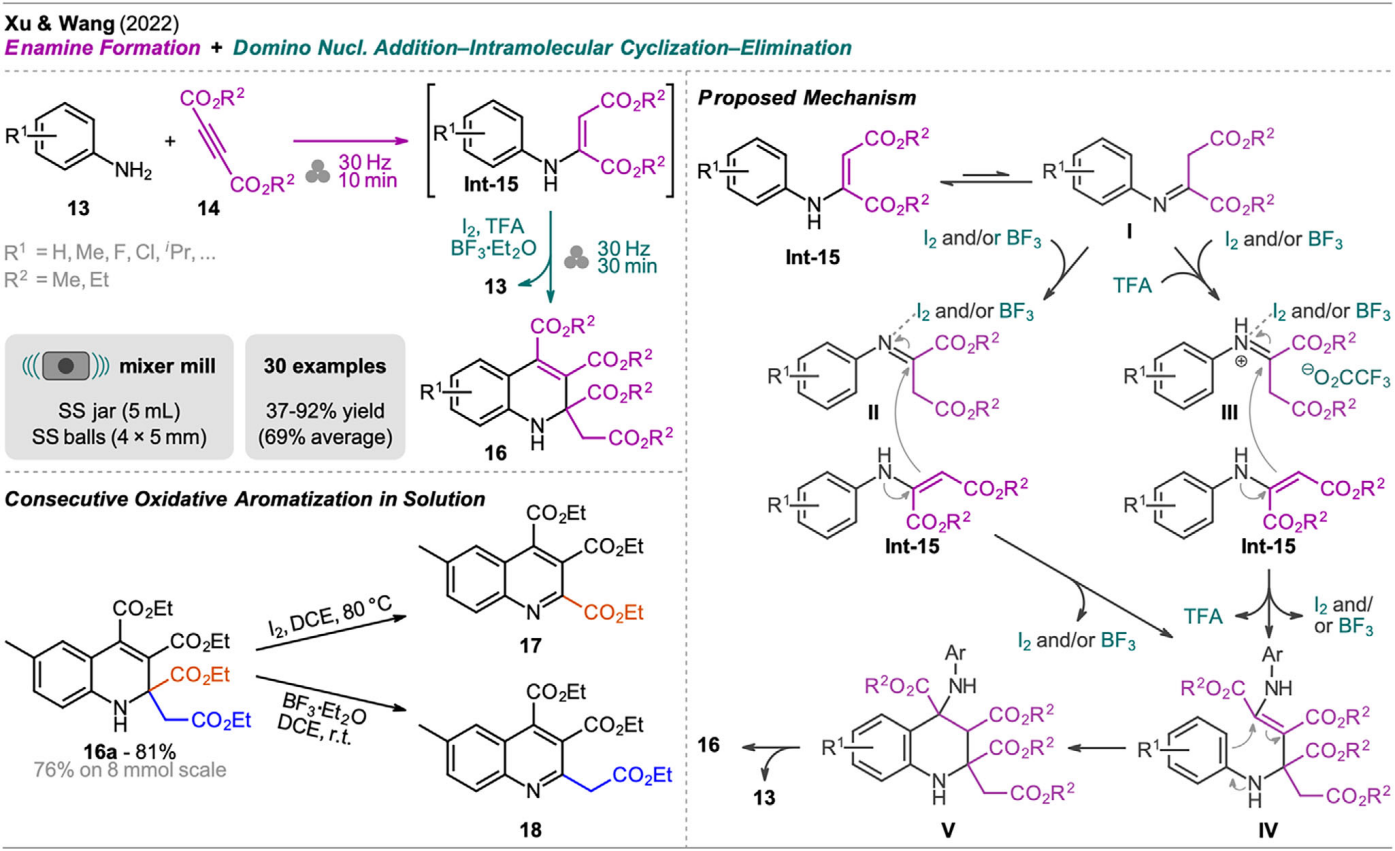
Synthesis of 1,2‐dihydroquinolines via an enamine formation and domino nucleophilic addition‐intramolecular cyclization followed by elimination.

Xu and Wang reported a one‐pot synthesis of polysubstituted 1,2‐dihydroquinolines bearing multiple ester substituents from anilines and dialkyl actylenedicarboxylates (Scheme 3, top left).^[^ ^42^ ^]^ This protocol features the preformation of an enamine intermediate **Int‐15** from anilines **13** and diesters **14**, and sequential addition of iodine, BF_3_·OEt_2_, and trifluoroacetic acid (TFA) afforded product **16** (Scheme 3). When only iodine or BF_3_·OEt_2_ was added, formation of product **16** was observed but with significantly lower efficiency. In contrast to the protocol by Tan et al., milling of all reactants and reagents at once afforded only trace amounts of the desired product **16** and significant amounts of enamine **Int‐15** and byproducts.

Based on control experiments, the authors identified the enamine **Int‐15** as the key intermediate in the transformation and proposed a plausible mechanism (Scheme 3, right). The β‐enamino ester **Int‐15**, formed by nucleophilic addition of the aniline **13** to the acetylene diester **14**, tautomerizes to the β‐imino ester **I**. With the assistance of I_2_ and/or BF_3_ or, additionally, TFA, intermediate I**I** or **III**, respectively, undergoes nucleophilic attack by another molecule of **Int‐15** affording intermediate **IV** after tautomerization. Subsequent intramolecular cyclization provides intermediate **V** which finally eliminates an aniline molecule **13,** resulting in the formation of the C ═ C bond and product **16**.

The method was explored with various anilines with alkyl‐ and halo‐substituents including a scale‐up experiment (20‐fold, 8 mmol) which did not show a significant reduction in yield. Interestingly, subsequent aromatization in solution with either iodine or BF_3_·OEt_2_ led to two different quinoline derivatives via cleavage of one of the functional groups in position C‐2, depending on the oxidant used (Scheme 3, bottom left).

**Scheme 4:**
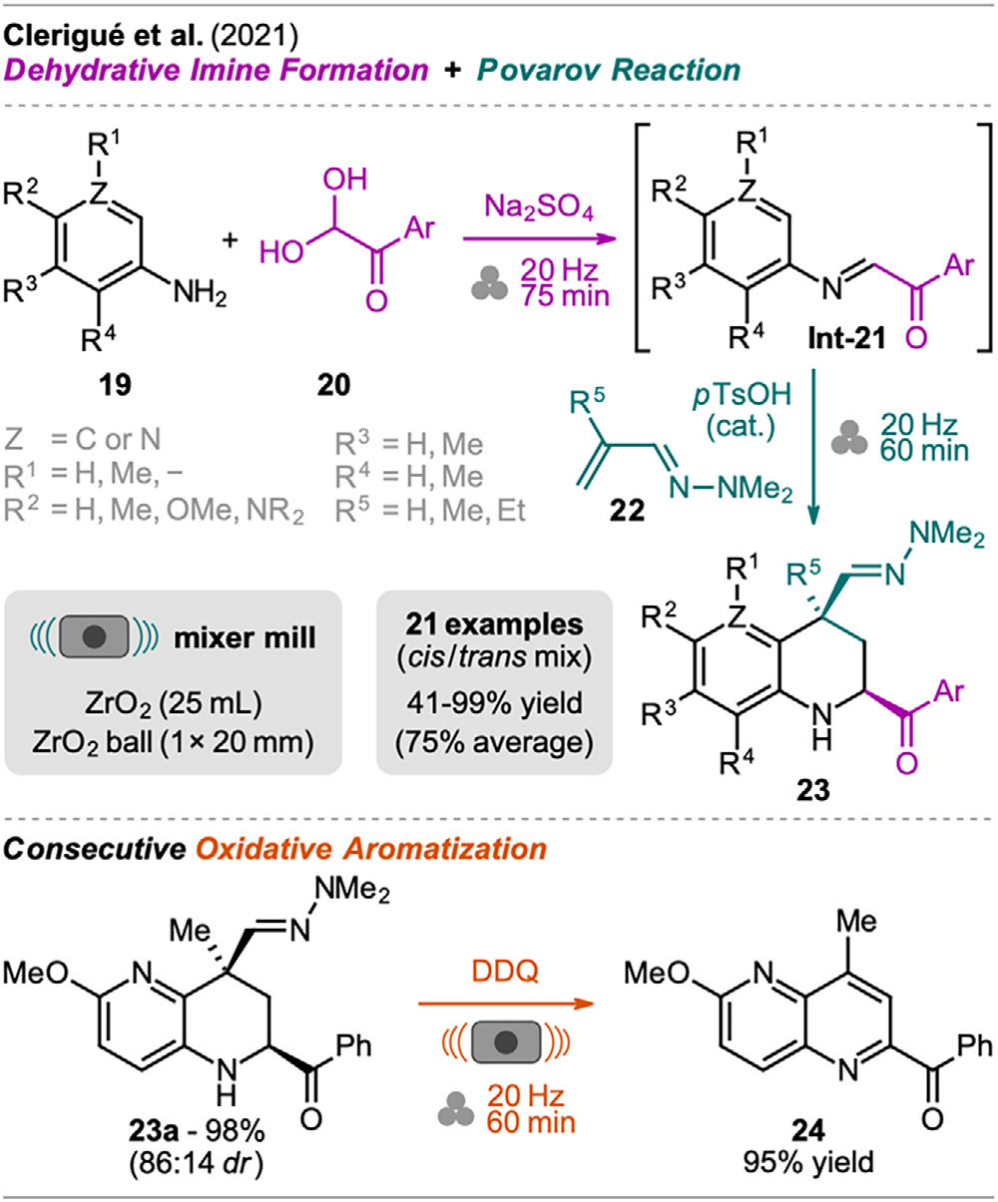
Synthesis of substituted 1,2,3,4‐tetrahydroquinolines and 1,2,3,4‐tetrahydro‐1,5‐naphthyridines via a dehydrative imine formation and Povarov reaction sequence.

1,2,3,4‐Tetrahydroquinolines with functionalization at C‐2 and C‐4 were prepared mechanochemically by Clerigué et al. (Scheme 4).^[^ ^43^ ^]^ The synthesis was carried out starting from aniline **19** (Z = C) and glyoxal derivative **20** which were milled with anhydrous Na_2_SO_4_ as a GA (acts as an absorbent for H_2_O formed from **20**) to give the α‐ketoimine **Int‐21**. Subsequently, hydrazone **22** and a catalytic amount of *p*‐toluenesulfonic acid (*p*TsOH) were added to the jar, and a second milling step was performed giving the tetrahydroquinoline **23** via Povarov reaction. The reaction resulted in a mixture of both possible diastereomers but with the *cis*‐isomer as the major one (>70:30 *dr* for most examples). The reaction's yields displayed in Scheme 4 are the isolated yields of the *cis*/*trans* mixtures. Other tested acid catalysts (e.g., FeCl_3_, Sc(OTf)_3_, InCl_3_) did not improve the *dr* while resulting in lower overall efficiency. However, pure ***cis*‐23** and ***trans‐23*** could be obtained upon a second column chromatography.

The mechanochemical protocol was compared to a solution‐based protocol by Bianchini et al., performed in two consecutive steps, in regards to efficiency and selectivity.^[^ ^44^ ^]^ In general, the time for the imine formation was extended from 30 minutes (SC) to 75 minutes (MC), but for the Povarov reaction step only 1 hour of reaction time was necessary for all substrates, which required up to 5 hours in the solution‐based protocol. Furthermore, in solution a change of solvent was required for the two steps, not allowing one‐pot operation. However, the solution‐based protocol showed higher *cis*‐selectivity for the same substrates. In regard to this trend, it is unclear if the solvent‐free method is generally superior to the classical method. Calculation of green metrics would help in the evaluation of advantages and disadvantages of both protocols but was not included in the original publication.

In addition to quinolines, two examples of tetrahydro‐1,5‐naphthyridines **23** (Z = N), which are also of potential pharmaceutical interest,^[^ ^45^, ^46^ ^]^ were successfully synthesized with excellent yields of 98% for **23a** and 85% (not shown). Compound **23a** was furthermore treated with 2,3‐dichloro‐5,6‐dicyano‐1,4‐benzoquinone (DDQ) in the mixer mill, forming 2‐acyl‐1,5‐naphtyridine **24** in high yield. The outcome of this reaction was surprising since earlier work by the same group showed that tetrahydroquinolines with similar substituents underwent C‐4 to C‐3 rearrangement of the hydrazone substituent when treated with DDQ in solution.^[^ ^47^ ^]^

### 2.2 Pyrrole Scaffolds

Another important class of heterocycles are pyrroles and pyrrolidines, motifs present in numerous bioactive compounds and pharmaceuticals, but also agrochemicals or functional materials.^[^ ^48^, ^49^, ^50^, ^51^ ^]^

**Scheme 5:**
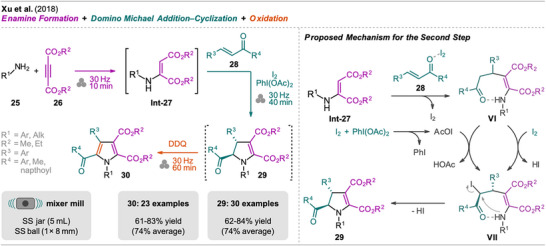
Synthesis of pyrroles via an enamine formation, domino Michael addition‐cyclization and oxidation sequence. *trans*‐2,3‐Dihydropyrroles could be obtained without the oxidation step.

A simple mechanochemical one‐pot synthesis of 2,3‐dihydropyrroles and pyrroles has been developed by Xu et al. (Scheme 5).^[^ ^52^ ^]^ The reaction sequence started with the formation of enamine **Int‐27** by milling of amine **25** with acetylenedicarboxylate **26** without additives.  α,β‐Unsaturated ketone **28** was then added together with iodine and PhI(OAc)_2_ and a second milling step was carried out which led to the domino Michael addition‐cyclization of **Int‐27** toward the 2,3‐dihydropyrrole **29** within a total reaction time of 50 minutes. The need for PhI(OAc)_2_ to achieve high conversion was an accidental finding and the use of only I_2_ resulted in lower conversion. Mechanistic considerations suggest the formation of AcOI which then, next to I_2_, reacts with the Michael addition product **VI** affording an iodine intermediate **VII**. This then readily undergoes an intramolecular S_N_2‐type nucleophilic substitution (cyclization) (Scheme 5, right). In the evaluation of different solvents for LAG, dichloroethane (DCE) was found to shorten the reaction time by 10 minutes. Nevertheless, DCE was not employed in the final reaction conditions to make the experimental process simpler and avoid the use of a chlorinated solvent. When the reaction was performed by mixing all reactants and reagents at once, the desired product was only formed in a poor yield of 18% together with unreacted enamine and byproducts. A scale‐up experiment (20‐fold, 4 mmol) of chalcone **28** (R^3^ = R^4^ = Ph) was performed and with a slightly longer reaction time (70 minutes in total) the corresponding dihydropyrrole was obtained in gram quantity and a good yield of 79% (80% on a 0.2 mmol scale). Next to various chalcone and aniline derivatives, aliphatic amines were also employed in the one‐pot reaction, affording the corresponding products in good to excellent yield.

The method was also extended by a third step, the oxidation of **29** toward the corresponding pyrroles **30** in one‐pot with DDQ. This one‐pot three‐step protocol was demonstrated for the synthesis of various polysubstituted pyrroles achieving very good yields of over 61%.

In solution, pyrrole scaffolds similar to **30** were synthesized by Yan et al. starting from β‐enamine ketones and acetylenedicarboxylates with CuI under an oxygen atmosphere in dimethylformamide (DMF) at 80 °C.^[^ ^53^ ^]^ Comparing these two methods, the advantages of the mechanochemical protocol can be highlighted: (1) the starting materials are readily available, whereas the solution‐based protocol required the preparation and isolation of the enamine prior to the Michael addition‐cyclization reaction, resulting in more solvent waste; (2) the mechanochemical reaction does not require the use of any solvent (except for the isolation and purification), whereas the solution approach uses DMF, a solvent harmful to health; and (3) no external heating is required when performing the reaction in the ball mill.

**Scheme 6:**
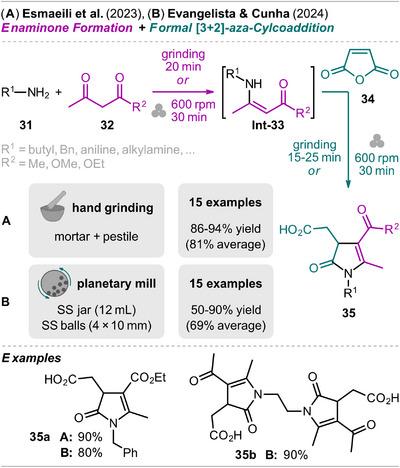
Synthesis of β,γ‐unsaturated butyrolactams via an enaminone formation and formal [3+2]‐aza‐cycloaddition sequence.

β,γ‐Unsaturated butyrolactams, representatives of 2‐pyrrolidinones, have been synthesized in a mechanochemical one‐pot two‐step process by Evangelista and Cunha,^[^ ^54^ ^]^ but also in an earlier report from Esmaeili et al.^[^ ^55^ ^]^ (Scheme 6). Both approaches perform stepwise the formation of enaminone intermediate **Int‐33** and subsequent formal [3+2]‐aza‐cycloaddition with maleic anhydride (**34**) to give pyrrolidinones **35**. Esmaeili et al. perform the reaction by hand grinding using a mortar and a pestle. Equimolar amounts of primary amine **31** and β‐ketoester **32** (R^2^  =  OAlk) were grinded for 20 minutes without any additive, then maleic anhydride (**34**) was added and grinding continued until TLC indicated complete conversion to **35** (15‐25 minutes).

The same route for the synthesis of β,γ‐unsaturated butyrolactams was taken by Evangelista and Cunha but the sequence was performed in a planetary mill. Furthermore, the authors also demonstrated the possible isolation of enaminones **Int‐33** via recrystallization or extraction (30 examples for **Int‐33** including primary and secondary amines **31**). Regarding the substrate scope of pyrrolidinones, both reports include aliphatic primary amines but also aniline was shown to efficiently react with acetylacetone and methyl or ethyl 3‐oxobutanoate. In the planetary mill approach, diamines were also used as starting materials and the corresponding bis‐enaminones and further bis‐lactams were obtained in good to excellent yield of up to 90% (e.g., compound **35b**).

Esmaeili et al. demonstrated isolation of the products by CC while Evangelista and Cunha showed that either recrystallization from ethyl acetate (EtOAc)/hexane mixtures (for solids) or extraction using EtOAc/H_2_O (for oils) is sufficient to obtain pyrrolidinones **35** with high purity and the advantage of less solvent use compared to CC.

**Scheme 7:**
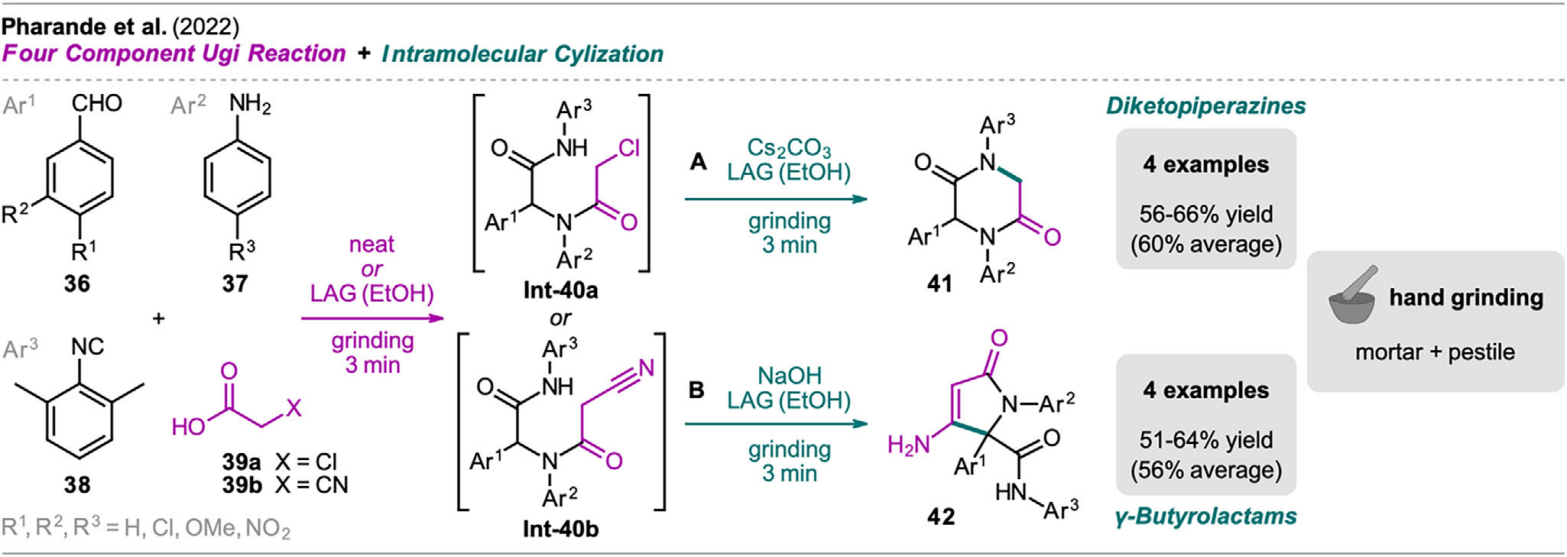
Synthesis of 2,5‐diketopiperazines (A) and α,β‐unsaturated γ‐butyrolactams (B) via an Ugi reaction and intramolecular cyclization sequence.

In 2022, Pharande et al. presented a one‐pot grinding approach for the synthesis of tri‐substituted 2,5‐diketopiperazines (DKPs) and α,β‐unsaturated γ‐butyrolactams (Scheme 7).^[^ ^56^ ^]^ For both scaffolds, a mechanochemical Ugi four‐component reaction (Ugi 4‐CR), employing equimolar amounts of an aromatic aldehyde **36**, aniline derivative **37**, aromatic isocyanide **38**, and either chloroacetic acid (**39a**, for DKPs) or cyanoacetic acid (**39b**, for lactams), was performed as the first step. The reaction was conducted under LAG conditions using EtOH or by neat grinding, depending on X in **39**. Remarkably, the dipeptide intermediates **Int‐40a** and **Int‐40b** were obtained in short reaction time of only three minutes. Furthermore, the authors demonstrated that the Ugi adducts could be isolated by recrystallization in good to excellent yields, and various combinations of aldehydes, amines, isocyanides, and carboxylic acid compounds (not only chloro‐ **39a** and cyanoacetic acid **39b**) were presented (15 examples of **40** with an average yield of 81%). Earlier work reports a mechanochemical Ugi 4‐CR in a ball mill under InCl_3_ catalysis and LAG conditions (MeOH).^[^ ^57^ ^]^ In comparison, the grinding approach presented by Pharande et al. offers several advantages, including significantly shorter reaction times (3 vs. 45 minutes), the elimination of a catalyst, and also simplified isolation and purification through recrystallization instead of CC.

Pharande et al. then extended their mechanochemical approach to multistep one‐pot transformations, aiming to synthesize privileged heterocyclic peptidomimetics directly from the Ugi‐adducts. For DKPs, cesium carbonate and further EtOH were added to **Int‐40a** and grinding continued, giving the cyclized tri‐substituted DKPs **41** via intramolecular S_N_2 reaction in moderate yields (Scheme 7A). In a small substrate scope, including 4 examples, chloro‐ or methoxy‐substituted anilines **37** and arylaldehydes **36** were demonstrated as possible starting materials with slightly diminished yields compared to the combination of benzaldehyde and aniline (66%). For the synthesis of lactams from **Int‐40b**, the addition of sodium hydroxide and EtOH as LAG additive facilitated the cyclization toward substituted α,β‐unsaturated γ‐butyrolactams **42** via an intramolecular nucleophilic attack on the nitrile (Scheme 7B). Benzaldehydes **36** with EWGs (4‐Cl, 4‐NO_2_) and aniline derivatives **36** (4‐Cl, 4‐OMe) were included in the small substrate library.

Similar synthetic routes for the synthesis of DKPs and α,β‐unsaturated γ‐butyrolactams in one pot in solution have been reported earlier by the same group. For DKPs, Gámez‐Montaño and coworkers demonstrated a one‐pot Ugi reaction‐cyclization sequence with ethynamine instead of aniline derivatives **37** in methanol.^[^ ^58^ ^]^ The cyclization step was assisted by ultrasonication, but with long total reaction times of over 24 hours (24 hours for the Ugi‐reaction, 15 minutes for the S_N_2‐cyclization). For the synthesis of butyrolactams, the reaction was performed in a sequential one‐pot fashion in methanol, requiring heating to 65 °C during the second step and a total reaction time of >1 hour.^[^ ^59^ ^]^ In comparison, the mechanochemical approaches significantly reduce the overall reaction time to just 6 minutes while also eliminating the need for significant amounts of solvent and external heating.

### 2.3 Other (Fused) Nitrogen‐Containing Five‐Membered Heterocyclic Scaffolds

*N*‐Heterocyclic compounds are in general of great interest in drug design due to their pharmacological and physiological properties and are present in a wider range of bioactive molecules.^[^ ^60^, ^61^, ^62^, ^63^ ^]^ Within this chapter, mechanochemical one‐pot reactions for the following scaffolds are presented, which are of diverse medicinal interest: 1,2,4‐triazolo[3,4‐a]‐phathalazines,^[^ ^64^ ^]^ benzimidazoles,^[^ ^65^, ^66^ ^]^ 1,3,4‐oxadiazoles,^[^ ^67^ ^]^ and pyrazoles.^[^ ^68^, ^69^ ^]^

**Scheme 8:**
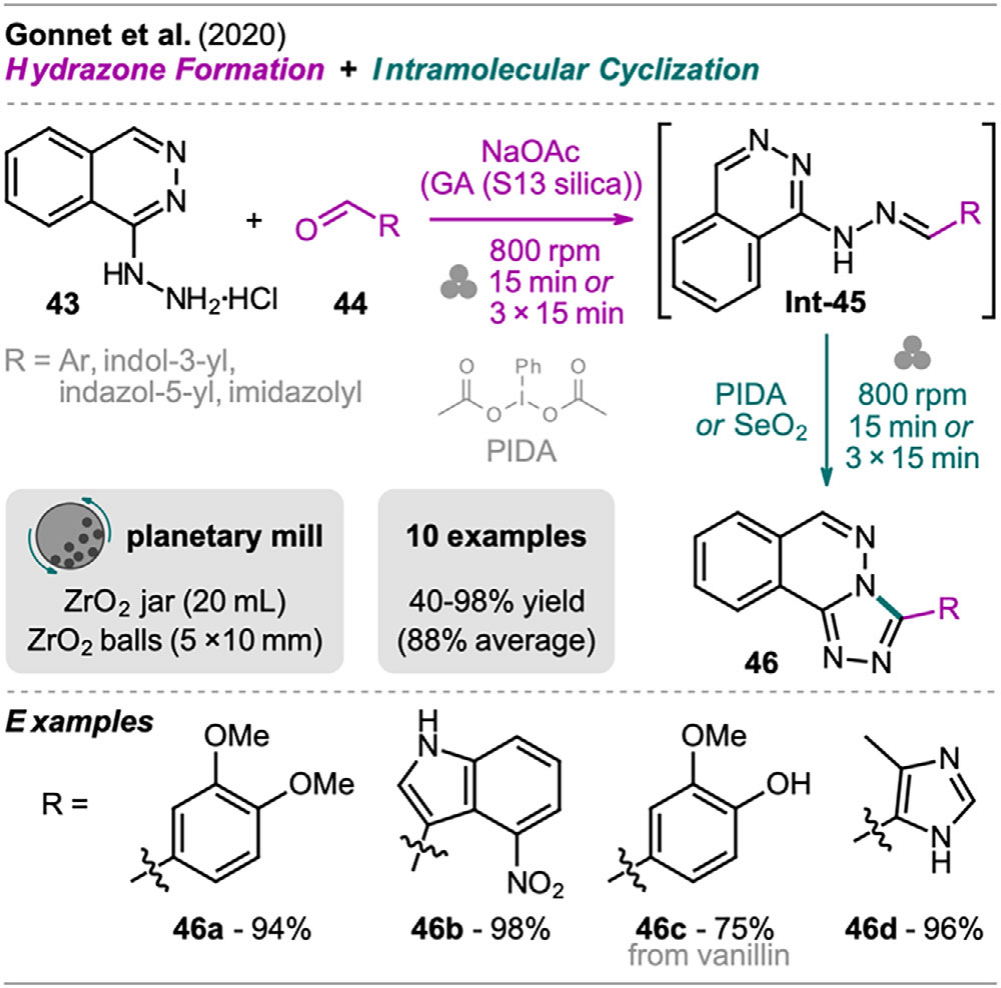
Synthesis of C‐3 substituted 1,2,4‐triazolo[3,4‐*a*]phthalazines via a hydrazone formation and intramolecular cyclization sequence.

A sequential one‐pot mechanochemical protocol for the synthesis of C‐3 substituted 1,2,4‐triazolo[3,4‐a]phthalazines has been investigated by Gonnet et al. in a planetary mill (Scheme 8).^[^ ^70^ ^]^ Starting from hydralazine **43** and aldehyde **44**, hydrazone **Int‐45** was formed in the first step with sodium acetate as an additive by three milling cycles of 15 minutes. The investigation of inert grinding additives revealed that the reaction time could be shortened to 15 minutes when pyrogenic silica (S13 silica) was used as GA (aldehyde **44** absorbed on S13 silica). The hydrazones **Int‐45** could be isolated by simple extraction but also directly converted into triazoles **46** by the addition of a cyclization agent and further milling. The optimized reaction conditions were investigated on isolated hydrazone **Int‐45** and the same observations as in the first step regarding the reaction time were made, allowing shorter reaction time in the presence of S13 silica. While iodobenzene diacetate (PIDA) was found to be the optimal reagent with nonphenolic hydrazones, selenium dioxide allowed the transformation of hydrazones bearing free hydroxyl groups (e.g., starting from vanillin) which gave poor yields using PIDA.

The general role of pyrogenic silica in the transformation was explained by, firstly, its ability to absorb water and acetic acid that is formed in the reaction. Secondly, its particle size (granulometry) was not sharply decreased in the performed milling process. The latter findings were obtained from a short study where the granulometry and specific surface area of different GAs (sand, silica gel, and pyrogenic silicas with differently modified surfaces) were analyzed before and after 15 minutes of milling in the used setup.

The methodology demonstrated applicability across various substrates using nonphenolic aryl and heteroaromatic aldehydes **44**, giving highly functionalized triazoles **46** in excellent yield from 93 to 98%. From vanillin and 4‐hydroxybenzaldheyde, triazoles were obtained in 75% (**46c**) and 40% yield (not shown), respectively, using SeO_2_.

The authors assessed the greenness of the developed protocol by comparing the E‐factor and PMI of the consecutive two‐step protocol with isolation of hydrazone **Int‐45** and a solution phase synthesis which was carried out in addition to the MC approach. The E‐factor of the mechanochemical transformation without GA was especially improved for the cyclization step, being reduced from 74 (SC) to 12 (MC), but without consideration of the work‐up and purification procedure. In regard to the provided data, the evaluation of the mechanochemical triazole synthesis in one‐pot including the final isolation and purification of product **46** would have provided a more realistic picture since solvents usually cannot be recycled in 100% quantity.

**Scheme 9:**
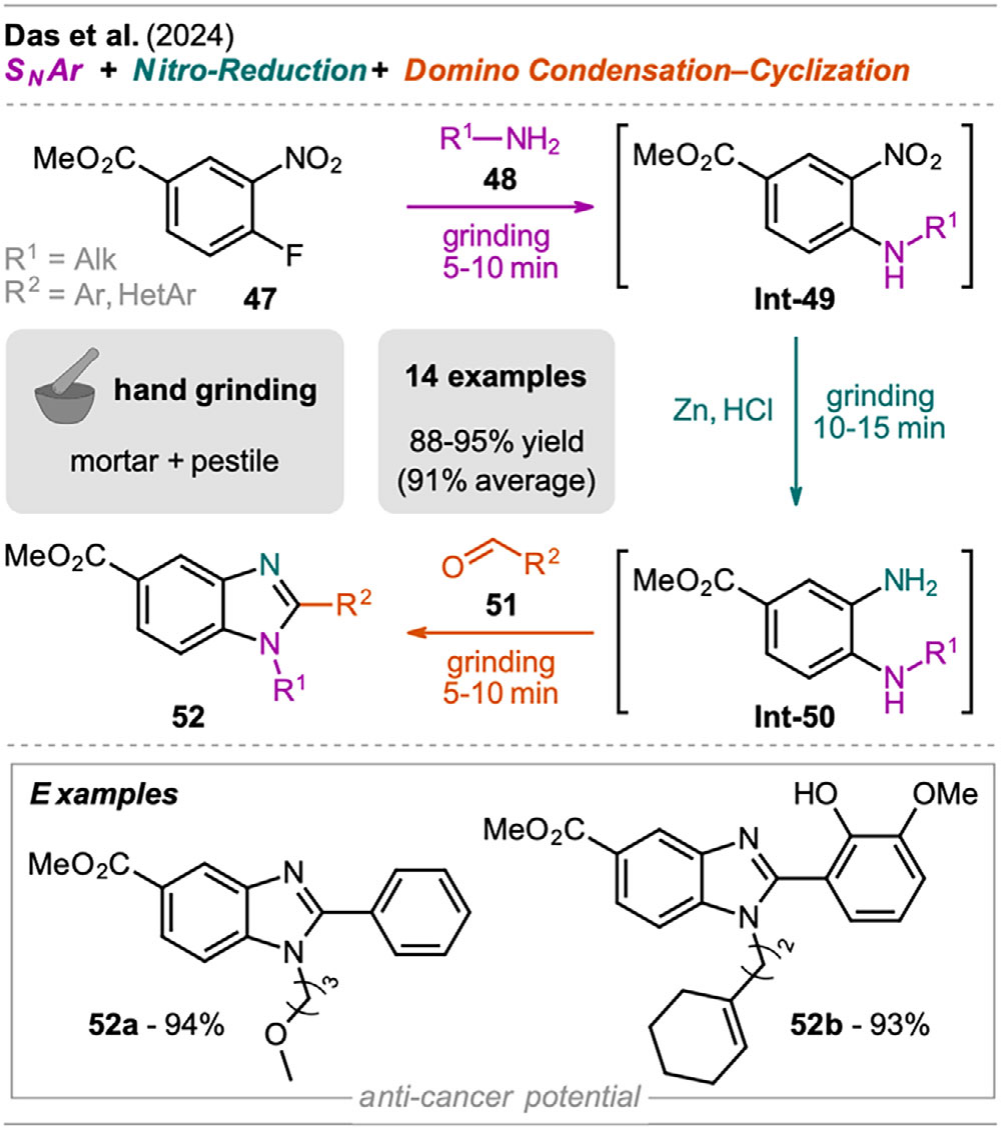
Synthesis of benzimidazoles with anti‐cancer potential via a three‐step one‐pot S_N_Ar, nitro‐reduction, and domino condensation‐cyclization sequence.

A sequential grinding approach for the one‐pot synthesis of benzimidazoles, including three steps was reported by Das et al. (Scheme 9).^[^ ^71^ ^]^ In a mortar, first the nucleophilic aromatic substitution of 4‐fluoro‐3‐nitrobenzoate (**47**) with primary amine **48** to form the secondary amine **Int‐49** was performed by simple grinding, then zinc dust together with hydrochloric acid was added, and grinding continued to obtain the diamine intermediate **Int‐50**. As the last step, aldehyde **51** was added, and further grinding gave substituted benzimidazoles **52** in an excellent yield of at least 88%. However, subsequent column chromatography was required, which might be replaceable by recrystallization on a larger scale. The method's applicability was demonstrated with various aryl aldehydes with EDGs and EWGs but also heteroaromatics, as, for example, furfuraldehyde. Additionally, the alkyl residue of the primary amine was varied.

Comparing the protocol to a similar solution‐based approach performed in three consecutive steps,^[^ ^72^ ^]^ the mechanochemical approach offers incredibly shorter reaction times (MC: approx. 30 minutes, SC: >40 hours), simplification of the reaction setup (SC required an inert atmosphere), and requires only one purification step instead of three. A more efficient approach in solution from Lin et al. shows that benzimidazoles can be synthesized directly from **Int‐49** via Pd‐catalyzed, iron pentacarbonyl‐mediated carbonylation of aryl iodides within 20 minutes under microwave radiation.^[^ ^73^ ^]^ However, yields are comparably lower, and prior synthesis and isolation of **Int‐49** is required.

In addition to the synthesis, the authors investigated the benzimidazole derivatives **52** as potential anti‐cancer drug candidates. Computational docking and dynamic simulation studies showed the ability of the substrates to interact with the proteins 3ERT, the ligand‐binding domain of human estrogen receptor α,^[^ ^74^ ^]^ and 5FGK, a complex between human kinase CDK8 and cyclin C,^[^ ^75^ ^]^ via various stabilizing interactions with generally higher docking scores for 3ERT. In experimental investigations on DNA binding and antioxidant activity, two derivatives with significant anti‐cancer potential were revealed as the most potent molecules (Scheme 9, compounds **52a** and **52b**).

**Scheme 10:**
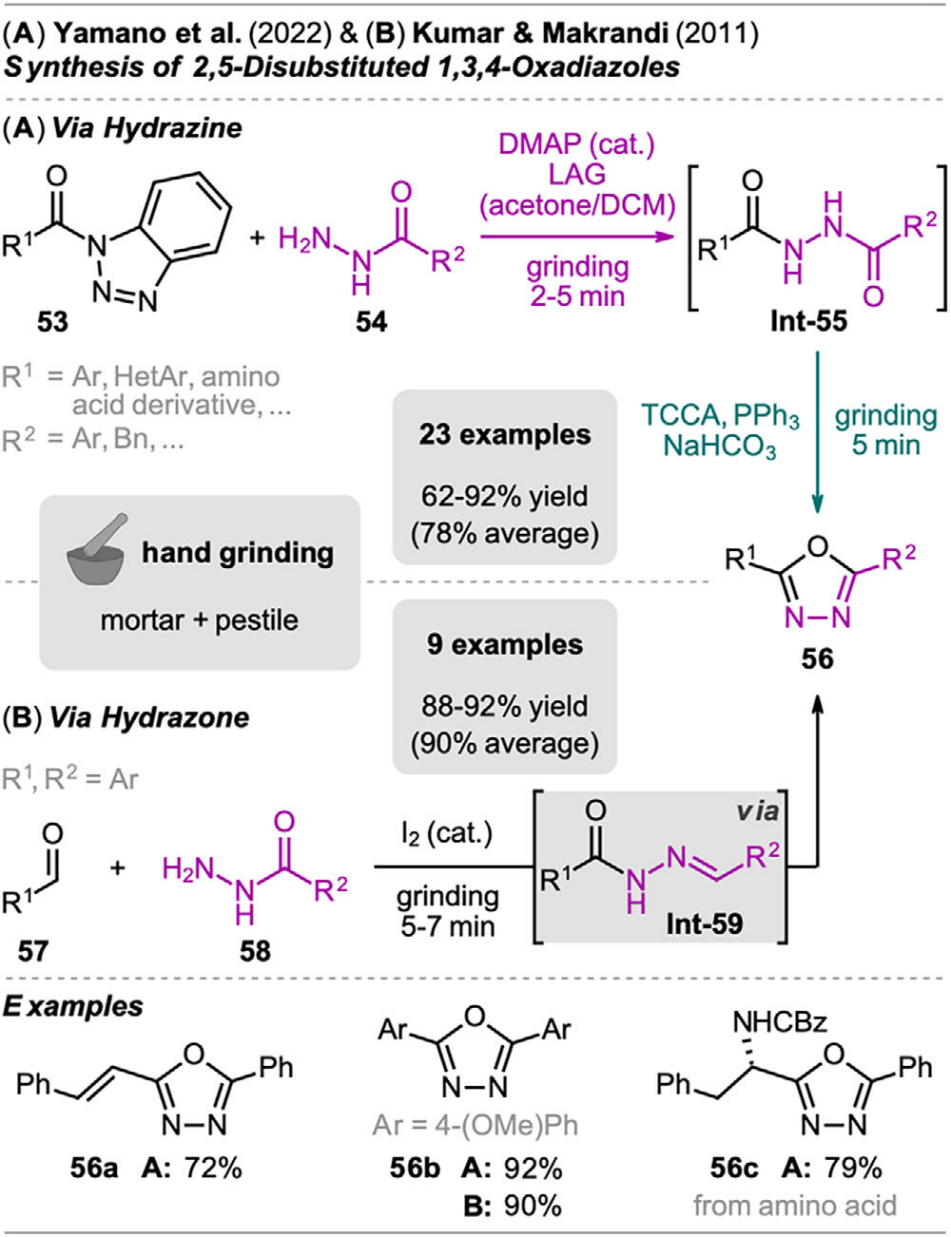
Synthesis of 2,5‐disubstituted 1,3,4‐oxadiazoles via (A) sequential hydrazine formation and intramolecular cyclization from *N*‐acyl benzotriazoles or via (B) a domino‐type hydrazone formation–cyclization sequence from aldehydes.

An approach in two‐steps for the one‐pot synthesis of 2,5‐disubstituted 1,3,4‐oxodiazoles via a hydrazine intermediate was reported by Yamano et al. in 2022 (Scheme 10A).^[^ ^76^ ^]^ Starting from *N*‐acyl benzotriazole **53** and aryl hydrazide **54**, hydrazine intermediate **Int‐55** was formed by grinding under LAG conditions and base catalysis using 4‐dimethylaminopyridine (DMAP). The 1,2‐diacylhydrazine **Int‐55** was then subsequently cyclized by the addition of trichloroisocyanuric acid (TCCA) and triphenylphosphine, acting as a cyclodehydration reagent. The 2,5‐disubstituted oxadiazoles **56** were obtained within short reaction times (max. 10 minutes in total) and in good to excellent yield after column chromatography. A wide range of activated ester substrates **53** were compatible with the presented reaction conditions. Interestingly, also Boc‐, Cbz‐, and Fmoc‐protected amino acid‐derived substrates **53** were successfully reacted without affecting the present protecting groups (e.g., compound **56c**).

Even though the method seems simple, the used *N*‐acyl benzotriazoles **53** are preactivated substrates, and most of the presented ones are not commercially available and need to be synthesized. The authors justify their choice for *N*‐acyl benzotriazoles due to easier handling and higher stability compared to acid chlorides, which are commonly used as precursors in the synthesis of oxadiazoles.^[^ ^77^ ^]^ However, an even easier grinding approach for the domino‐type synthesis of oxadiazoles, starting from aldehydes **57** (instead of acyl benzotriazoles) and aryl hydrazides **58** via hydrazone intermediates **Int‐59**, was already developed in 2011 by Kumar and Makrandi (Scheme 10B).^[^ ^78^ ^]^ In the presence of catalytic amounts of iodine, *N*‐acyl hydrazones **Int‐59** were formed in situ, which subsequently underwent oxidative cyclization to yield the desired oxadiazoles **56**. Comparing the two methods, the latter one benefits from readily available aldehyde starting materials. Nevertheless, the presented scope is limited to aryl aldehydes and aryl hydrazides, raising questions about the applicability of this protocol for the synthesis of more complex oxadiazoles as shown by Yamano et al.

**Scheme 11:**
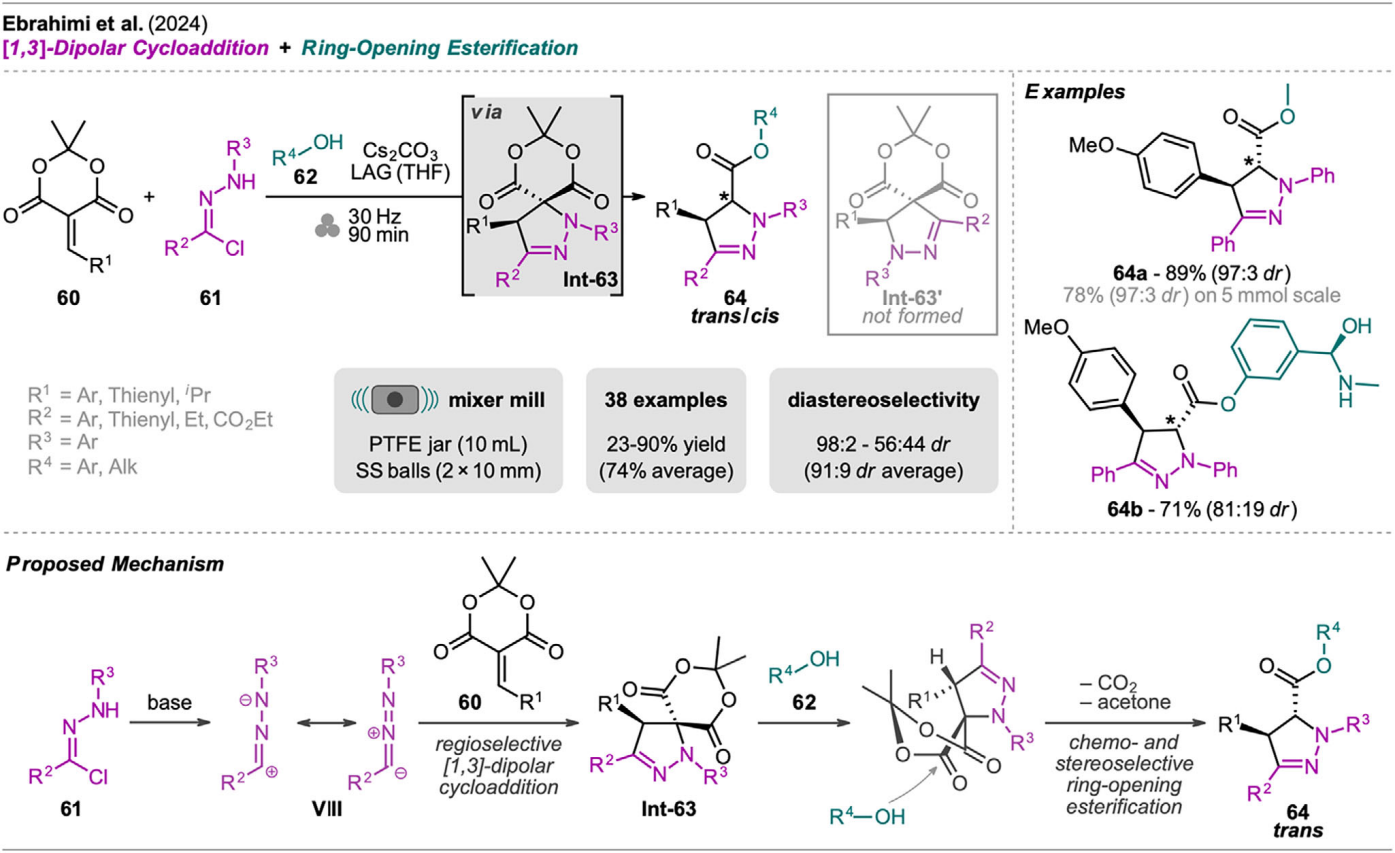
Synthesis of multi‐substituted Δ^2^‐pyrazolines via a domino‐type cycloaddition and ring‐opening esterification sequence.

In 2024, Ebrahimi et al. developed a one‐pot protocol for the stereoselective synthesis of multi‐substituted Δ^2^‐pyrazolines (Scheme 11).^[^ ^79^ ^]^ This synthetic pathway, in contrast to most of the presented protocols, does not require sequential addition of reagents but can be performed in one‐pot in a domino fashion in most cases. However, the route consists of two distinctive steps, namely a 1,3‐dipolar cycloaddition and ring‐opening esterification with an isolatable intermediate. In the first step of the reaction, alkylidene Meldrum's acid **60** reacts with the nitrilimine **VIII**, in situ generated from hydrazonyol chloride **61** in the presence of cesium carbonate, giving the Meldrum's acid functionalized Δ^2^‐pyrazoline **Int‐63**. The cycloaddition was found to proceed regioselective, strongly favoring the regioisomer where the more sterically hindering substituent of **61** (R^2^) occupies the 5‐position of the pyrazoline **Int‐63** over the second possible isomer with R^2^ in 3‐position **Int‐63′**, which was not observed at all. The alcohol **62** present in the reaction mixture then readily attacks this intermediate **Int‐63** and the ester‐functionalized pyrazoline **64** is formed by ring‐opening and release of CO_2_ and acetone. Cesium carbonate was found to participate in both steps, the conversion of hydrazonoyl chloride **61** into reactive nitrile imine and the ring‐opening of **Int‐63**, since two equivalents were necessary to achieve high conversion. Furthermore, the utilization of LAG with THF resulted in better yields with a total reaction time of 90 minutes. More importantly, the ring‐opening esterification proceeded with high chemo‐ and stereoselectivity in most cases, forming the *trans* product as the major one (up to 98:2 *dr trans*/*cis*).

The generality of this protocol was investigated with a series of (hetero)aryl‐ and alkyl‐substituted alkylidene Meldrum's acids **60**, where high selectivity for the *trans* isomer was observed in all cases. Among aryls, only the nitro‐substituted compound  failed, but aryls with other EWGs and EDGs reacted smoothly. Furthermore, various di‐aryl‐substituted hydrazone chlorides **61** were explored, giving the corresponding products in moderate to high yield and also high diastereoselectivity, except for *ortho*‐methyl substituted phenyl residues (56:44 *dr*). Regarding compound **61**, next to primary alcohols, phenols were also found to readily react in the esterification step with excellent diastereoselectivity, not disturbing the cycloaddition. When (*R*)‐(−)‐phenylephrine, which contains a phenolic OH, secondary OH, and amine group, was employed, only the phenolic hydroxyl group underwent the esterification–decarboxylation sequence, while other functionalities remained intact (example **64b**). With 2‐aminoethanol, preformation of **Int‐63** was necessary by milling of all reagents except the alcohol, which was subsequently added to the jar followed by a second milling step. Additionally, a scale‐up experiment (25‐fold, 5 mmol) for one example **64a** was included, giving Δ^2^‐pyrazoline with slightly diminished yield (78% vs. 89%) but no change in diastereoselectivtiy (97:3 *dr*).

For comparison, the reaction was also conducted as a one‐pot solution‐based process in THF at 50°C for 90 minutes, which resulted in lower yields and selectivity for most examples. The two methods were evaluated for their environmental impact by calculating green metrics, including AE, RME, E‐factor, Mol. E, and EcoScale. While both approaches have the same AE, the MC process exhibits a better RME due to enhanced yield. Additionally, the E‐factor highlighted the advantages of the MC protocol with the reduced solvent amount as the main contributing factor (5.1 vs. 26.6), though without consideration of solvents needed for isolation and purification. The EcoScale further emphasized the benefits of ball milling over solution‐based chemistry with a notably higher value for the MC approach (MC: 54 vs. SC: 29.5). Together, these metrics underscore the superior greenness of the MC process in this case.

### 2.4 Other Heterocyclic Scaffolds

**Scheme 12:**
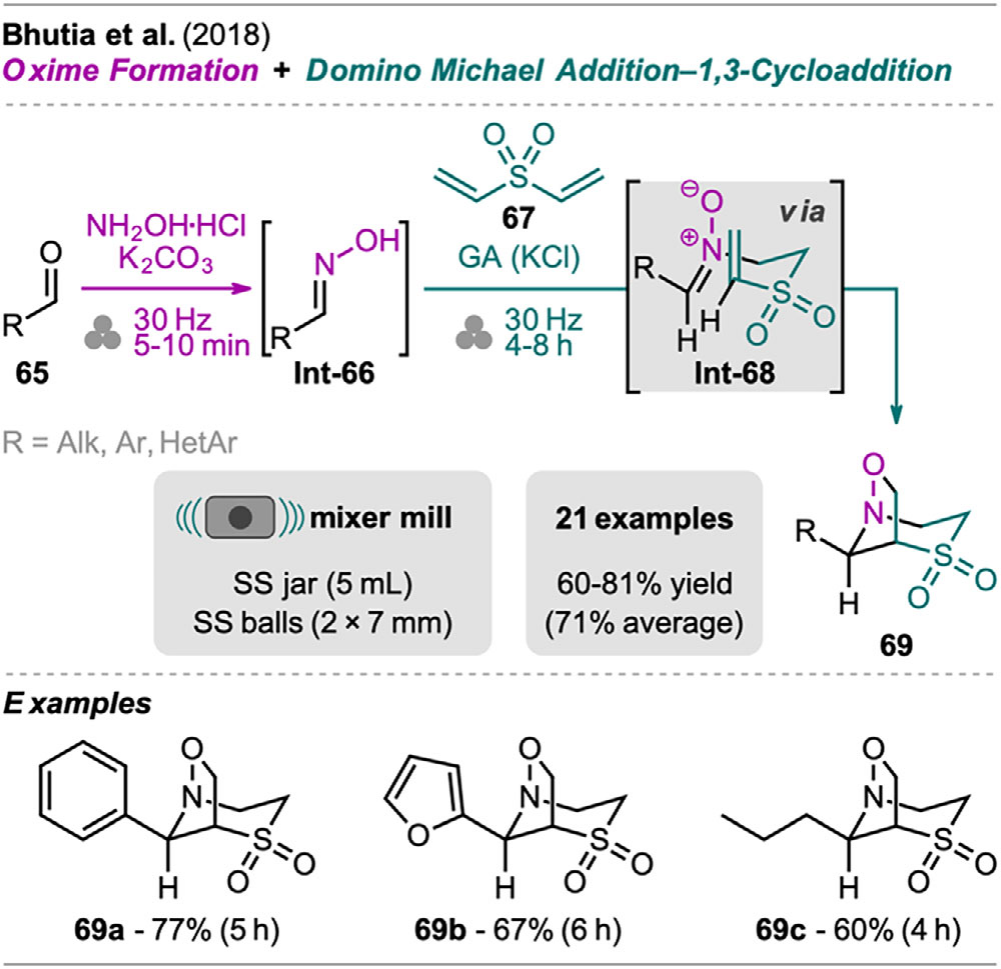
Synthesis of bridged bicyclo azasulfone derivatives via an oxime formation and domino Michael addition‐1,3‐cycloadditon sequence.

An efficient mechanochemical protocol yielding bridged bicyclo azasulfone derivatives from aldehydes and divinyl sulfones was developed by Bhutia et al. (Scheme 12).^[^ ^80^ ^]^ Starting the synthesis sequence, aldehyde **65** was transformed into the corresponding oxime **Int‐66** by milling with hydroxylamine hydrochloride and potassium carbonate. In one‐pot, divinyl sulfone **67** was added together with potassium chloride as GA, and further milling resulted in the Michael addition product **Int‐68** which subsequently underwent regioselective 1,3‐cycloaddition to form the desired product **69**. The applicability of the developed methodology was investigated with various alkyl, aryl, and heteroaryl aldehydes **65**. The reaction proceeded with similar efficiency independent of EDGs or EWGs present on the aromatic ring, and also aliphatic aldehydes of different chain lengths were suitable as starting materials. Reaction times were optimized for each substrate and in the range of 4–8 hours and **69** products were obtained in good yields.

Unfortunately, the authors do not provide data for green metrics but draw comparisons to other methods in solution for the synthesis of **69a** obtained from benzaldehyde (R  =  Ph). Noteworthy, previous reported protocols either in xylene without catalyst at 140°C by Grigg et al.^[^ ^81^ ^]^ or water with dodecylbenzenesulfonic acid, a surfactant, by Hota et al.^[^ ^82^ ^]^ started from the oxime **Int‐66** and only the domino Michael addition–cycloaddition was performed. Although the solution‐based reactions were performed in isolated fashion, the obtained yields for **69a** were significantly lower compared to the MC protocol (SC: 60 and 57%, respectively, vs. MC: 77%), which includes also the prior formation of the oxime **Int‐66a** in one‐pot. This clearly indicates that the MC approach is superior to the presented SC methods. Even though CC was necessary for the purification of the products, which is a solvent‐intensive step, the contribution by Grigg et al.^[^ ^81^ ^]^ reports purification via recrystallization on a 10 mmol scale which might also be applicable for the MC process when performed on a larger scale. However, screening for greener solvent alternatives would be required since benzene was reported as a suitable solvent for recrystallization.

In addition, the authors conducted an in vitro study to assess the potential antibacterial activity of the synthesized bridged bicyclic sulfone compounds. Some of the compounds demonstrated significant inhibitory effects against *Myobacterium smegmatic* and *Escherichia coli*. However, none of the investigated compounds exhibited activity against *Stapholyococcus aureus*. These preliminary findings highlight the potential of this compound class as novel antibacterial agents and warrant further evaluation.

**Scheme 13:**
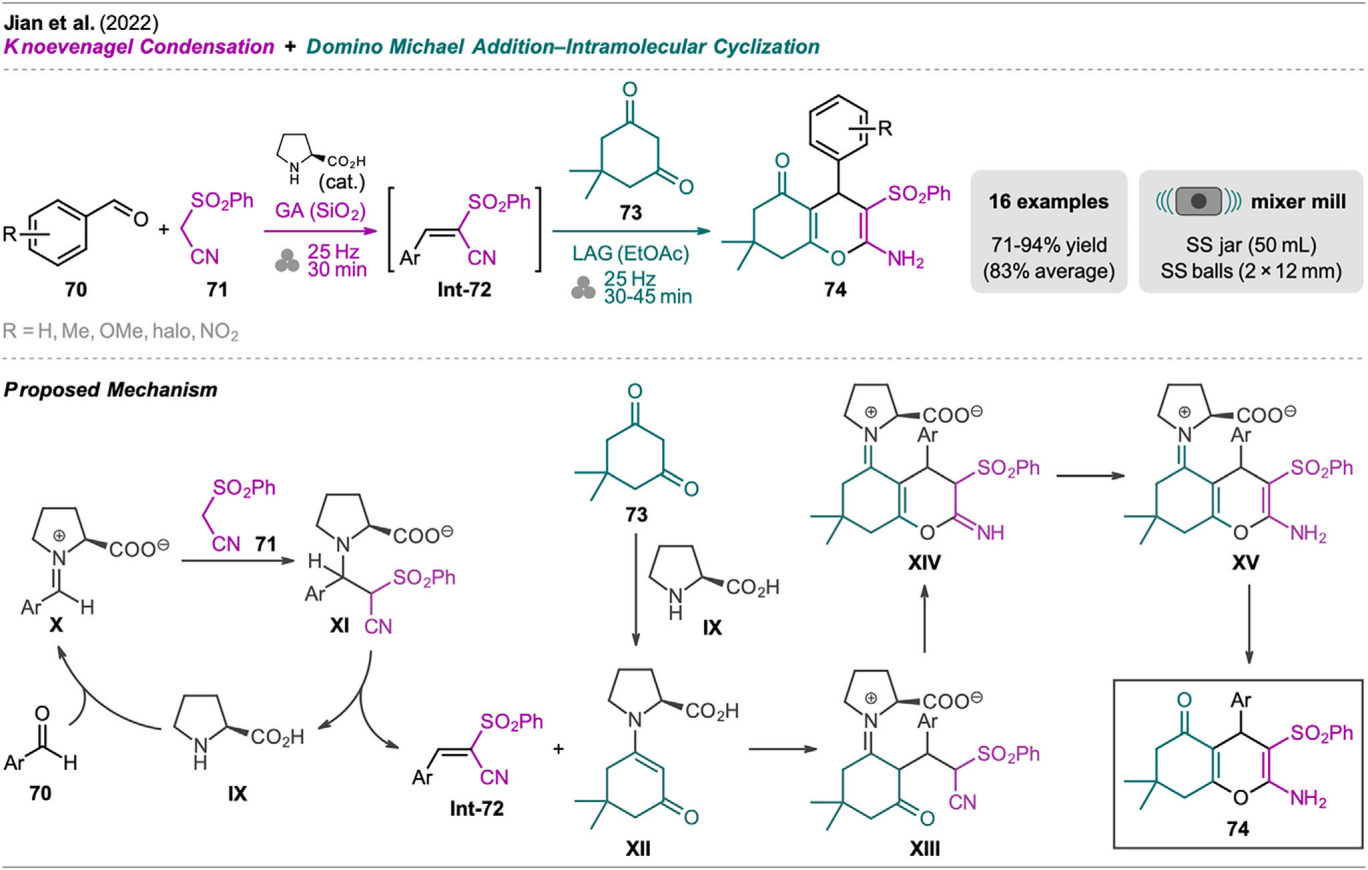
Synthesis of arylsulfonyl 4*H*‐pyran derivatives via sequential Knoevenagel condensation and domino Michael addition‐intramolecular cyclization.

**Scheme 14:**
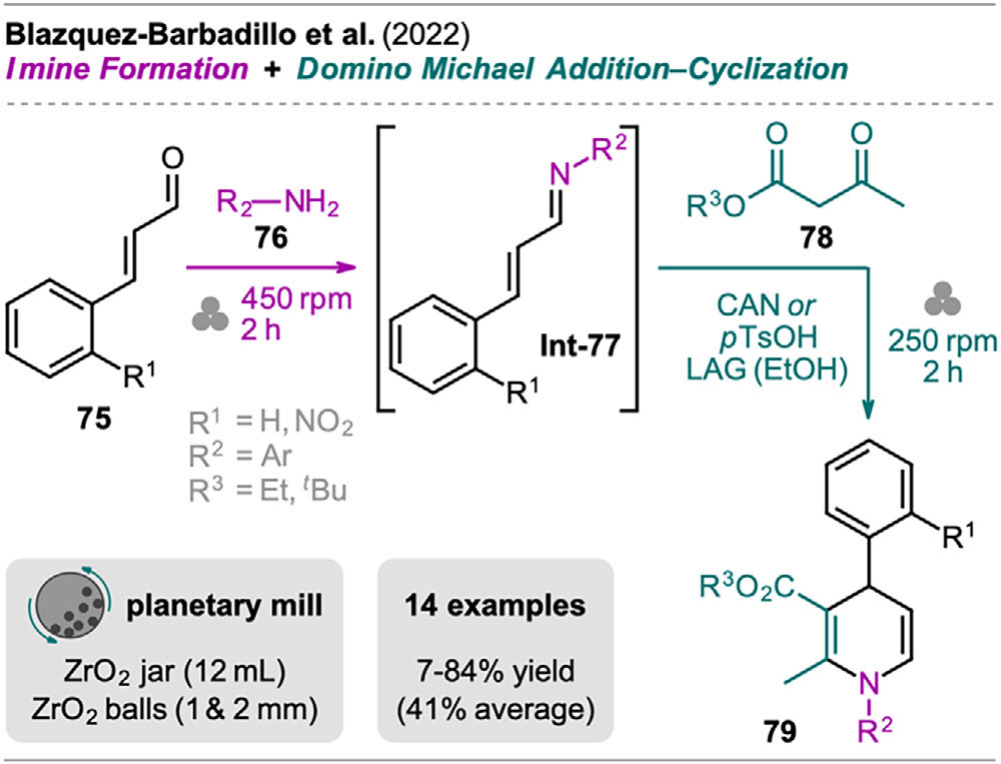
Synthesis of unsymmetrical 1,4‐diaryl‐1,4‐dihydropyridines via an imine formation and domino Michael addition–cyclization sequence.

In 2022, Jiang et al. reported a mechanochemical protocol for the synthesis of arylsulfonyl 4*H*‐pyrans in two sequential steps (Scheme 13, 14).^[^ ^83^ ^]^ This approach uses l‐proline as a catalyst, which participates in both steps, catalyzing the Knoevenagel condensation and activating dimedone **73** in situ for the subsequent Michael addition and intramolecular cyclization. The authors describe the synthesis using a mixer mill and performed the reaction in a one‐pot, two‐step fashion. First, aldehyde **70** and sulfonyl acetonitrile **71** were milled in the presence of the organocatalyst with silica as GA. The Knoevenagel condensation product **Int‐72** then underwent Michael addition with added dimedone **73** in its activated enamine form **XII**, which was generated in situ. The use of EtOAc as a LAG additive, added in the second step, resulted in higher yields of the 2‐amino‐3‐arylsulfonyl 4*H*‐pyran product **74**. Operation as a multicomponent reaction was not investigated further under these conditions, as initial experiments were unsuccessful and desired product **74** was only obtained when the two steps were performed sequentially.

Based on the obtained results, the authors proposed a plausible mechanism for the reaction sequence. First, aldehyde **70** undergoes condensation with l‐proline (**IX**) forming iminium ion **X** which reacts with **71** to form intermediate **XI** and results in **Int‐72** upon elimination of **IV**. This then reacts with enamine **XII** formed by condensation of dimedone **73** and l‐proline (**IX**) in a Michael addition giving intermediate **XIII**. Subsequent intramolecular cyclization (intermediate **XIV**) and tautomerization result in iminium ion **XV** which upon hydration gives desired product **74**.

In the substrate scope, various aryl aldehydes **70** with both EWGs and EDGs in different positions afforded products **74** in good to excellent yields. The practicability of the developed protocol was demonstrated in a scale‐up experiment (20‐fold, 5 mmol) starting from benzaldehyde (**70**, R = H), giving the desired product albeit with significantly lower yield (61% vs. 92% on a 0.25 mmol scale).

Additionally, a study on the reutilization of used l‐proline was performed. The catalyst was shown to be easily recoverable by filtration of the crude product as a solution in dichloromethane and was reused after evaporation in another reaction cycle after drying. Even after three recycling steps of l‐proline, the reaction still proceeded with a 66% yield compared to 92% with a fresh catalyst. These results further emphasize the method's potential for minimizing waste and enhancing sustainability compared to solution‐based protocols.

A solvent‐free synthetic pathway toward 1,4‐dihydropyridines (1,4‐DHPs), a scaffold present in various therapeutic agents used in medicine for the treatment of cardiovascular diseases,^[^ ^84^, ^85^ ^]^ has been presented by Blazquez‐Barbadillo et al. in 2022 (Scheme 14).^[^ ^86^ ^]^ As the first step, cinnamaldehyde **75** was milled with amine **76** in a planetary without additives to form the imine intermediate **Int‐77**. Subsequently, ceric ammonium nitrate (CAN) or *p*TsOH was added in catalytic amounts (15 mol%) together with β‐ketoester **78**, and further milling at a lower frequency compared to the first step resulted in 1,4‐DHP **79**. In general, the authors reported difficulties in the isolation of the final products. For amines **76** where R^2^ is a (substituted) phenyl group, purification via recrystallization was not possible due to the oily nature of the products, requiring CC. However, the isolated yields were only low or moderate, ranging from 7 to 50%, due to CAN‐promoted aerobic overoxidation to pyridines in the workup and purification process, despite clean conversion to the desired 1,4‐DHPs confirmed by GC‐MS. When the same reactions were performed with *p*TsOH, isolation of the corresponding 1,4‐DHPs via CC was unsuccessful, and the formation of an unidentified byproduct was observed. For solid 1,4‐DHPs, such as those synthesized from 2‐nitro cinnamaldehyde and 1‐naphthylamine, the use of *p*TsOH instead of CAN as a catalyst allowed isolation via precipitation from water, resulting in good isolated yields of over 80%. Therefore, the choice of catalyst was made based on the physical state of the targeted product.

Regarding the substrate scope, nitro‐substituted cinnamaldehyde resulted in slightly better yields compared to unsubstituted cinnamaldehyde. Various aniline components **76** were investigated, and better results were obtained with EDGs on the phenyl ring due to enhanced nucleophilicity of the amino group.

The authors compared the presented mechanochemical method to solution‐based approaches showing that yields were significantly higher for the latter. Furthermore, a similar solution process only required one hour of reaction time, lower amounts of CAN (5 mol%), and could be performed as a multicomponent reaction in a single operational step.^[^ ^87^ ^]^ Noteworthy, single‐step operation was also investigated under mechanochemical conditions, however resulted in lower reaction efficiency compared to the sequential approach. Nevertheless, the presented method was assessed regarding its greenness using the web‐based scoring matrix DOZN tool,^[^ ^88^ ^]^ which performs quantitative assessment of the greenness against the 12 Principles of Green Chemistry, categorizing them into three groups. For the groups “improved resource use” and “reduced human and environmental hazards,” the ball milling method exhibited lower (better) values compared to the solution‐based process,^[^ ^87^ ^]^ mainly due to the lower amount of ethanol used in the synthesis. For the group “increased energy efficiency,” both methods resulted in zero scores as the reactions were conducted under ambient conditions without requiring heating or higher pressure.

While the mechanochemical approach for the synthesis 1,4‐DHPs exhibited lower yields compared to solution‐based methods, it demonstrated advantages in terms of sustainability, particularly in reduced solvent usage and improved resource efficiency. Despite its limitations, the method represents a promising alternative for environmentally conscious synthetic strategies.

### 2.5 Bis‐Heterocyclic Scaffolds

**Scheme 15:**
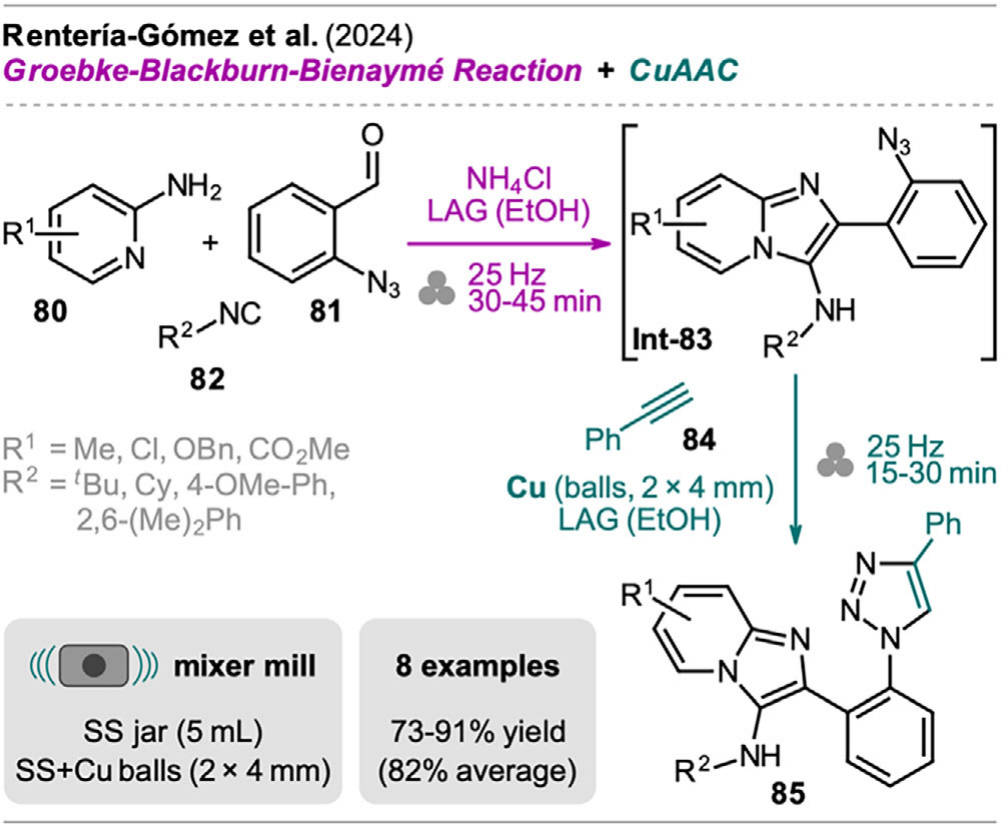
Synthesis of imidazo[1,2‐*a*]pyridine scaffolds containing a 1,2,3‐triazole unit via a multicomponent Groebke‐Blackburn‐Bienaymé reaction and copper‐catalyzed azide‐alkyne cycloaddition (CuAAC) sequence.

A mechanochemical one‐pot approach for the synthesis of imidazo[1,2‐*a*]pyridine scaffolds containing a 1,2,3‐triazole unit has been recently reported by Rentería‐Gómez et al. via subsequent Groebke‐Blackburn‐Bienaymé reaction (GBB‐3C), an isocyanide‐based multicomponent reaction, and copper‐catalyzed azide‐alkyne cycloaddition (CuAAC) (Scheme 15).^[^ ^89^ ^]^ Imidazo[1,2‐*a*]pyridines are of great pharmacological interest due to various biological activities, for example, anti‐cancer, anti‐mycobacterial, anti‐viral, and anti‐diabetic activity.^[^ ^90^ ^]^ The investigations on a mechanochemical GBB‐3CR version were initiated by previous work from the same group where this three‐component reaction was presented in a solvent‐ and catalyst‐free ultrasound‐assisted fashion in a one‐pot GBB‐3C/S_N_2/ring‐chain azido‐tautomerization sequence to synthesize various bis‐heterocycles.^[^ ^91^ ^]^ The mechanochemical sequence toward imidazo[1,2‐*a*]pyridines **85** started with the GBB‐3CR of 2‐aminopyridine **80**, 2‐azidobenzaldehyde (**81**), and isocyanide **82** in equimolar amounts with ammonium chloride as a catalyst under LAG conditions using EtOH. The addition of ethanol was found to be crucial to obtain the GBB‐3CR product **Int‐83** at all. After complete consumption of the starting materials, CuAAC of the azide moiety was performed sequentially with phenylacetylene (**81**), catalyzed by elemental copper from copper balls that were also added to the milling jar. Again, the addition of EtOH as a LAG additive was crucial for a successful outcome. Noteworthy, the CuAAC step could also be performed with sodium ascorbate and copper sulfate instead of copper balls, however requiring increased reaction time. With this protocol in hand, a series of bis‐heterocycles **85** were synthesized in good to excellent yield (73‐91%) in one‐pot. Different alkyl and phenyl isocyanides **82** were explored, showing better efficiency for alkyls. Various substituted 2‐aminopyridines **80** were also found to be tolerated, however, when 6‐bromo‐2‐aminopyridine was reacted, no GBB‐3CR product was obtained.

Notably, the same sequence toward **80** was additionally performed assisted by ultrasound irradiation (USI), however, the GBB‐3CR required external heating, and the CuAAC using sodium ascorbate and copper sulfate was performed in *^t^* BuOH/H_2_O mixture. Furthermore, the two steps could not be performed in one‐pot and evaporation of ethanol used as a solvent in the GBB‐3CR, even though used in small amounts, was necessary in between. The mechanochemical protocol is therefore preferred over the sonochemical‐assisted process due to no requirement for external heating, easier handling of intermediates, and shorter overall reaction times (approx. 1 hour vs. 5 hours) while the achieved yields are comparable.

In addition, the authors calculated various green metrics values including AE, E‐factor, RME, PMI, and CE. All given values except for the PMI are the same for the MC‐ and USI‐assisted process with high AE of >95 and low E‐factors of 0.14, for example. However, calculation only takes stoichiometric reagents into account. Catalysts and solvents used in the reaction but also for isolation and purification are left out and considered as recyclable. For the calculation of the PMI, solvents that are needed in the transformation are considered, resulting in a lower PMI value for the MC process (2.25) compared to the USI process (11.10). According to this, the MC one‐pot process was identified as the greener and more sustainable method, nevertheless, the calculations have been performed over‐optimistically in regard to catalyst and solvent recycling.

**Scheme 16:**
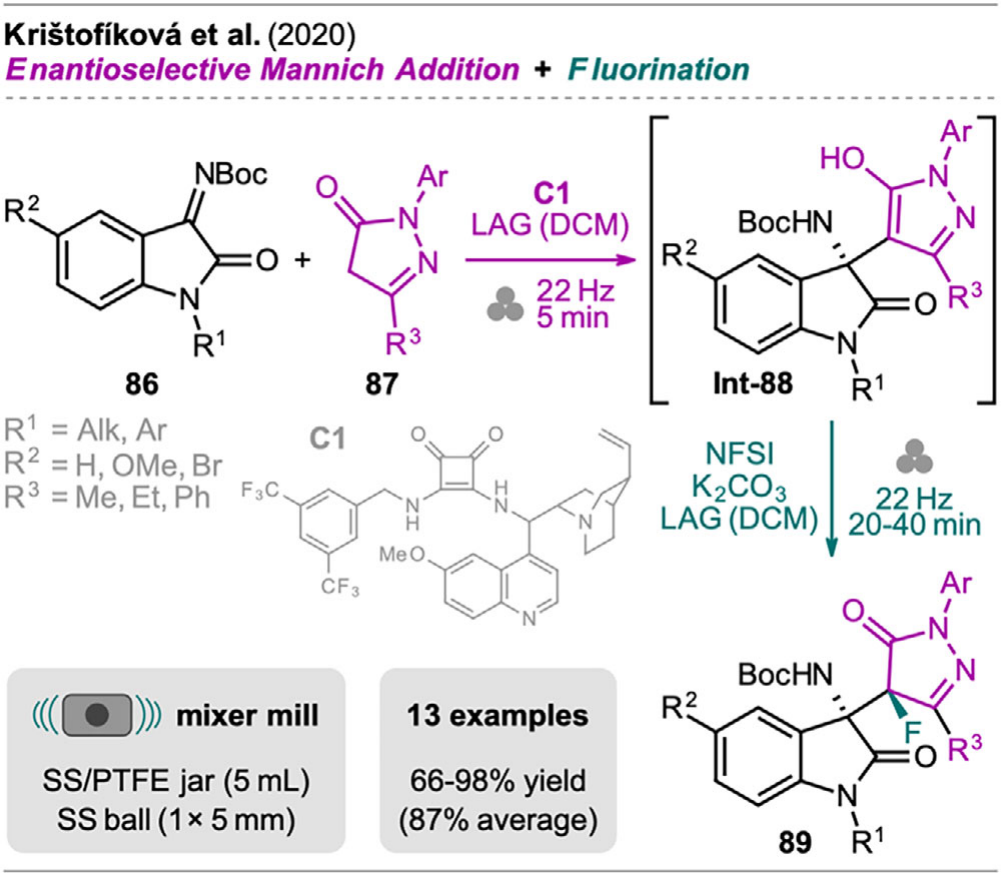
Synthesis of fluorinated oxoindole−pyrazolone adducts via sequential enantioselective Mannich addition and fluorination.

Krištofíková et al. demonstrated a mechanochemical protocol for the synthesis of bis‐heterocyclic fluorinated oxoindole−pyrazolone adducts (Scheme 16).^[^ ^92^ ^]^ This approach combines the enantioselective Mannich addition of oxindole imine **86** to a pyrazolone **87** with subsequent diastereoselective fluorination at the pyrazolone ring in a one‐pot process. The protocol builds on earlier work by Song and coworkers who developed this transformation under similar reaction conditions in a one‐pot fashion but as a solution‐based reaction in dichloromethane (DCM).^[^ ^93^ ^]^ By transitioning to a mechanochemical approach, Krištofíková et al. aimed to shorten reaction times and eliminate the use of chlorinated solvents, setting the stage for a more sustainable and efficient synthetic protocol. Focusing on the MC protocol, the Mannich addition proceeded with high enantioselectivity using the quinine‐derived squaramide catalyst **C1**, and **Int‐88** was observed after a short reaction time of only 5 minutes. A similar catalyst was reported in the solution‐based approach; however, better selectivity and yield were observed with **C1** in the MC protocol. By adding *N*‐fluorobenzenesulfonimide (NFSI) and potassium carbonate, the electrophilic fluorination of **Int‐88** was accomplished, giving product **89** in good to excellent yields with excellent diastereoselectivity. Minute amounts of DCM were still necessary to achieve high yield and selectivity, and attempts to replace DCM with less environmentally harmful organic solvents (e.g., EtOAc or acetonitrile) resulted in lower efficiency. Nevertheless, the MC approach reduced the total reaction time to 25–45 minutes, compared to 2 hours for the SC protocol (1 hour for each step). In terms of catalyst loading and reagent excess, the solution protocol required only 0.5 mol% of catalyst, compared to 1 mol% (MC), while the quantities of NFSI and K_2_CO_3_ used were identical. Still, the ball milling approach was identified as the more sustainable process with a lower PMI of 4.1 compared to 42.2 for the solution process, primarily due to the reduced solvent usage.

In addition to pyrazolones **87**, Krištofíková et al. explored isoxazolones to construct bis‐heterocyclic fluorinated oxindole−isoxazolone adducts (not shown). However, under the optimized conditions for pyrazolones, the reaction proceeded with significantly lower efficiency and selectivity. Efforts to improve this reaction by testing of various catalysts, increasing the amount of isoxazolone and NFSI, prolonging the reaction time, etc., resulted in only marginal improvements.

**Scheme 17:**
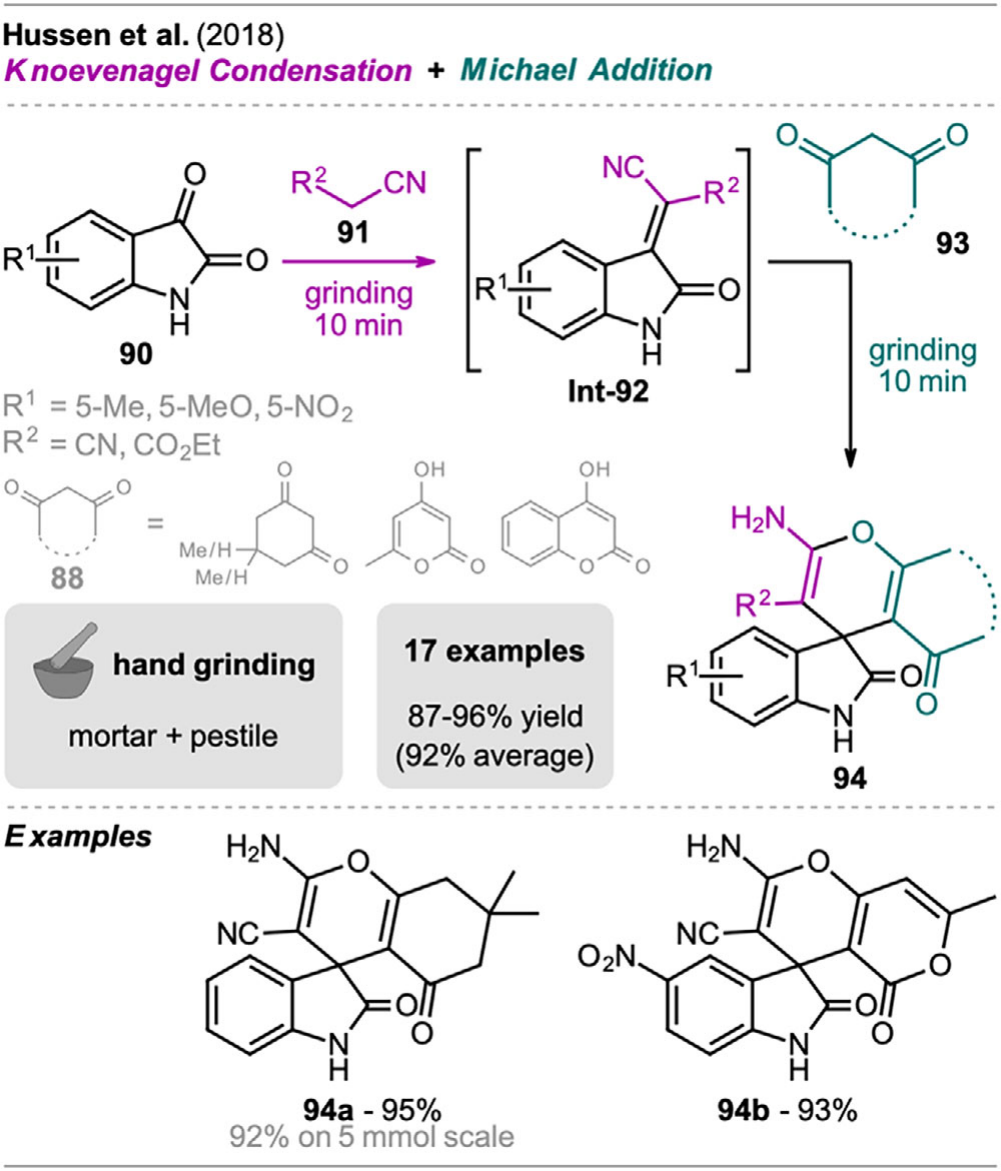
Synthesis of spiro[4H‐pyran oxoindolines] derivatives via a Knoevenagel condensation and Michael addition sequence.

Hussen et al. described a stepwise one‐pot synthesis of spiro[4*H*‐pyran oxoindolines] under neat grinding conditions, without the use of catalyst or additive, via a Knoevenagel condensation‐Michael addition sequence (Scheme 17).^[^ ^94^ ^]^ In contrast, a similar approach was previously reported by Elinson et al. in a multicomponent fashion with NaOAc as a catalyst for the activation of malononitrile.^[^ ^95^ ^]^ However, in the work by Elison et al., only reactions of isatins with malononitrile and dimedone were demonstrated. In Hussen et al.’s protocol, isatin **90** was ground with malononitrile or ethylcyanoacetate **91**, giving the Knoevenagel condensation product **Int‐92** within just 10 minutes. This was followed by the addition of a cyclic 1,3‐dicarbonyl compound **93** which underwent Michael addition with **Int‐92**, yielding the spiro compound **94**. This straightforward method allowed the synthesis of various spiro[4*H*‐pyran oxoindolines] **94**, including various isatins with different substituents and 1,3‐dicarbonyl substrates, all with excellent yields. The practicability of the method was also demonstrated with a scale‐up experiment (10‐fold, 5 mmol), which provided the product in similar high yield. Notably, pure products were obtained by washing the reaction mixture with water and no CC was necessary. Furthermore, attempts to perform the reaction in multicomponent fashion without preformation of the Knoevenagel condensation product **Int‐92** were unsuccessful, resulting in a mixture of unidentifiable products.

To evaluate the greenness of the method, green metrics including the AE, PMI, CE, and RME, as well as the E‐factor were calculated for the scale‐up experiment (92% yield). As no additives and solvents are required and reactants are used in equimolar amounts, the method exhibits an excellent E‐factor of 0.05 and RME of 95%. Moreover, the AE and CE are remarkably high with values of 95% and 94.9%, respectively. These results clearly underscore the efficiency and sustainability of the developed method.

**Scheme 18:**
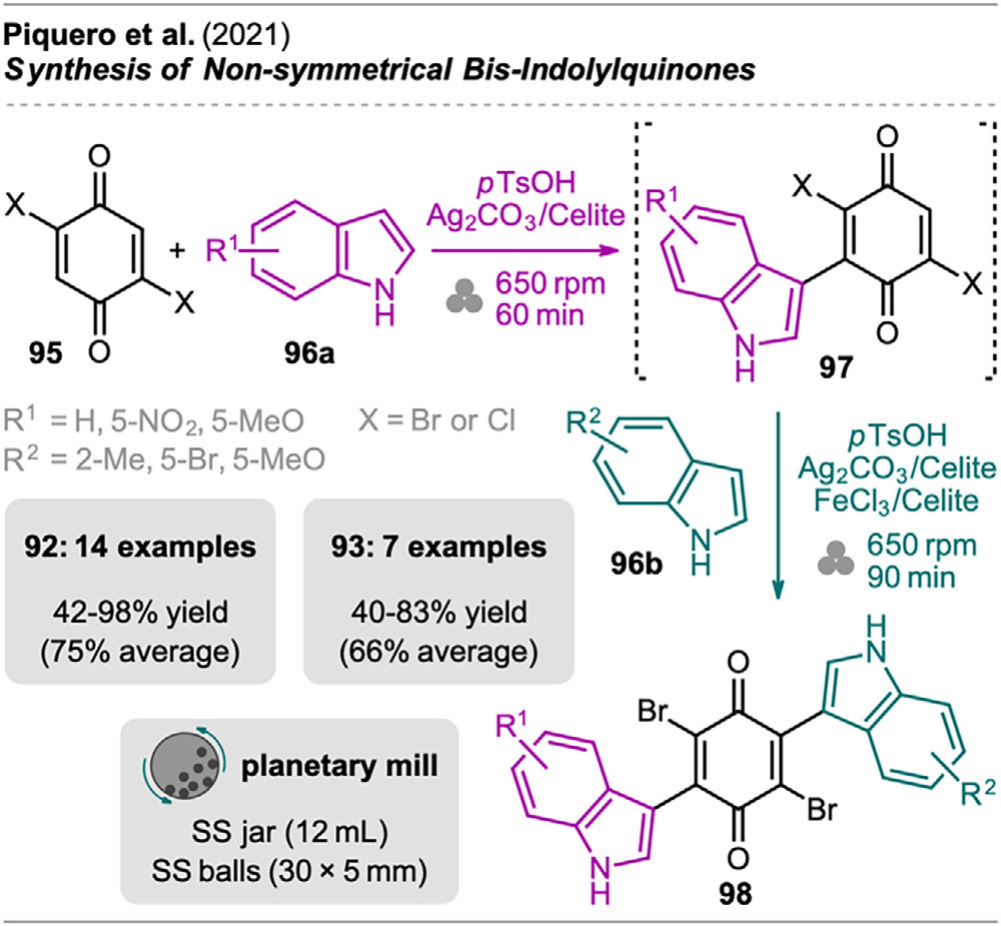
Synthesis of mono‐indolylquinones and sequential one‐pot transformation into (non)symmetrical bis‐indolylquinones.

Bis‐indolyl quinones are secondary fungal metabolites that belong to a class of compounds exhibiting various biological activities (e.g., anti‐HIV, antitumor, and antidiabetic).^[^ ^96^, ^97^ ^]^ The synthesis of nonsymmetric bis‐indolylquinones often requires isolation of the mono‐indolylquinones, and many solution‐based methods have been developed using this approach.^[^ ^97^, ^98^, ^99^ ^]^ Piquero et al. reported a mechanochemical approach that allows the one‐pot synthesis of both symmetrical and nonsymmetrical bis‐indolylquinones (Scheme 18).^[^ ^100^ ^]^ For nonsymmetrical bis‐indolylquinones, mono‐indolylquinone **97** was first obtained by milling dibromobenzoquinone **95** and indole **96a** with *p*TsOH and Fertizon's reagent (Ag_2_CO_3_ on Celite) as the oxidant in a planetary mill. Then, the second indole **96b** was added together with another portion of *p*TsOH and oxidant as well as iron(III) chloride on Celite or cerium ammonium nitrate (the latter for methoxy‐substituted indoles) as catalyst. Without the Lewis acid, the second addition proceeded sluggishly. The bis‐indolylquinones **98** were isolated in moderate to good yields, with no requirement for CC. Filtration of a solution in EtOAc, washing with water, and precipitation from solvent was sufficient for purification.

The mono‐indolylquinones **97** could also be isolated, and both dibromoquinone and dichloroquinone gave comparable yields. For symmetrical bis‐indolylquinones (R^1^ = R^2^), the two additions were achieved in a single step under the same conditions used for the second step in the sequential approach toward nonsymmetrical bis‐indolylquinones (R^1^ ≠ R^2^).

The authors compared their method for symmetrical bis‐indolylquinones to a solution‐based protocol but did not extend this comparison to the one‐pot two‐step process for nonsymmetrical ones. However, the one‐pot synthesis of symmetrical **98** uses the same equivalents of reagents and substrates, and the values are expected to remain in a similar range. The mechanochemical approach offers clear advantages due to its one‐pot design, eliminating the need for two separate isolation and purification steps, which usually require significant solvent use. Both methods demonstrated excellent values for the AE (>99%) as no side products are formed. The sustainability of the mechanochemical process was evaluated by comparing the calculated E‐factor (55.4) and PMI (56.4) to the values for the solution‐based protocol (E‐factor: 3291, PMI: 3292).^[^ ^99^ ^]^ It has to be mentioned that the paper does not provide details on the calculations for both processes.

### 2.6 Modification/Substitution of Heterocyclic Compounds

**Scheme 19:**
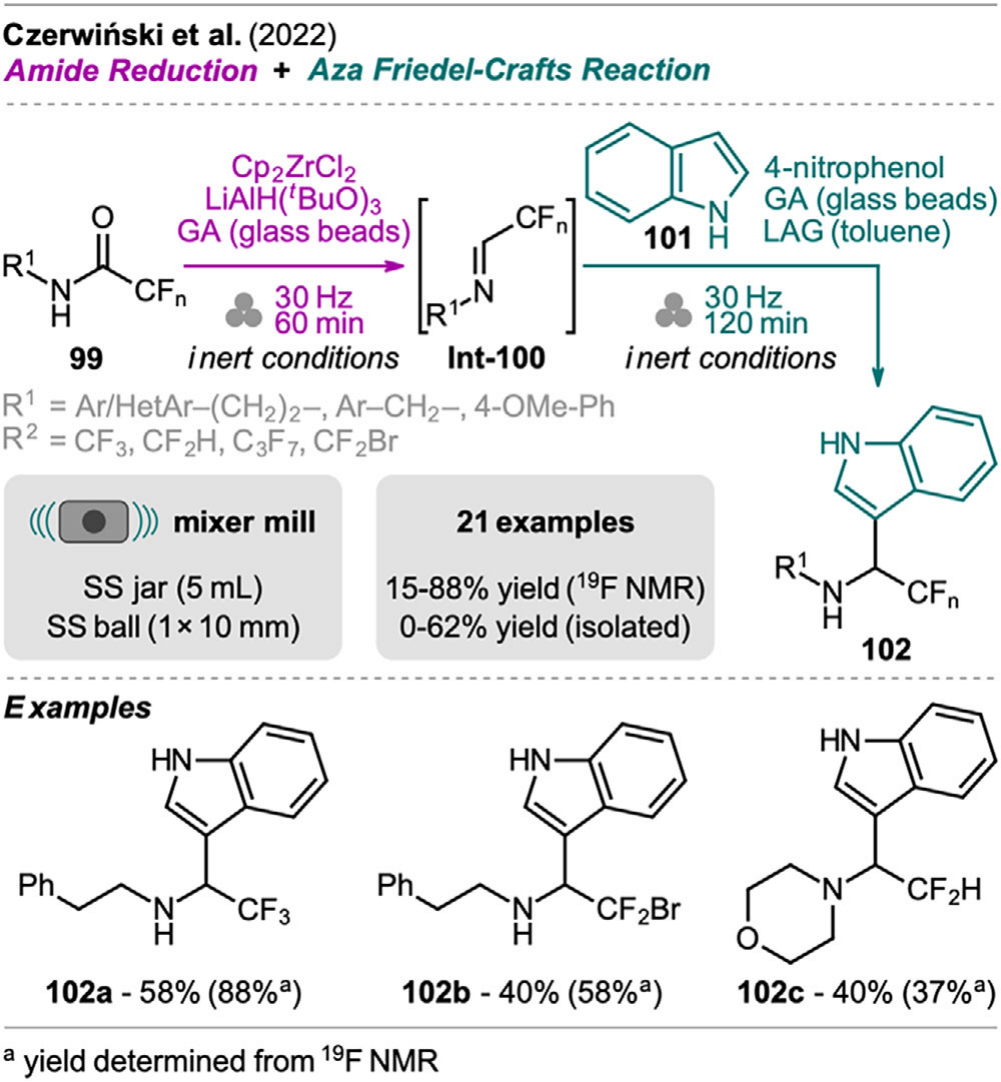
Synthesis of indole‐functionalized, fluorinated amines via sequential reduction of fluoroacetamides and aza Friedel‐Crafts reaction.

To contribute to the development of sustainable methods in modern organic synthesis, Czerwiński et al. investigated the mechanochemical one‐pot partial reduction of fluoroacetamides and sequential nucleophilic addition of indoles to obtain high‐value functionalized fluorinated amines (Scheme 19).^[^ ^101^ ^]^ A solution‐based approach for this reaction in THF was presented by the same group in 2019.^[^ ^102^ ^]^ Performing the reduction and nucleophilic addition step in one‐pot in solution, Schwartz's reagent (Cp_2_Zr(H)Cl) was identified as the only suitable reducing agent that allowed the combination of the two steps. However, the addition step of indole in the presence of TFA required long reaction times (16 hours). With the MC approach, the authors succeeded in translating this wet synthesis process to a more sustainable, greener solid‐state reaction. In the MC protocol, the reduction of fluoroacetamide **99** to the imine intermediate **Int‐100** was performed using Schwartz's reagent that was in situ formed from Cp_2_ZrCl_2_ with an excess of LiAlH(O*^t^* Bu)_3_. As GA, soda‐lime glass beads were used and **Int‐100** obtained within 60 minutes (SC: 15–45 minutes). Sequentially, an excess of indole **101**, 4‐nitrophenol (activator), and further glass beads were added together with a small amount of toluene as LAG additive. After further milling for 2 hours product **102** was obtained. Noteworthy, the reaction was performed under inert conditions and the jar was loaded and reopened in a glovebox. The presence of glass beads was found to be beneficial for the reaction outcome since they in general adsorb small amounts of water from atmospheric moisture on their surface which supports the liberation of free imine from its zirconia cycle. Other acids as activators for the aza Friedel‐Crafts reaction instead of 4‐nitrophenol resulted in the formation of various fluorinated by‐products. Another key step in the optimization of the reaction conditions in solid‐state was the switch from a planetary mill to a mixer mill. This shows that the applied mode of milling has a significant influence on the reaction outcome.

In addition, the authors also aimed for a more sustainable, easier purification method. Instead of CC, the amine products **102** were purified over an acidic resin (Dowex 50 W). We do not want to go into detail here, but this method turned out to be quite challenging due to the acid‐driven formation of an indole dimer from the product (not shown) and was not always sufficient. Furthermore, the reported isolation process was time‐intensive (>32 hours) and required various solvents (THF, H_2_O, DCM, and MeOH). Also, prior separation of excess nitrophenol was necessary by filtration over Celite. However, products could not be isolated in high purity by just performing CC.

The applicability of the new method was demonstrated with various (hetero)arylethyl amides (R^1^ = (Het)Ar‐(CH_2_)_2_‐), benzylic amide, and tertiary amide from morpholine. However, yields were only moderate, except for phenylethyl acetamide yielding **102a** (88% yield from NMR, 58% isolated yield). Variation of the fluoro group was shown with CF_3_, CF_2_H, C_3_F_7_, and CF_2_Br giving the corresponding amines in moderate yields. For many substrates, the isolated yields after purification over acidic resin are significantly lower compared to the yields determined based on quantitative ^19^F NMR, especially for amide substrates with EWGs at the aryl ring.

In general, the efficiency and applicability of the mechanochemical protocol seem questionable. However, comparing green metrics (E‐factor, EcoScale), the solid‐state process still seems to be slightly superior to the solution‐based approach regarding greenness.^[^ ^102^ ^]^ The authors calculated the E‐factors in two variants: without and with consideration of solvents used for isolation and purification. In both cases, the MC process results in lower values (without: 106; with: 5861) compared to the SC process (without: 2666; with: 8894). However, an E‐factor of 106 is still high for a milling approach and on the verge of satisfaction from a green chemistry point of view. This is also reflected in the EcoScale which is 47 for the MC process (SC: 36).

**Scheme 20:**
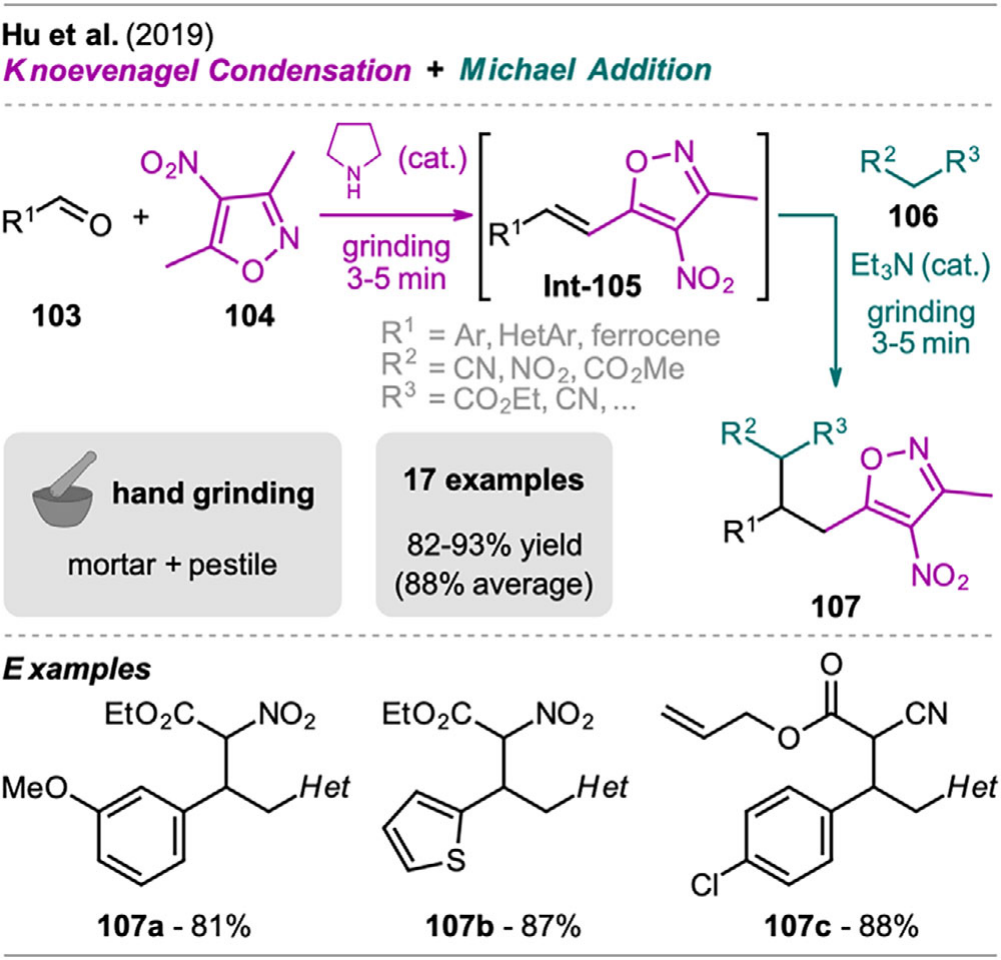
Synthesis of highly functionalized 3,4,5‐isoxazoles via a Knoevenagel condensation and Michael addition sequence.

In a one‐pot two‐step grinding approach, Hu et al. synthesized highly functionalized 3,4,5‐isoxazoles starting from readily available 2,5‐dimethyl‐4‐nitroisoxazole (Scheme 20).^[^ ^103^ ^]^ This protocol combines a Knoevenagel reaction and Michael addition in one‐pot, with each step being catalyzed by different bases. In the first step, aldehyde **103** and isoxazole **104** were ground in a mortar in the presence of pyrrolidine as a catalyst, achieving high conversion to styryl isoxazoles **Int‐105** in 3–5 minutes, which could be isolated. Subsequently, a strong Michael donor **106** was added along with triethylamine to facilitate Michael addition and yield the desired isoxazole product **107** after further grinding within minutes. The products **107** were obtained as diastereomeric mixtures in a 1:1 ratio.

The scope of the presented method was investigated including various aryl aldehydes with EWGs and heteroaryl aldehydes. Also, ferrocene aldehyde afforded the corresponding products in good to excellent yields with ethyl nitroacetate (**106**, R^2^ = NO_2_, R^3^ = CO_2_Et). However, aryl aldehydes with EDGs did not result in complete conversion in the first step and required isolation by column chromatography before performing the Michael addition (examples excluded from Scheme 20). The scope of activated methylene compound **106** was also explored, but only substrates with at least one nitro or cyanide substituent acted as sufficiently strong Michael donors. For instance, dialkyl malonates did not react under these conditions, even with prolonged reaction times, limiting the generality of the protocol.

Equivalent solution‐based protocols also allow to perform this sequence in one‐pot with piperine as a catalyst, catalyzing both steps. However, these reactions require ethanol as a solvent, reflux conditions, and take up to 6 hours.^[^ ^104^, ^105^ ^]^ This clearly demonstrates the advantage of the MC protocol, despite its restriction to strong Michael donors under the reported conditions.

## 3 Sequences Including Cross‐Coupling Steps

In recent years, mechanochemistry has emerged as a powerful tool for performing cross‐coupling reactions under solvent‐free conditions, with protocols established for transformations such as Negishi, Suzuki‐Miyaura, Mizoroki‐Heck, Buchwald‐Hartwig, and Sonogashira couplings.^[^ ^106^, ^107^ ^]^ More recently, novel mechanochemical strategies have been developed that integrate cross‐coupling steps with other transformations or the in situ synthesis of reactive precursors into efficient one‐pot processes.

**Scheme 21:**
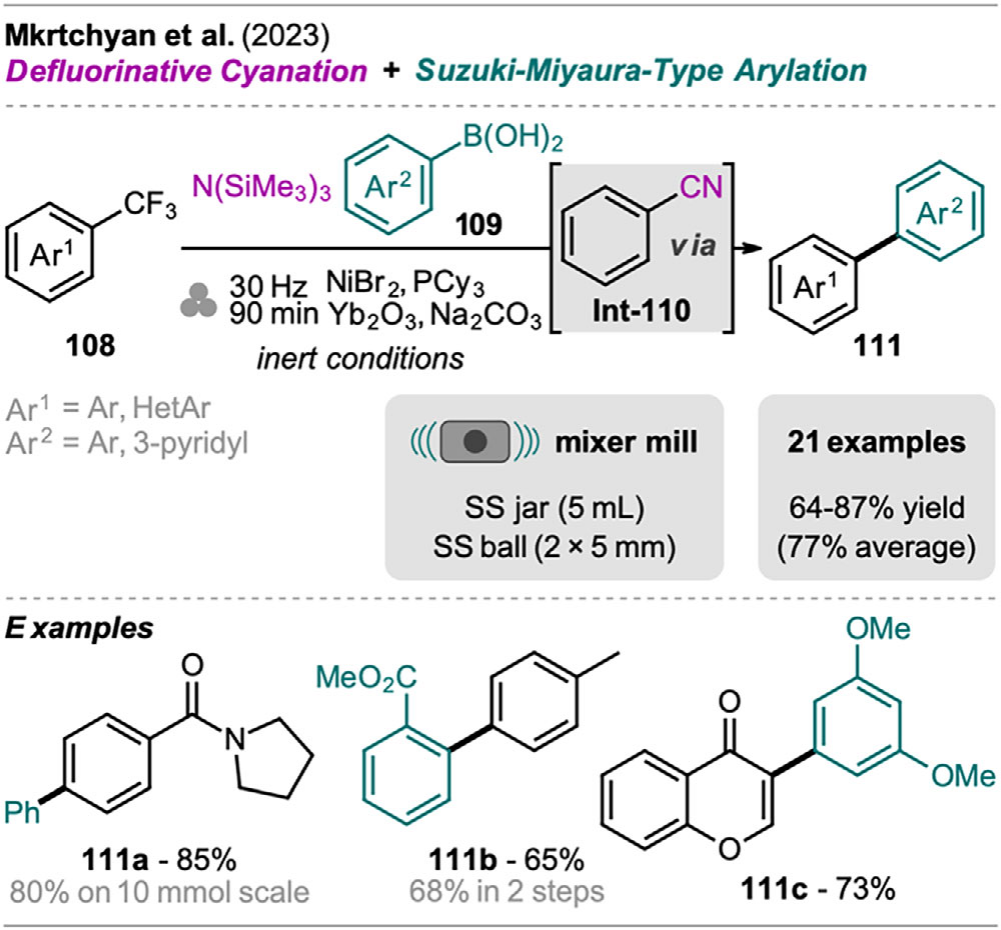
One‐pot domino‐type defluorinative cyanation of trifluoromethyl aryls and subsequent Suzuki‐Miyaura‐type arylation with boronic acids for the synthesis of biaryls.

An interesting combination of reactions, without prior evidence for mechanochemical protocols for both steps as single transformations, was reported by Mkrtchyan et al. (Scheme 21).^[^ ^108^ ^]^ The authors successfully performed the defluorinative cyanation of trifluoromethyl (hetero)arenes **108** followed by Suzuki‐Miyaura‐type cross‐coupling of the formed (hetero)arylnitriles **Int‐110** with boronic acids **109** in one‐pot to obtain biarenes **111**. Remarkably, the two transformations were compatible without requiring sequential addition of reagents. For the individual reactions, the defluorinative cyanation of **108** to give **Int‐110** was carried out using tris(trimethylsilyl)amine (N(SiMe_3_)_3_), ytterbium oxide, and NaHCO_3_. Yb_2_O_3_ is known for its high affinity to fluoride in C(sp^3^)‐F bonds, polarizing this bond and activating the CF_3_ moiety for coupling with nucleophiles like amines, which has also been demonstrated by the same group in the mechanochemical transformation of trifluoromethyl aryls into amides and Schiff bases.^[^ ^109^ ^]^ Meanwhile, the cross‐coupling of arylnitrile **Int‐110** with boronic acid **109** to give biarenes **111** was achieved under Ni‐catalysis, using NiBr_2_ and PCy_3_ as the catalytic system and Na_2_CO_3_ as the base. These optimized conditions were combined, allowing all starting materials and reagents (except NaHCO_3_) to be added in one jar in a glovebox and milled for 90 minutes. Noteworthy, while the cyanation step could be performed in air, the cross coupling required inert conditions.

To gain insights on the mechanism of the defluorinative cyanation, the authors performed DFT calculations for the formation of benzonitrile from trifluoromethylbenzene. Involving neutral state and radical anion calculations, the radical anion pathway was found to be energetically more favorable due to low activation barriers, and all activation barriers were considered as accessible under mechanochemical reaction conditions.

The applicability of the protocol was demonstrated with a broad substrate scope, including various trifluoromethylarenes bearing halo, carbonyl, and amine functionalities, giving the corresponding products in good to high yields. Additionally, heteroarenes were successfully transformed, with yields exceeding 67%. In terms of boronic acids, phenylboronic acid as well as functionalized aryl‐, even with fluoro‐substituents, and pyridinylboronic acids were well‐tolerated, too. The reaction could also be scaled up (10‐fold, 10 mmol) without significant decrease in yield.

While the authors do not provide green metrics for the mechanochemical protocol, the comparison to existing solution‐based methods for decyanative C─C coupling offers valuable insight into its environmental and operational benefits. For instance, Yu et al. reported a Ni‐catalyzed Suzuki‐Miyaura coupling of aryl nitriles in dioxane at 110 °C with reaction times of 20 hours.^[^ ^110^ ^]^ Similarly, Rahimi et al. demonstrated this transformation using a newly designed ligand and boronic esters in toluene at 100 °C for over 15 hours.^[^ ^111^ ^]^ Both methods involve significant solvent use, longer reaction times, and higher energy consumption compared to the mechanochemical approach reported by Mkrtchyan et al., which operates solvent‐free in a mixer mill within 90 minutes. This highlights clear advantages in terms of sustainability, supported by broad substrate tolerance and scalability.

**Scheme 22:**
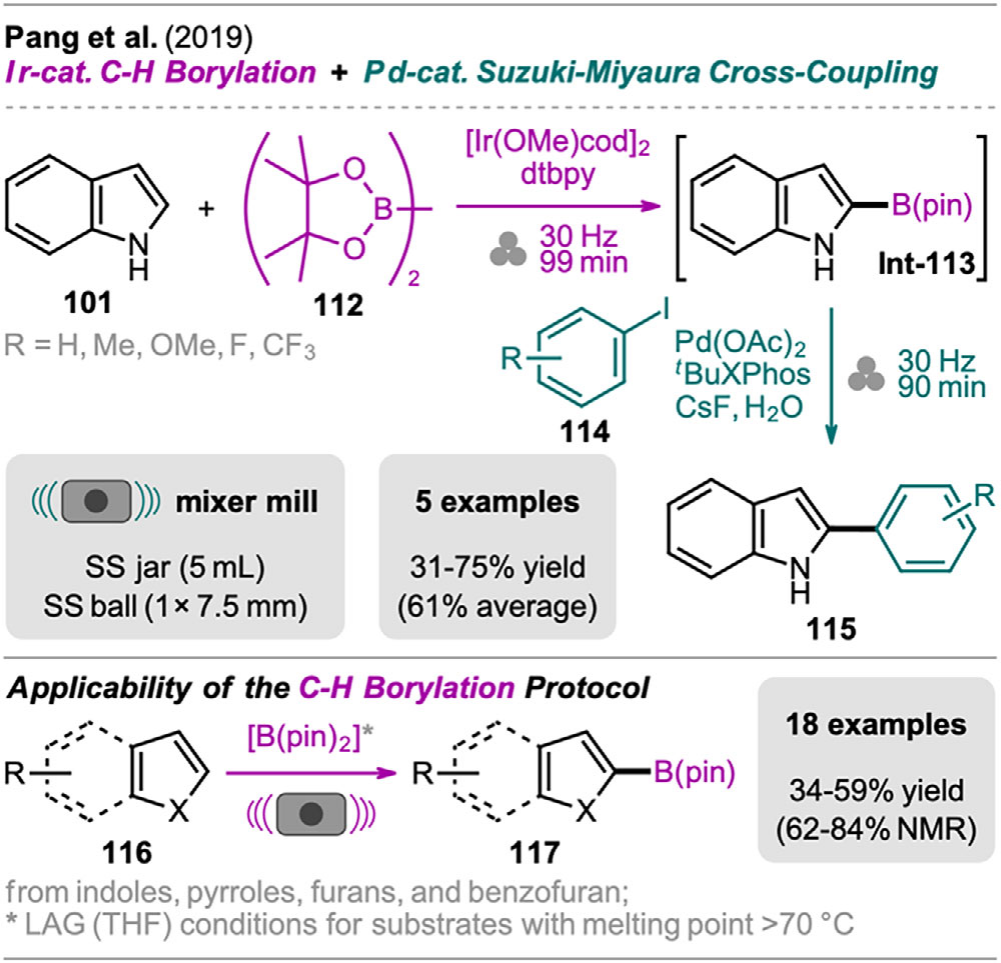
Sequential one‐pot C‐H borylation of indole and Suzuki‐Miyaura cross‐coupling to aryl iodides for the synthesis of 2‐aryl‐substituted indoles.

A classical Suzuki‐Miyaura cross‐coupling of aryl iodides with heteroaryl boronic esters under mechanochemical conditions has been demonstrated by Pang et al. (Scheme 22, top).^[^ ^112^ ^]^ While several protocols for mechanochemical Pd‐catalyzed C─C couplings of this type have been reported,^[^ ^107^, ^113^, ^114^, ^115^ ^]^ this protocol involves the prior synthesis of the boronic acid ester species **Int‐113** via C─H borylation in one‐pot. Notably, the mechanochemical iridium(I)‐catalyzed C─H borylation represents a novel development, as such transformation has not been previously reported in the literature via mechanochemistry. The C─H borylation of indole **101** was performed with bis(pinacolato)diboron (**112**) using iridium(I)/2,2′‐bipyridine complex as catalyst, in situ formed from [Ir(cod)OMe]_2_ and 4,4′‐di‐*tert*‐butyl‐2,2′‐dipyridyl (dtbpy). The reaction conditions were based on a solution protocol,^[^ ^116^ ^]^ but the mechanochemical transformation offered the significant advantage of being performed without the exclusion of air and moisture. A key factor for the successful outcome of this reaction was the choice of jar and milling ball size. While the reaction completely failed when performed in a 1.5 mL stainless steel (SS) jar with one SS ball (⌀ = 3 mm), switching to a 5 mL jar with one ball of 7.5 mm diameter resulted in high conversion toward boronic acid ester **Int‐113** (74% for model reaction with indole **101**). From a green chemistry perspective, it is noteworthy that both boryl groups of the diboron **112** were effectively utilized and only 0.6 equivalents were sufficient to achieve high yields for **Int‐113**, thereby minimizing waste. By subsequent addition of an aryl iodide **114** together with catalytic amounts of palladium(II) acetate and *tert*‐butyl‐XPhos, as well as stoichiometric amounts of cesium fluoride and water, further milling afforded the C‐C cross‐coupling product **115** in moderate to good yields (31–75%). While the reaction sequence was only explored starting from indole, the tolerance for EDGs and EWGs on the aryl iodide was investigated, resulting in diminished yields for methoxy‐ and trifluoromethyl‐substitution, showing some limitations in the applicability of this protocol.

Due to its novelty, the C─H borylation was explored including indoles bearing EWGs or EDGs at different positions, and furthermore, 2‐substituted pyrroles and furans **116** were successfully converted to the boronic acid esters **117** under the reported conditions (Scheme 22, bottom). However, for solid substrates with melting points >70°C the addition of THF as a LAG additive was necessary to achieve the formation of product **117** at all. Generally, isolated yields were lower than the corresponding NMR yields, explainable by losses during purification by CC due to partial hydrolysis and protonation.

Noteworthy, the authors conducted experiments on two substrates — solid indole (**101**) and liquid benzofuran (**116**, X = O, R = H) — to investigate the kinetics of the mechanochemical C‐H borylation reaction in comparison to the corresponding transformations in THF solution under inert conditions. In the mechanochemical approach, both substrates exhibited sigmoidal reaction kinetics with nonreactive periods of 50–60 minutes followed by a rapid increase in reaction rates. For indole, this transition coincided with a change in the rheology of the reaction mixture which likely improved mixing efficiency and thus increased the reaction rate. However, no such rheology change was observed for benzofuran. Based on these observations, the sigmoidal behavior was attributed to an induction period to generate the catalytically active species under MC conditions. Additional experiments including the preformation of the catalytically active species and subsequent addition of the substrate could have provided further insight into this hypothesis but were not included. In contrast, the solution‐phase reactions showed different behavior: for indole, an initially high reaction rate was observed which decreased after 20 minutes, whereas for benzofuran an extended nonreactive period of even 90 minutes was found.

Similar to the mechanochemical protocol, Robbins and Hartwig reported a sequential one‐pot C─H borylation and Suzuki‐Miyaura coupling in solution.^[^ ^117^ ^]^ Comparison of these two methods highlights the advantages of the mechanochemical protocol: (1) as it is typical for MC protocols, no solvent (or only a minute amount) is required, therefore reducing waste; (2) the reaction time was drastically shortened to approx. 3 hours, compared to 36 hours (18 hours for each step) in the SC protocol; (3) no external heating source was needed, making the process more energy‐efficient; and (4) the reaction was conducted without precautions for the exclusion of air and moisture, simplifying the experimental set‐up and reducing operational complexity compared to the solution‐based protocol. However, higher loadings of Ir catalyst were required in the MC approach (0.75 mol%) compared to the solution protocol (0.25 mol%).

## 4 Functional Group Transformations

### 4.1 Amide and Sulfonamide Bond Formations

**Scheme 23:**
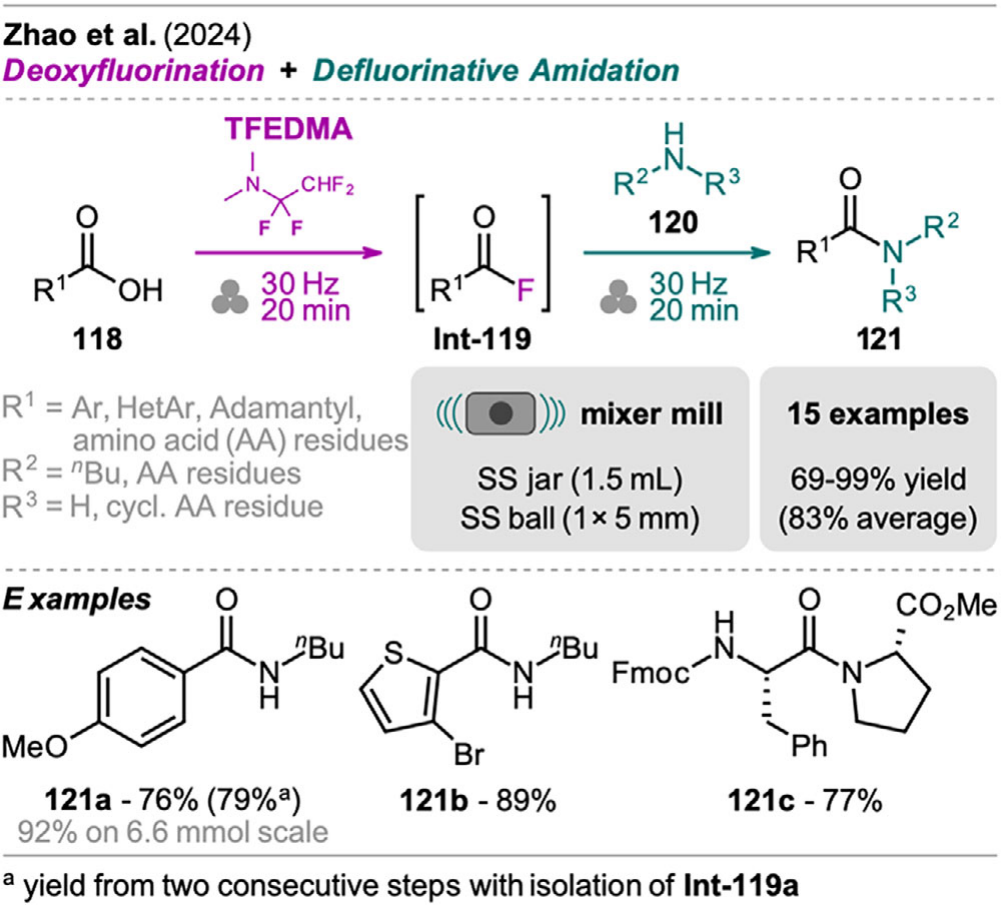
Synthesis of amides via a fluorination and defluorinative amidation sequence.

In 2024, Zhao et al. developed a mechanochemical protocol for the synthesis of amides through sequential one‐pot deoxyfluorination of carboxylic acids and coupling with primary and secondary amines (Scheme 23).^[^ ^118^ ^]^ The deoxyfluorination of carboxylic acid **118** was accomplished with 1,1,2,2‐tetrafluoroethyl‐*N*,*N*‐dimethylamine (TFEDMA), a thermally stable and industrially scalable reagent, giving the acylfluoride intermediate **Int‐119** within 20 minutes of milling. Subsequently, amine **120** was added, yielding the amide product **121** after a second milling step without the requirement of any additives. The entire sequence was carried out in air, allowing a simple reaction set‐up.

The generality of the protocol was demonstrated with a variety of benzoic acids bearing substituents (e.g., OMe, Ph, Br) at different positions, providing the corresponding amides **121** with 1‐butylamine in good yields ranging from 69–86%. The method also tolerated 3‐bromothiophene‐2‐carboxylic acid as a substrate, an example of heteroaryl carboxylic acids, giving product **121b** with high efficiency. Notably, the protocol was extended to amino acids for the synthesis of dipeptides starting from *N*‐Fmoc‐ or *N*‐Cbz‐protected amino acids, which were fluorinated and subsequently coupled with amino acid methyl esters (e.g., compound **121c**). The dipeptides were obtained in high yields of up to 99%, while no epimerization was observed (>99:1 *er* for all examples). Whereas the purification of amides and dipeptides **121** on a small scale was performed by CC, a scale‐up experiment with 4‐methoxybenzoic acid (33‐fold, 6.6 mmol) and 1‐butylamine demonstrated that recrystallization could be used as an alternative, less solvent‐intensive purification method, yielding the amide product **121a** with an excellent yield of 92% (76% on a 0.2 mmol scale).

In addition, the authors performed the two reactions separately and presented a substrate library of acyl fluorides **Int‐119**, including 20 examples, as well as additional amides **121**, synthesized from isolated alkyl or aryl acylfluorides and aniline derivatives.

The authors evaluated the presented method for the synthesis of dipeptides regarding greenness by calculating several green metrics including AE, CE, RME, and E‐factor, and compared these values for the same compound to solution‐based methods via acyl fluorides from Bolduc et al.^[^ ^119^ ^]^ and Lee et al.^[^ ^120^ ^]^ While some green metrics were more favorable for the SC protocols, the MC method showed significantly lower E‐factor values of 2.2 or 2.6 (depending on the purification method which affected the yield), without consideration of solvents used in the isolation and purification (SC: 52.2, 114.3). Furthermore, the protocol was compared to a mechanochemical approach by Dalidovich et al.^[^ ^121^ ^]^ which utilized an uronium‐based coupling reagent for the direct synthesis of amides from acids and amines. For the same dipeptide, Zhao et al.’s protocol demonstrated advantages in AE, RME, and E‐factor. However, both methods exhibit similarly strong performance across the calculated green metrics.

**Scheme 24:**
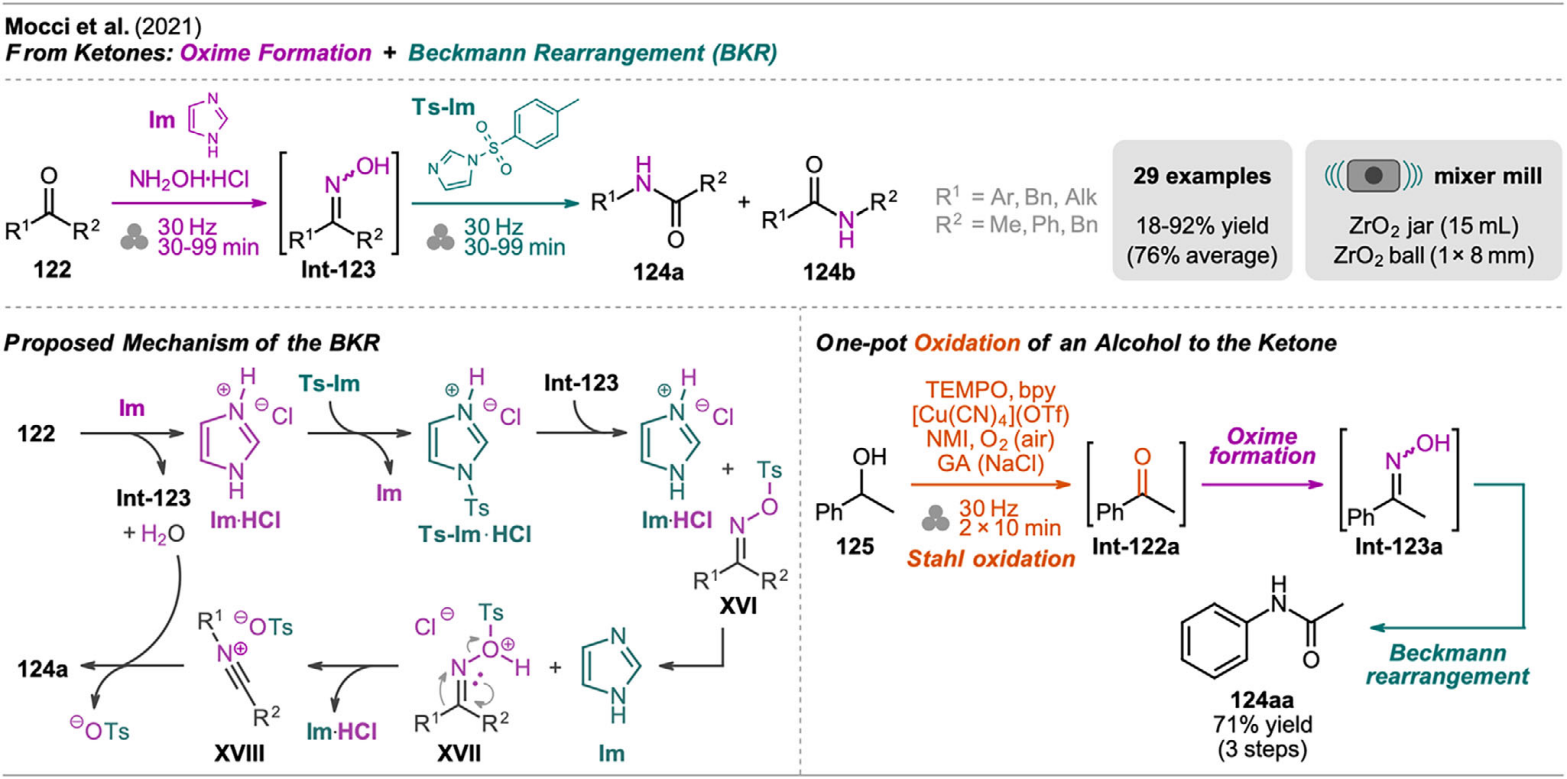
Sequential one‐pot transformation of ketones via oxime intermediates into amides by sequential Beckmann rearrangement including the one‐pot three‐step synthesis of acetophenone from 2‐phenylethanol.

Another mechanochemical procedure for the synthesis of amide frameworks was reported by Mocci et al., namely a one‐pot approach from ketones via oxime intermediates followed by Beckmann rearrangement (BKR) (Scheme 24).^[^ ^122^ ^]^ The BKR is an important reaction for the synthesis of secondary amides and especially significant for the industrial production of paracetamol (Hoechst‐Celanese process)^[^ ^123^ ^]^ and ε‐caprolactam, an intermediate in the synthesis of nylon‐6,6.^[^ ^124^ ^]^ However, conventional BKR methods often require prior synthesis and isolation of the oxime, strong acids, high temperatures, and solvents, and also catalytic versions starting from carbonyls rely on external heating.^[^ ^125^, ^126^, ^127^ ^]^

Mocci et al. achieved the formation of oxime **Int‐123** from ketones **122** using solid hydroxylamine hydrochloride and stoichiometric amount of imidazole, acting as a base and GA, resulting in complete conversion of **122** within 30–99 minutes of milling (Scheme 24). Previous investigations on the BKR of isolated oxime revealed that the BKR is successful in the presence of *p*‐tosylimidazole (Ts‐Im) and oxalic acid. Performing the two steps in one‐pot, no acid additive was required since imidazole hydrochloride (Im·HCl), generated in the formation of ketoximes, was sufficient to trigger the BKR. It is assumed that a proton transfer from Im·HCl to Ts‐Im promotes the formation of the tosyl ester on the oxime giving intermediate **XVI**. This then undergoes BKR upon protonation (oxonium cation **XVII**) resulting in nitrilium ion **XXIII** which upon hydrolysis gives the desired product **124a**. The mechanism displayed in Scheme 24 has been concluded by us based on the mechanism for the BKR on isolated oxime promoted by Ts‐Im and oxalic acid proposed by Mocci et al. The authors do not provide a detailed mechanism or mechanistic studies on the one‐pot two‐step reaction that is triggered by Im·HCl and does not require oxalic acid.

From acetophenone derivatives (**122**, R^2^ = Me) with various substituents on the phenyl ring and symmetrical ketones (**122**, R^1^ = R^2^), amides **124a** were obtained in good to excellent yields, except for 4′‐nitroacetophenone, which gave the corresponding amide only in 18% yield. Notably, paracetamol was prepared in 84% yield from 4′‐hydroxyacetophenone. Also, cyclic ketones reacted smoothly to the corresponding lactams in good yields, overcoming susceptible ring strain. From other ketones, mixtures of the two regioisomers **124a** and **124b** were obtained, but also in moderate to good yield. Regioselectivity was shown to be strongly dependent on the residues.

Notably, most amides could be isolated and purified by aqueous washing steps or extraction and subsequent filtration over a short pad of silica, not requiring CC. The reaction could also be scaled up (8‐fold, 8 mmol) without any loss of reaction efficiency.

Taking a step further, the authors demonstrated that the sequence toward amides can also be started from the corresponding secondary alcohols, which was exemplified in the synthesis of amide **124aa** from alcohol **125**. Mechanochemical Stahl oxidation conditions, adapted from a previous report by the same group,^[^ ^128^ ^]^ were found to be compatible with the following oxime formation‐BKR sequence, allowing all three steps sequentially in one‐pot (Scheme 24). Following this route, amide **124aa** was obtained in 71% from alcohol **125** over three steps.

For the synthesis of amide **124aa**, the authors evaluated the greenness of the method by calculating various green metrics. The MC protocol demonstrated a remarkably low E‐factor of 3.1 (100.6 including purification) and a high EcoScore of 73, in contrast to a similar process in solution^[^ ^129^ ^]^ with an E‐factor of 242.8 (solvent and silica for CC not included) and an EcoScale of 36. These metrics underscore the environmental benefits of the MC approach, which arise from several key advantages: (1) the one‐pot combination of oxime formation and BRK, (2) no need for external heating, and (3) the avoidance of column chromatography for most examples.

In conclusion, this method can be emphasized as an eco‐efficient process, offering solvent‐free and mild conditions, and the use of inexpensive and eco‐friendly reagents.

**Scheme 25:**
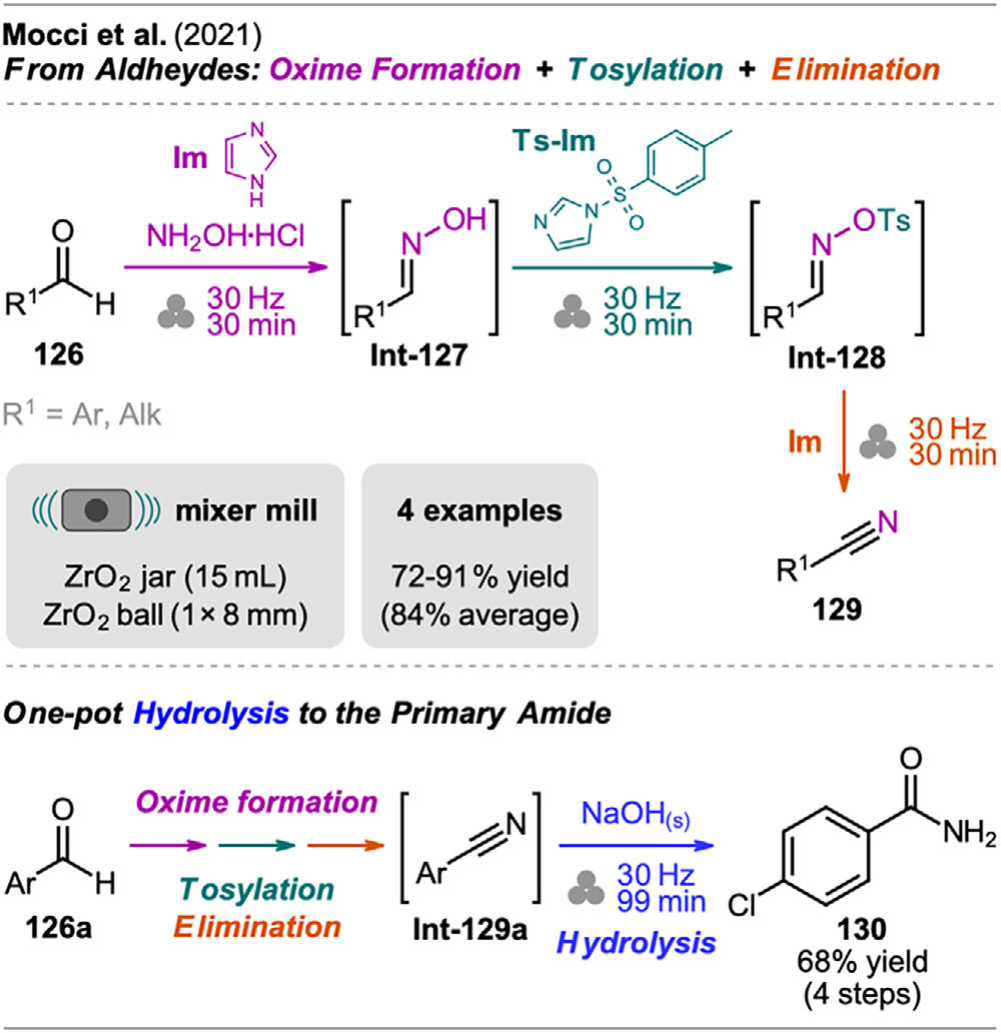
Sequential one‐pot transformation of aldehydes via oxime intermediates into nitriles by sequential tosylation and elimination including an example for the sequential one‐pot hydrolysis of the nitrile to the primary amide.

Following this strategy, Mocci et al. further investigated an array of aldehydes **126** to obtain nitriles **129** (Scheme 25).^[^ ^122^ ^]^ By the addition of Ts‐Im, the aldoxime **Int‐127** was transformed into the *O*‐tosyl‐oxime **Int‐128**. This intermediate underwent subsequent elimination, which was significantly sped up by the addition of further imidazole, giving nitrile **129** in good to excellent yield.

In addition, sequential one‐pot hydrolysis of nitrile **Int‐129a** could be achieved by addition of a milling step with solid sodium hydroxide to the sequence, however, with quite long reaction time of 99 minutes (Scheme 25). The primary amide **130** was obtained in 68% over 4 steps.

**Scheme 26:**
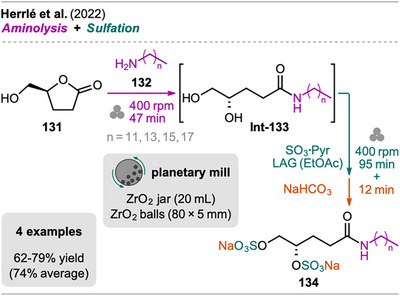
Synthesis of potential anionic surfactants via an aminolysis and sulfation sequence from a γ‐butyrolactone derivative.

Herrlé et al. presented a protocol for the aminolysis of (*S*)‐γ‐hydroxymethyl‐γ‐butyrolactone (2H‐HBO) with primary alkyl amines (C12‐C18) and subsequent sulfation in one‐pot to obtain bis(sulfooxypentyl)amides as potential anionic surfactants, with the mechanochemical sulfation being unprecedented in literature to date (Scheme 26).^[^ ^130^ ^]^ The amide formation was achieved by cyclic milling of equimolar amounts of lactone **131** and amine **132** in a planetary mill, without the need for addi base. In one‐pot, sulfur trioxide pyridine complex (SO_3_⋅pyr) along with ethyl acetate as a LAG additive was added and further milling resulted in the mechano‐sulfation of the amide intermediate **Int‐133**, which was then further milled with NaHCO_3_ to obtain product **134** in its sodium salt form. In total four bis(sulfooxypentyl)amides **134** with alkyl chains of different lengths were synthesized in good yields.

Green metrics, including AE, RME, PMI, and E‐factor, were calculated, and comparison to a classical solvent‐based route in two steps was drawn. The mechanochemical method demonstrated excellent E‐factors (<2) for each example, whereas the SC method resulted in higher values of 7 and up to 25 for the aminolysis and sulfation, respectively. However, these calculations excluded the workup and purification steps. Additionally, the MC protocol exhibited a significantly better PMI of up to 38% in comparison to the SC method, which achieved only 14% for the aminolysis and 5% for the sulfation. Overall, this process can be considered superior to the solution process in terms of environmental sustainability, featuring shorter total reaction times of 2.5 hours and higher obtained yields.

**Scheme 27:**
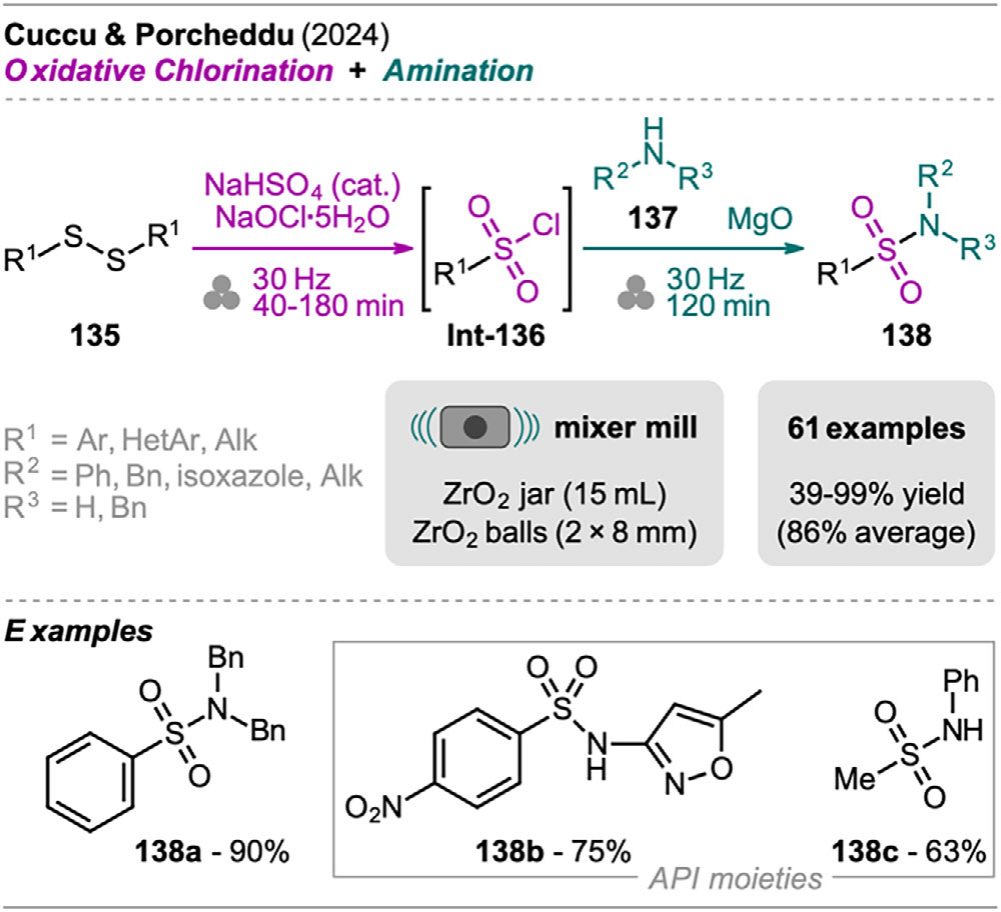
Synthesis of sulfonamides via sequential oxidative chlorination and amination of disulfides.

In 2024, Cuccu and Porcheddu reported the solvent‐ and metal‐free one‐pot two‐step synthesis of sulfonamides from dialkyl and diaryl disulfides (Scheme 27).^[^ ^131^ ^]^ In this protocol, the oxidative chlorination of disulfide **135**, avoiding odorous thiols, to sulfonyl chloride **Int‐136** and sequential amination are combined. Sodium hypochlorite (as pentahydrate) was found to be a suitable chlorinating agent to obtain the sulfonyl chloride intermediate **Int‐136** with a high conversion rate in the presence of sodium bisulfate as an inorganic acid catalyst. The sulfonyl chlorides **Int‐136** could be isolated in good to excellent yield (no CC) or directly converted into sulfonamides **137** in one‐pot. The sequential amination was performed by addition of primary or secondary amine **137** along with an excess of MgO as base and further milling for 2 hours. Other solid inorganic bases were not suitable (e.g., K_2_CO_3_) and were found to promote hydrolysis of **Int‐136** due to their high hygroscopicity, which MgO does not exhibit.

The generality of the method was demonstrated through a broad substrate scope. Various diaryl disulfides with both EDGs and EWGs in *para* or *meta* position were well‐tolerated and resulted in excellent yields of **138** with different primary and secondary amines, for example, dibenzylamine, aniline, and piperidine. Notably, diisopropyl‐ and dimethyl‐disulfide also provided sulfonamides, the latter one yielding an API moiety found in the backbone of the anti‐inflammatory drug nimesulide (compound **138c**).^[^ ^123^ ^]^ The protocol was further extended to amino acid methyl esters, yielding highly functionalized sulfonamides. With the aim to access additional API moieties, isoxazole amines were investigated too (e.g., compound **138b**), but required LAG conditions (*N*‐methyl imidazole) for success in the amination step.

This method clearly stands out in comparison to solution‐based protocols, not only because of its one‐pot operation, but also by eliminating large solvent and acid amounts and replacing pyridine or triethylamine, often used as bases for the amination, by nontoxic MgO. The environmental advantages are underscored by comparison of green metrics, even though the authors only provide data for the two transformations in a consecutive fashion with isolation of the sulfonyl chloride. For a similar protocol in solution, the E‐factors — 208.9 and 111.8 for the chlorination and amination, respectively, — are significantly higher than for the MC protocol (chlorination: 15.9; amination: 86.8), including solvents for isolation and purification. Additionally, the mechanochemical amination protocol shows notable improvement of the RME with 54% for the MC protocol compared to 22% for the SC approach.

**Scheme 28:**
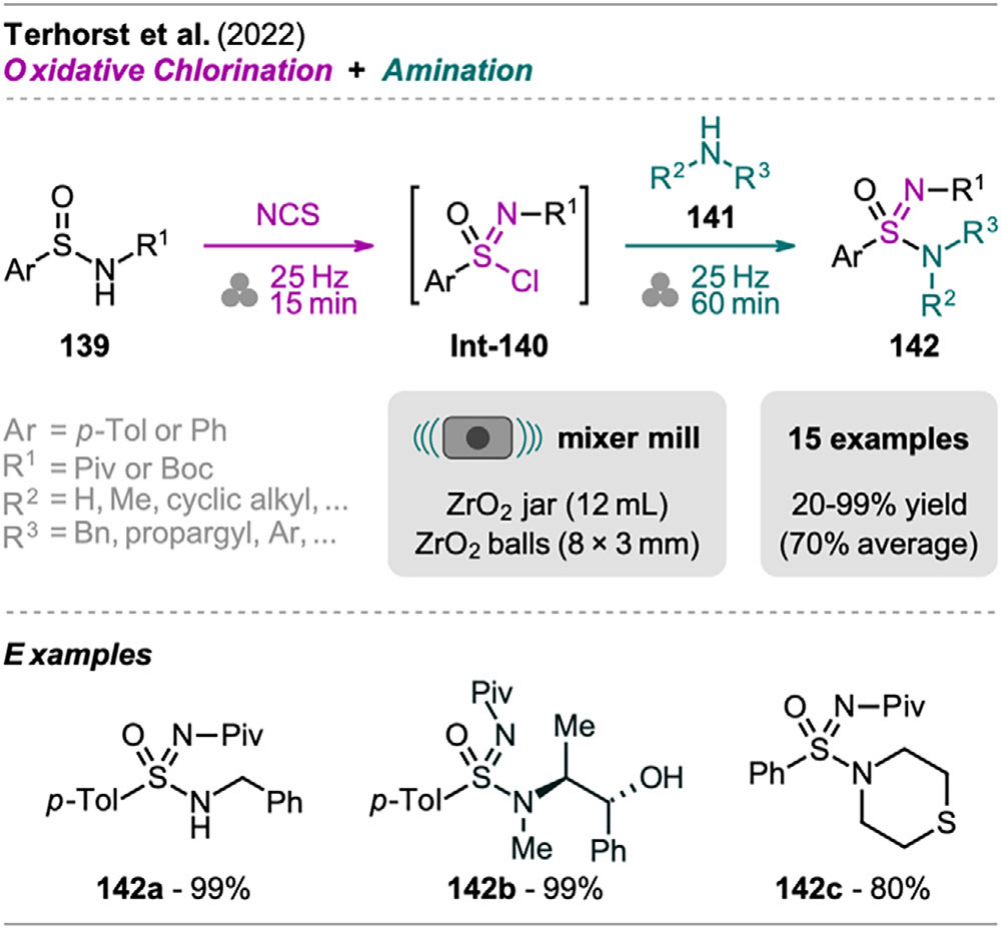
Synthesis of sulfonimidamides via sequential oxidative chlorination and amination of sulfinamides.

The sequence of oxidative chlorination and subsequent amination was also demonstrated for the synthesis of sulfonimidamides by Terhorst et al. (Scheme 28).^[^ ^132^ ^]^ In this protocol, reactive sulfonimidoyl chloride **Int‐140** was readily formed by milling of sulfinamide **139** with stoichiometric amount of *N*‐chlorosuccinimide (NCS), a strong chlorinating agent. Subsequently, a primary or secondary amine **141** was added, yielding the sulfonimidamide **142** without the presence of base.

The applicability of the protocol was demonstrated by investigating various amines with pivaloyl protected *p*‐toluenesulfinamides, including alkylic amines, cyclic secondary amines, anilines, etc., *N*H‐free sulfinamides were found to be unsuitable starting materials and only oligomeric products were observed.

A similar solution‐based method for the synthesis of sulfonimidamides from benzyl‐protected sulfinamides with chlorination and amination in one‐pot was reported by Jabczun et al.^[^ ^133^ ^]^ The reaction was carried out in acetonitrile at room temperature using trichloroisocyanuric acid as a chlorinating agent, followed by sequential amination with morpholine in the presence of triethylamine as a base. Comparing these two methods, the advantages but also disadvantages of the MC protocol become clear: (1) While NCS is commonly used as a chlorinating agent, it is thermally unstable with the risk of explosion at elevated temperatures. Although the MC reaction is performed at room temperature, mechanochemical processes often generate heat due to friction and impact during milling, which can result in a significant rise in temperature, depending on several milling parameters.^[^ ^134^ ^]^ This can lead to an increased risk for potential explosions, questioning the safety of NCS use in the ball mill. In contrast, TCCA is thermally stable and, furthermore, contains three chlorine atoms per molecule, allowing the use of sub‐stoichiometric amounts.^[^ ^135^ ^]^ (2) In the mechanochemical process, no base is required for the amination step, reducing the amount of waste generated from the process. (3) While the MC approach eliminates the need for solvents during the reaction itself, product purification still requires CC, as it is the case in the SC approach.

### 4.2 Fluorine‐Containing Functional Groups

**Scheme 29:**
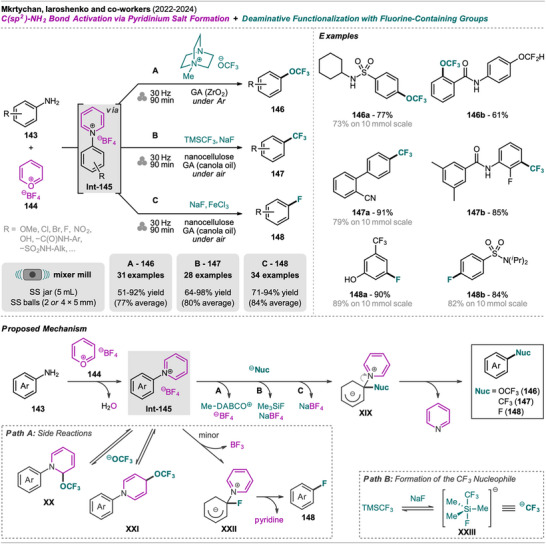
Deaminative functionalization of aryl amines with fluorine or fluorine‐containing groups via in situ generated pyridinium salt intermediates.

Mkrtchyan, Iaroshenko, and coworkers thoroughly investigated mechanochemical transformations for the synthesis of fluorine‐containing organic molecules, presenting three one‐pot methods based on a common pathway: the in situ activation of a C(sp^2^)‐NH_2_ bond followed by deaminative nucleophilic aromatic substitution (S_N_Ar).^[^ ^136^, ^137^, ^138^ ^]^ In their approach, aromatic anilines as cheap and easily available synthetic precursors were utilized to synthesize trifluoromethoxy (–OCF_3_), trifluoromethyl (–CF_3_), and fluoro (–F) substituted arenes, depending on the nucleophile used (Scheme 29A‐C).

The first protocol in this series, presented in 2022, focused on the synthesis of trifluoromethoxy aryls **146** and was the starting point for further investigations (Scheme 29A).^[^ ^136^ ^]^ The authors showed that the C(sp^2^)‐NH_2_ bond of anilines **143** can be activated with pyrylium reagent **144**, giving a pyridinium salt intermediate **Int‐145**, which readily underwent S_N_Ar. While the selective functionalization of amino(hetero)arenes by pyrylium salts is known in the literature,^[^ ^139^, ^140^, ^141^ ^]^ this marked the first application of such chemistry in a mechanochemical setting. Among various OCF_3_ sources tested, diazabicyclooctanium trifluoromethanolate was identified as the most effective, and under the optimized conditions with zirconium(IV) oxide as GA trifluoromethoxy arenes **146** were obtained in good to excellent yields.

The authors state that ZrO_2_ was used as grinding additive to improve mixing and prevent the formation of a gum or paste since some of the starting materials were liquids and furthermore water is produced from the condensation between amine **143** and pyrylium salt **144**. Additionally, DFT calculations were performed to elucidate the mechanistic route for the second step ‐ the substitution and dissociation of the pyridinium salt **Int‐145**. It was revealed that single electron transfer followed by dissociation into the phenyl radical and pyridine is thermodynamically disfavored. Therefore, attention was drawn to the mechanistic route via nucleophilic attack of the OCF_3_ anion at **Int‐45** to result in intermediate **XIX** and desired product **146** upon elimination of pyridine. Next to the desired substitution at the carbon bearing the pyridinium moiety it was proposed that metastable intermediates could be formed by *ortho*‐ and *para*‐substitution of a hydrogen at the pyridine ring (intermediates **XX** and **XXI**). However, for these intermediates relatively high energies and a very small energetic barrier of only 2 kcal/mol for disintegration were calculated. Therefore, the nucleophilic attack at the pyridinium ring was considered reversible and irrelevant to the performance of the reaction. Product **146** was found to be the thermodynamically most stable product with a splitting of ‐38.9 kcal/mol compared to the isolated reactants **Int‐145** and OCF_3_ anion.

In addition, another side reaction was observed for a few substrates ‐ the formation of the fluorinated product **148** via intermediate **XXII**. It is proposed that this concurrent reaction takes place with the BF_4_ anion as the fluoride nucleophile source. A control experiment with [1,1′‐biphenyl]‐4‐amine leaving out the OCF_3_ nucleophile source revealed that indeed BF_4_ anion can act as a fluoride source and after prolonged reaction time of 6 hours the fluorinated product **148** was obtained in 70% yield.

The scope of the method was investigated, revealing broad applicability. Various anilines with EWGs and EDGs at different positions on the aromatic ring were well‐tolerated, although slightly lower yields were observed for *ortho*‐substituted substrates, probably due to steric hindrance for the nucleophilic attack of the OCF_3_ anion. Beyond anilines, amides and sulfonamides containing amino groups on a benzene ring next to diverse other functional groups showed compatibility (e.g., compounds **146a** and **146b**), highlighting the applicability of this method for late‐stage functionalization. Additionally, the scalability of the protocol was demonstrated for 5 examples (10‐fold, 10 mmol), with no significant decrease in yields.

Building on this strategy, a method for the trifluoromethylation of aminoarenes **143** to obtain trifluoromethyl (hetero)arenes **147** was presented in 2024 (Scheme 29B).^[^ ^137^ ^]^ The optimized conditions involved trimethyl(trifluoromethyl)silane as CF_3_ source, sodium fluoride as an activator for TMSCF_3_, nanocellulose as a solid reaction medium, and commercial canola oil as a lubricant. Interestingly, the reaction did not proceed in the absence of nanocellulose, prompting the investigation of its role in the reaction mechanism. First, nanocellulose appeared to act as a scavenger for water generated during the condensation reaction between amine **144** and pyrylium salt **144**. Second, mechanistic DFT studies suggested that nanocellulose forms a complex with **Int‐145** via hydrogen bonding, effectively catalyzing the conversion into the trifluoromethylated product **147**. As for the substitution with OCF_3_, DFT calculations again suggested that the CF_3_ nucleophile from **XXIII** is introduced via aromatic nucleophilic substitution to deliver the desired trifluoromethyl arene **147**. Noteworthy, the reaction was also successful when toluene was used as a LAG additive instead of canola oil. However, canola oil represents a cheaper, nonhazardous, and biodegradable alternative compared to organic solvents.

The scope of the method was demonstrated using a variety of aryl and heteroaryl amines bearing EDGs and EWGs (e.g., compound **147a**), as well as amides and sulfonamides, all of which gave the corresponding trifluoromethyl products in good to excellent yields. Furthermore, the method was also used for the gram‐scale synthesis (10‐fold, 10 mmol) of three representative examples, giving the products in acceptable yields.

Further expanding the strategy, Mkrtchyan, Iaroshenko, and coworkers demonstrated the deaminative fluorination of (hetero)aryl amines **143** via in situ activation as pyridinium salts **Int‐145** (Scheme 29C).^[^ ^138^ ^]^ Their investigations were prompted by earlier observations during the trifluoromethoxylation study (Scheme 29A) where control experiments revealed that the BF_4_ counter anion can act as an F source, however, not very efficiently. Subsequent investigations revealed that the deaminative fluorination of **143** via **Int‐145** could be achieved with NaF as the fluorine source, iron(III) trichloride as a catalyst, nanocellulose as a solid reaction medium, and canola oil as a lubricant (Scheme 29C). Similar to the trifluoromethylation process, no reaction occurred in the absence of nanocellulose, and exclusion of either FeCl_3_ or canola oil resulted in significantly lower yield (<14%).

A plausible mechanism for the deaminative fluorination of arenes was proposed based on their previous studies on mechanochemical deaminative functionalization. However, the exact role of FeCl_3_ acting as a catalyst remained unclear. Furthermore, the role of nanocellulose in this transformation was not further elucidated and described to act as a scavenger for H_2_O formed in the condensation between amine **143** and pyrylium salt **144** to give **Int‐145**.

As for the earlier methods, the fluorination protocol was found to be widely applicable and a variety of (hetero)aryl amines gave the corresponding fluorinated product in good to excellent yields, showing compatibility with diverse functional groups (e.g., halo, ester, or cyano, compound **148a**). Furthermore, substrates with amide as well as sulphonamide connections (e.g., compound **148b**) were well‐tolerated, and the method was employed for gram‐scale synthesis (10‐fold, 10 mmol), without significant decrease in yields.

**Scheme 30:**
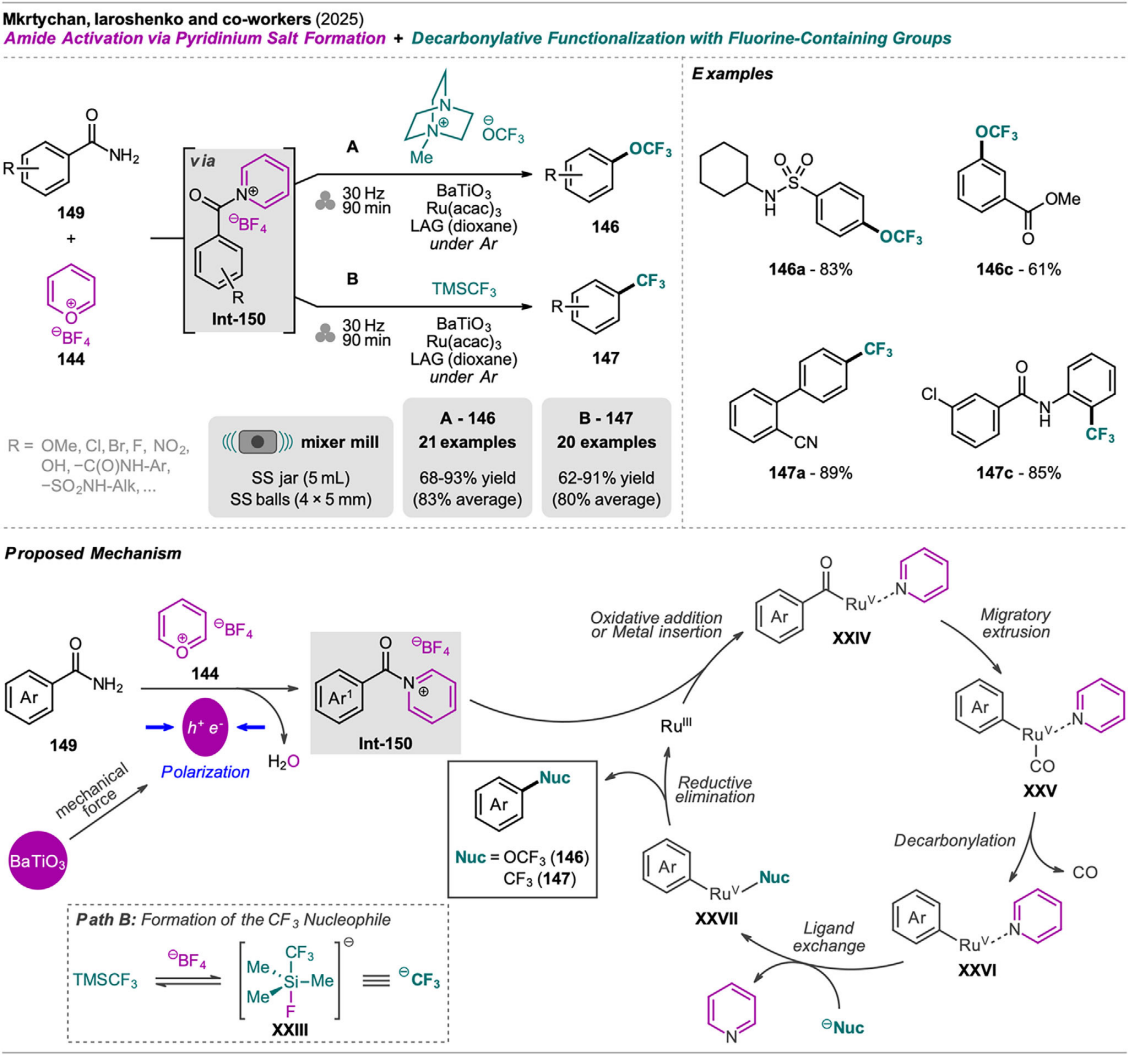
Decarbonylative functionalization of aryl amides with fluorine‐containing groups via in situ generated pyridinium salt intermediates.

Inspired by the work of Cornella,^[^ ^142^, ^143^ ^]^ who demonstrated the functionalization of sulfonamides in solution using pyrylium salt **144** as an activator, Mkrtchyan, Iaroshenko, and coworkers extended their investigations and presented the decarbonylative trifluoromethoxylation (Scheme 30A) and trifluoromethylation (Scheme 30B) of amides **149** activated by in situ formation of the pyrdinium salt **Int‐150**.^[^ ^144^ ^]^ For both transformations, ruthenium catalysts were found to successfully catalyze the deamination and subsequent coupling with either the OCF_3_ or CF_3_ anion, yielding products **147** or **148**, respectively. Ruthenium(III) acetylacetone was identified as the optimal catalyst. However, the reaction only resulted in high yields when an excess of piezoelectric barium titanate (BaTiO_3_) was added to the reaction mixture.

Considering its role, the authors proposed that BaTiO_3_ undergoes polarization under mechanical stress, with the resulting energy input exciting intermediate **Int‐150** and facilitating its oxidative addition to the ruthenium catalyst. Despite this hypothesis, the exact mechanistic role of BaTiO_3_ in the system remained unresolved. Following the Ru^III^–Ru^V^ catalytic cycle, which was proposed based on literature and previous studies, Ru‐acyl intermediate **XXIV** undergoes migratory extrusion of CO giving intermediate **XXV** which upon decarbonylation generates Ru‐aryl intermediate **XXVI**. Next, the pyridine ligand is exchanged by a nucleophilic OCF_3_ or CF_3_ species, the latter coming from a pentacoordinated silicon intermediate species **XXIII** formed from TMSCF_3_ and BF_4_ anion, acting as a synthetic equivalent of the CF_3_ anion, as displayed in Scheme 30. Finally, intermediate **XXVII** undergoes reductive elimination to deliver the desired product **146** or **147** and regenerate the active Ru^III^ species.

For the trifluoromethoxylation (Scheme 30A), trifluoromethanolate served as OCF_3_ nucleophile source, consistent with its earlier use in the protocol starting from amines. In the case of trifluormethylation (Scheme 30B), trifluoromethyl trimethylsilane was used as CF_3_ source. Otherwise, the reaction conditions were identical, and in both cases 1,4‐dioxane was employed as a LAG additive, ensuring effective reaction conditions.

Independent of the introduced fluoro group, various aryl amides with similar aryl moieties used in the deaminative approaches (Scheme 29A,B) were well‐tolerated by the presented decarbonylative method, again including examples with amide and sulfonamide connections.

Interestingly, all methods for the introduction of fluorine or fluorine‐containing functional groups developed by Mkrytchan, Iaroshenko, and coworkers^[^ ^136^, ^137^, ^138^, ^144^ ^]^ were found to only work in the solid state, as attempts to perform the transformations in solution or under neat conditions either failed completely or resulted in significantly lower yields, even when performed at elevated temperatures and with prolonged reaction times. These findings strongly suggest that mechanical activation plays a crucial role in enabling the reactivity, pointing to a mechanistically distinct pathway that is uniquely accessible in the solid state.

### 4.3 Other Functional Group Transformations

**Scheme 31:**
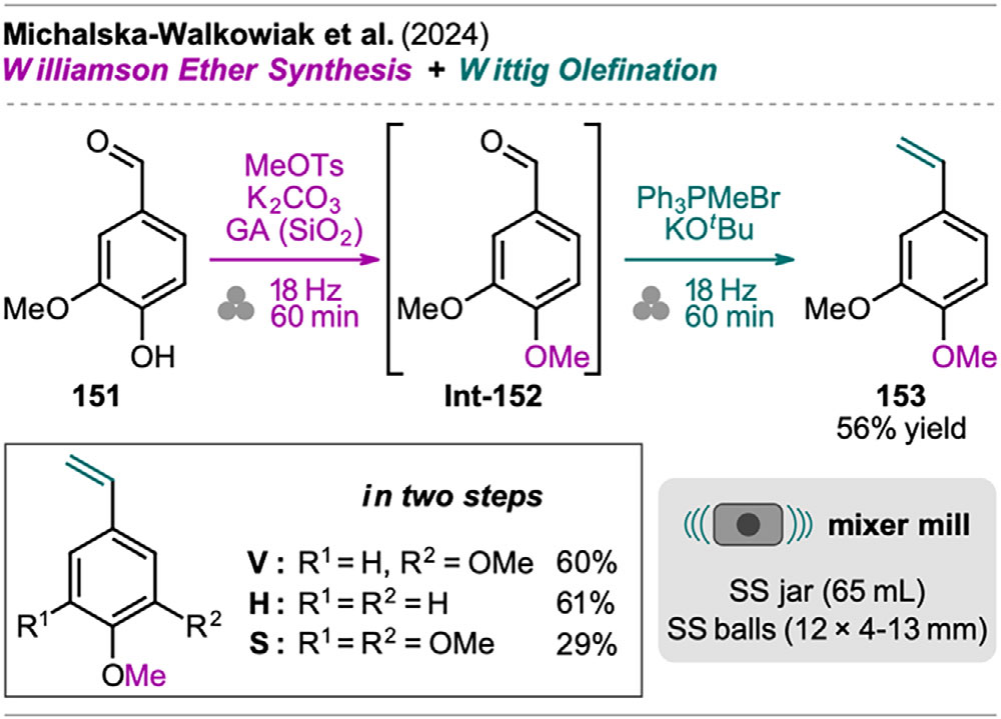
Sequential one‐pot Williamson ether synthesis and Wittig olefination for the synthesis of vinyldimethoxybenzene from vanillin.

Aiming for a greener synthesis of bio‐sourced styrene derivatives as monomers for bio‐based polymers, Michalska‐Walkowiak et al. presented a mechanochemical one‐pot Williamson ether synthesis and Wittig olefination sequence in 2024, albeit demonstrated only for a single example (Scheme 31).^[^ ^145^ ^]^ In their work, vinyldimethoxybenzene **153** was synthesized from vanillin (**V**, **151**) by milling with methyl tosylate (MeOTs), potassium carbonate as a base, and silica as GA to obtain the ether product **Int‐152**. Subsequently, methylphosphonium bromide (Ph_3_PMeBr) along with potassium *tert*‐butoxide was added, and further milling resulted in the olefinated product **153** in 56% isolated yield with a total reaction time of 2 hours. Notably, the active Wittig ylide was formed in situ and subsequently reacted with **Int‐152**.

The authors also performed the sequence with in‐between isolation of **Int‐152** in solution, which in general resulted in higher overall yields compared to the mechanochemical one‐pot protocol (SC: 74%). However, despite this disadvantage, the MC procedure offered various advantages over the solution‐based reactions. For the ether synthesis: (1) While the reaction in solution took 48 hours, the ball milling approach only required 1 hour of reaction time. (2) The reaction was performed at room temperature, compared to reflux conditions in solution. For the Wittig olefination, reaction time was again drastically shorter in the MC protocol (1 hour for MC vs. 24 hours for SC), along with various other advantages: (1) While the solution‐based approach requires exclusion from moisture and therefore operation under inert conditions, the mechanochemical Wittig olefination can be performed in air. (2) The simplicity of the mechanochemical protocol stands out, as it avoids the tedious step of ylide pre‐formation at low temperatures and temperature control in general. (3) Instead of strong bases like butyl lithium, which are highly reactive and require careful handling, the mechanochemical method employs a solid and easy‐to‐handle base, such as potassium tert‐butoxide, further enhancing its practicality and safety.

Additionally, 4‐hydroxybenzaldehyde (**H**) and syringaldehyde (**S**) were used as starting materials, however, the corresponding vinyl substrates were synthesized in two consecutive steps with isolation of the ether product. While the Wittig olefination gave good to excellent yields (77‐93%), rather low yields were obtained in the Williamson ether synthesis, especially for syringaldehyde (38%), resulting in low to moderate overall yields (Scheme 31).

**Scheme 32:**
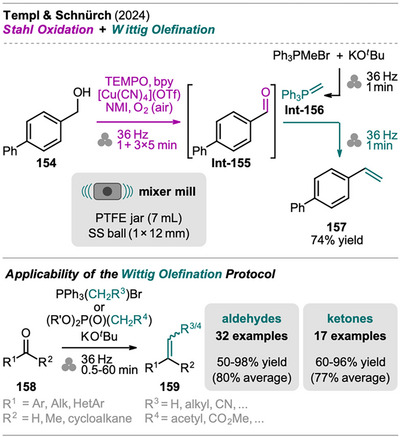
Sequential one‐pot Stahl oxidation and Wittig olefination of a primary alcohol to obtain the corresponding vinyl compound.

In the same year, Templ and Schnürch presented a highly efficient one‐pot two‐step mechanochemical Stahl oxidation and Wittig olefination sequence, although only on one example.^[^ ^146^ ^]^ By adapting previously reported mechanochemical Stahl oxidation conditions from Porcheddu et al.,^[^ ^128^ ^]^ alcohol **154** was oxidized to the corresponding aldehyde **Int‐155** and subsequently methylenated, yielding the desired product **157** in 74% yield (Scheme 32, top). The conditions of the mechanochemical Wittig reaction, which included the preformation of the Wittig ylide **Int‐156** by milling of methylphosphonium bromide with potassium *tert*‐butoxide and addition of the obtained paste to the oxidized intermediate **Int‐155**, were thoroughly investigated by the authors prior to the demonstration of this sequence and closely resemble the protocol developed by Michalska‐Walkowiak et al.,^[^ ^145^ ^]^ although both studies were conducted independently. Taking a closer look at the mechanochemical Wittig olefination, Templ and Schnürch adapted and optimized an earlier protocol by Balema and Pecharsky (2002),^[^ ^147^ ^]^ achieving a simplified and robust method. As mentioned earlier, also in this protocol the need for air and moisture exclusion could be eliminated and the solid, easy‐to‐handle base potassium *tert*‐butoxide was used. Most examples required only 30 seconds to 5 minutes of milling and allowed the one‐pot combination of substrate **159**, Wittig salt, and base. The protocol was demonstrated as a versatile approach and found to be compatible with various aldehydes and ketones, but also phosphonium halides and phosphonates, providing diverse olefins **160** with good to excellent yields (Scheme 32, bottom).

Remarkably, the reaction times could be shortened drastically compared to the Wittig olefination protocol presented by Michalska‐Walkowiak et al.,^[^ ^145^ ^]^ as a result of fine‐tuning of the reaction parameters and equivalents of reactants. The further development of a sequential one‐pot oxidation and Wittig olefination expands the scope and utility of this approach. The compatibility of the two methods reduces the need for intermediate workup or purification steps, streamlining the synthesis and further emphasizing the environmental and practical benefits of the mechanochemical approach.

**Scheme 33:**
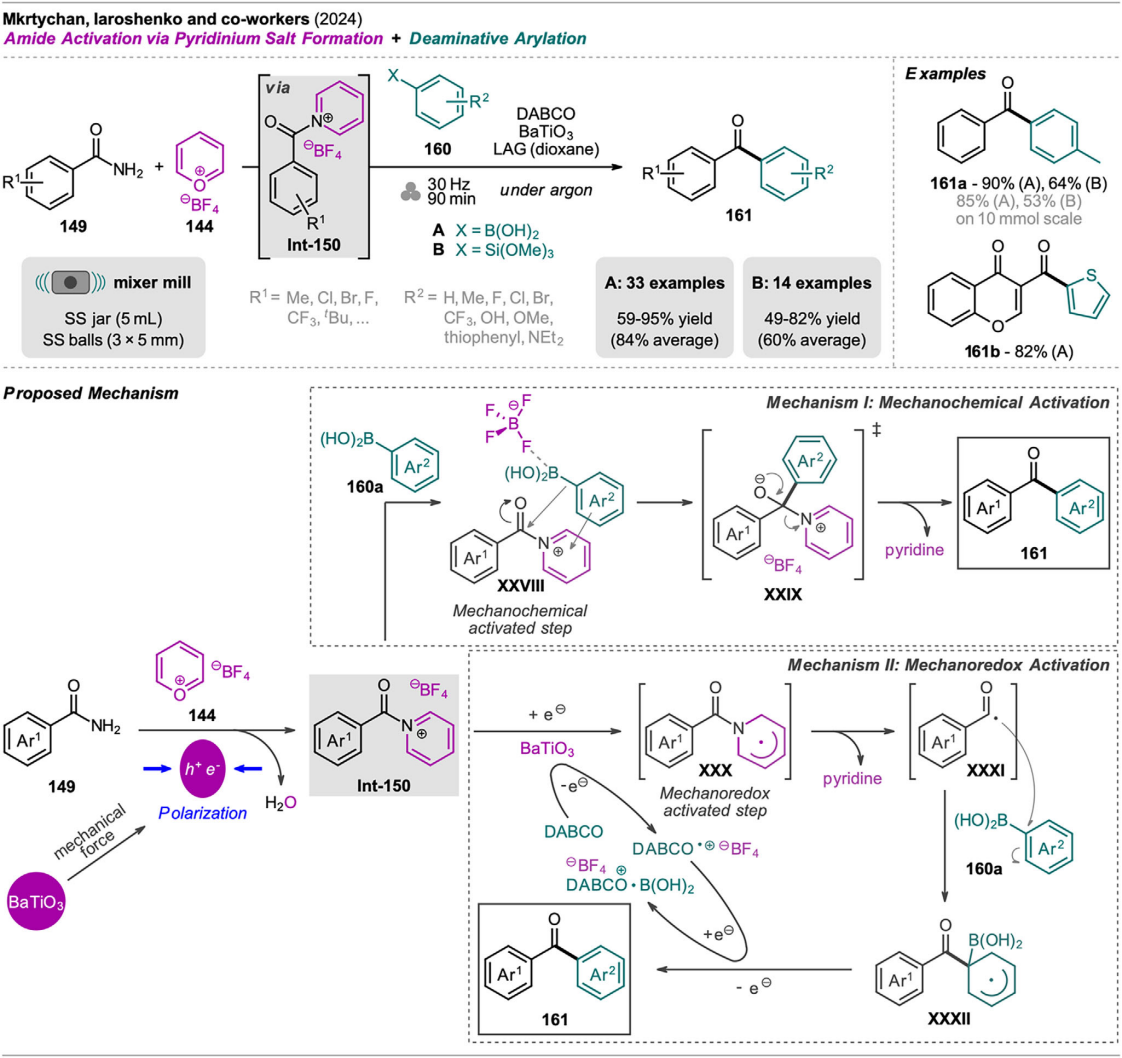
Deaminative arylation of amides for the synthesis of ketones via in situ generated pyridinium salt intermediates.

Mkrtychan, Iaroshenko, and coworkers developed a domino‐type mechanochemical protocol for the deaminative arylation of amides yielding aromatic ketones by activation of the C‐N bond via pyridinium salts (Scheme 33),^[^ ^148^ ^]^ a strategy in close similarity to their other work presented earlier (see Scheme 29).^[^ ^136^, ^137^, ^138^, ^144^ ^]^ Interestingly, the use of piezoelectric barium titanate to enable the decarbonylative functionalization of amides (see Scheme 30) originated from this work, but already here, the exact role of BaTiO_3_ in the transformation could not be unambiguously identified. Optimization studies revealed that amides **149** can be arylated under mechanochemical conditions with boronic acids **160a** or aryl trimethoxysilanes **160b** as aryl sources via activation as pyridiniumsalt **Int‐150**, in the presence of DABCO as a base and piezoelectric BaTiO_3_ under LAG conditions (1,4‐dioxane). Notably, this approach enables the mechanochemically driven C‐C coupling of boronic acids (or trialkoxysilanes) in the absence of a transition metal catalyst.

The authors proposed two different mechanisms for the deaminative arylation process involving pyrylium tetrafluoroborate and barium titanate: (I) via mechanochemical activation and (II) via mechanoredox activation, both displayed in Scheme 33. For both mechanisms it is proposed that BaTiO_3_ is highly polarized by mechanical stress. Following the suggested mechanism I, **Int‐150** gets excited due to the energy input by barium titanate, enabling the nucleophilic addition of the aryl group of **160a** at the carbonyl moiety (intermediate **XXVIII**). Upon elimination of pyridine from transition state **XXIX**, the targeted biaryl ketone **161** is formed. For the alternatively proposed mechanism II, polarized barium titanate enables single electron transfer from DABCO to **Int‐150**, resulting in transition state **XXX** which upon elimination of pyridine forms the acyl radical **XXXI**. This radical then allows direct coupling with the boronic acid **160a** to form radical **XXXII**. From this intermediate, single electron transfer back to the DABCO species takes place to ensure a continuous redox cycle and release the desired product **161**.

The use of piezoelectric BaTiO_3_ as a mechanoredox activator in mechanochemical transformations has been shown in earlier reports.^[^ ^149^, ^150^, ^151^, ^152^, ^153^ ^]^ However, in these transformations direct single electron transfer from or to BaTiO_3_ is proposed and not just its ability to release energy in its polarized state.

For proof of the involvement of the pyridinium salt **Int‐150**, the reaction was also performed in a sequential one‐pot two‐step fashion and from isolated **Int‐150**, both were successful.

As for the other methods, a variety of aryl amides, aryl boronic acids, and aryl silanes with electronically different substituents (e.g., Me, F, CF_3_, NEt_2_) gave the ketone products **161** in good to excellent yields. Comparing the two investigated coupling agents, in general higher yields were observed when boronic acids were used (see, e.g., compound **161a**). It is noteworthy that in addition to aryl amides, ureas were also well‐tolerated as substrates and successfully transformed to the corresponding amides by this method (not shown). Scale‐up experiments (10‐fold, 10 mmol) were successful too, and the ketones were obtained in gram quantities in comparable yields to the smaller scale.

Interestingly, this method was again found to only work in the solid state, as attempts to perform the transformations in solution failed completely, even when performed at elevated temperatures and with prolonged reaction times. This observation is somehow evidence for the mechanistic consideration that BaTiO_3_ is activated by the mechanical stress in the milling approach, helping to overcome the reaction's activation barrier for the deamination step.

## 5 Synthesis of APIs

### 5.1 Rufinamide

Rufinamide is an anti‐epileptic drug with a triazole derivative structure.^[^ ^154^ ^]^ Triazole scaffolds are in general of use in medicinal chemistry and incorporated in various drugs with letrozole, fluconazole, or rufinamide as representatives.^[^ ^155^, ^156^ ^]^ Furthermore, bioactive triazole derivatives play an important role in agriculture, especially often used as the active unit of antifungal agents.^[^ ^157^ ^]^

**Scheme 34:**
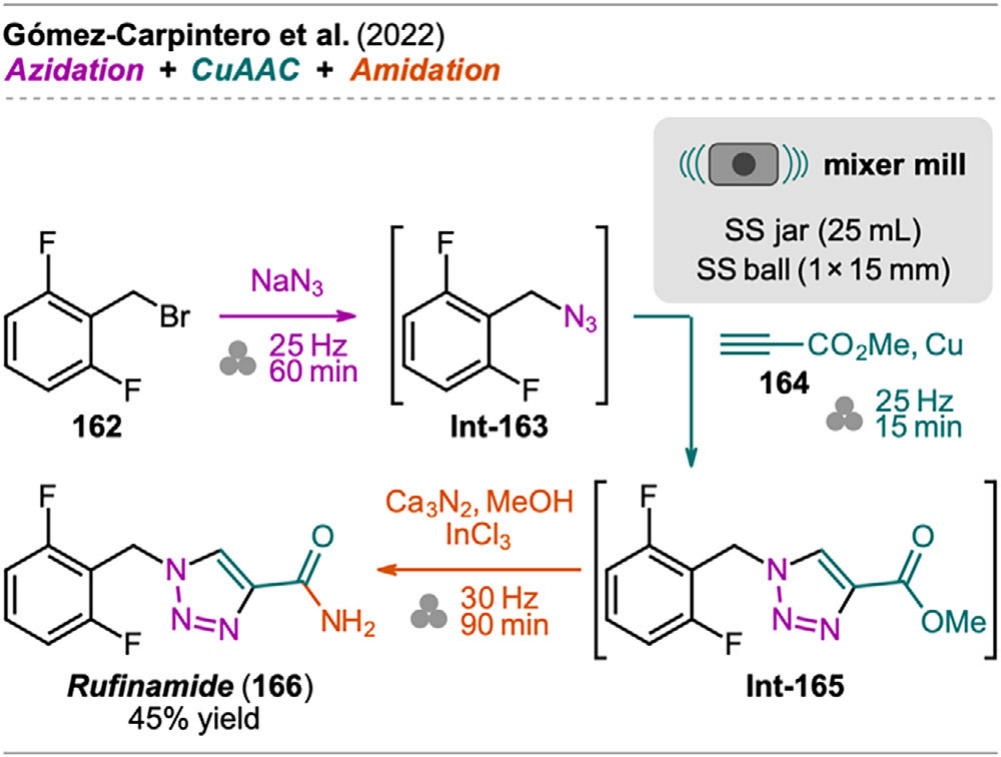
One‐pot mechanochemical synthesis of the anti‐epileptic drug rufinamide via an azidation, CuAAC and amidation sequence.

The mechanochemical synthesis of rufinamide representing a 1,4‐disubstituted 1,2,3‐triazole via a sequential one‐pot three‐step protocol was developed by Gómez‐Carpintero et al. (Scheme 34).^[^ ^158^ ^]^ In the presented approach, the azide formation, CuAAC and amidation were combined sequentially in one‐pot starting from 2,6‐difluorobenzyl bromide (**162**). The azide intermediate **Int‐163** was formed by milling of bromide **162** with sodium azide using a mixer mill. The subsequent CuAAc with methyl propiolate (**164**) using Cu powder as a catalyst gave the 1,2,3‐triazole intermediate **Int‐165** regioselective in a short time of only 15 minutes. Other tested Cu(I) catalysts also resulted in high conversion of **Int‐163**, however leading to the formation of more byproducts compared to Cu powder. **Int‐165** was finally transformed into rufinamide (**166**) by amidation with calciumnitride and methanol (ammonia sources) and indium trichloride as a catalyst. The method did not require the isolation of any intermediate product and rufinamide could be obtained with an overall yield of 45% in less than 3 hours and high purity after recrystallization.

To assess the future applicability of this synthetic pathway in the pharmaceutical industry, the residual content of iron, manganese, copper, and indium was measured. Monitoring trace metal levels is essential in pharmaceutical production to ensure compliance with safety regulations and to protect patient health. All measured metal contents were below the permitted daily exposure, even at the maximum recommended daily dose of 3200 mg of rufinamide.

A similar synthetic pathway for the synthesis of rufinamide has also been described in one‐pot in water in patent WO2010043849A1.^[^ ^159^ ^]^ However, reaction times were long (in total 38 hours), and heating was required, but rufinamide was isolated by filtration in 50% yield. In comparison to this method, green metrics have been calculated resulting in an E‐factor of 18 for the mechanochemical protocol, but without consideration of the isolation and purification. Also in this case, the metrics calculation is not fully comprehensible and the numbers given in the paper do not match with the numbers shown in the supporting information.

### 5.2 Praziquantel

**Scheme 35:**
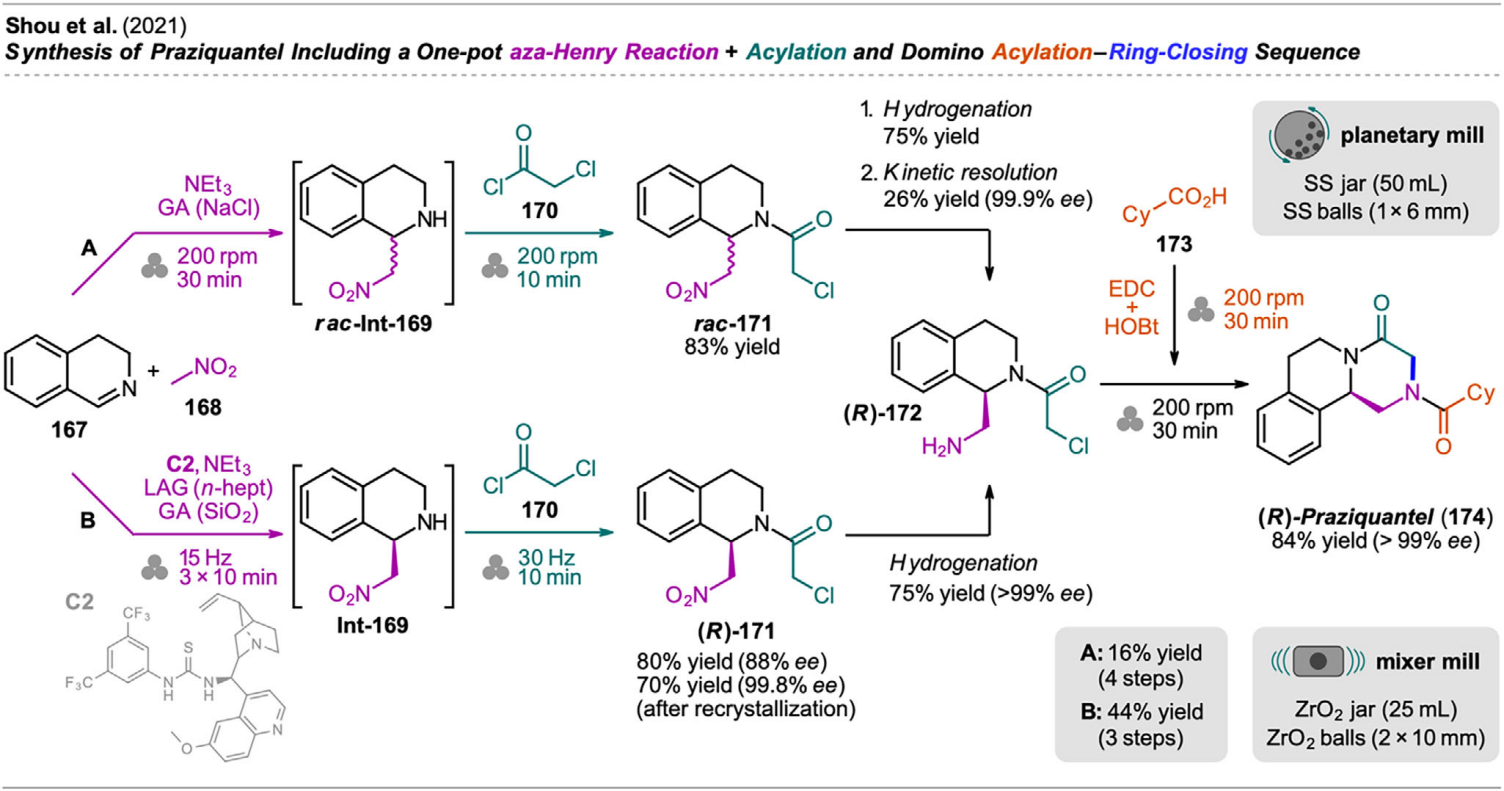
Synthesis of the anti‐parasitic drug (*R*)‐praziquantel including a sequential one‐pot aza‐Henry reaction‐acylation and domino acylation‐ring‐closing sequence.

Aiming for a synthetic route toward praziquantel (PZQ), a first‐line drug for the treatment of the parasitic disease schistosomiasis,^[^ ^160^ ^]^ Shou et al. presented a one‐pot aza‐Henry reaction (racemic and asymmetric version) and acylation as well as a one‐pot acylation and domino cyclization reaction (Scheme 35).^[^ ^161^ ^]^ Their work presented two practical and scalable pathways toward (*R*)‐PZQ (**174**) combining mechanochemical one‐pot transformations with solution‐based steps. Following Path A (Scheme 35A), a mechanochemical aza‐Henry reaction between isoquinoline **167** and nitromethane (**168**) was performed, using triethylamine as a base and NaCl as GA. The racemic intermediate ***rac*‐Int‐169** obtained from this reaction was subsequently acylated with chloroacetyl chloride **170** in one pot to produce ***rac*‐171** in a good yield of 83%. To obtain enantiomeric pure material, the authors performed hydrogenation in solution to reduce the nitro group to an amine, followed by kinetic resolution to isolate **(*R*)‐172**. However, the latter step turned into an elaborate process when racemization of undesired (*S*)‐enantiomer and recovery of the resolution agents were included. To overcome these challenges, the authors designed an asymmetric version of the mechanochemical aza‐Henry reaction to selectively synthesize the desired (*R*)‐isomer **(*R*)‐169**, improving the overall yield and efficiency (Scheme 35B). The thiourea‐based catalyst **C2** was found to successfully induce enantioselectivity allowing the asymmetric synthesis of **(*R*)‐171** via one‐pot aza‐Henry reaction and acylation, as described before. Although the enantioselectivity of this transformation was not excellent, recrystallization allowed the separation from undesired **(*S*)‐171** and **(*R*)‐171** could be isolated with an overall yield of 70% and >99 *ee*. Subsequent solution‐based hydrogenation then delivered amine **(*R*)‐172** with well‐preserved enantiopurity. With this precursor for praziquantel (**174**) in hand, the final step in the synthesis involved a one‐pot domino acylation and ring‐closing reaction. **(*R*)‐172** was coupled with cyclohexanecarboxylic acid (**173**) using 1‐ethyl‐3‐(3‐dimethylaminopropyl)carbodiimide (EDC) and 1*H*‐1,2,3‐benzotriazol‐1‐ol (HOBt) as coupling agents, forming an intermediate that readily cyclized without additional base. This last step then provided (*R*)‐praziquantel (**174**) in 84% yield while maintaining excellent enantiopurity (>99%).

Notably, all steps in both routes were scalable, demonstrating the practicality of these methods for the synthesis of (*R*)‐PZQ. Comparing the two pathways, the asymmetric approach (Path B) offered a concise formal three‐step route with an overall yield of 44%, representing a significant improvement in efficiency and practicality over the racemic pathway, which gave **174** in 16% yield over four steps.

Compared to exclusively solution‐based pathways,^[^ ^162^, ^163^ ^]^ this combination of mechanochemical (one‐pot) reactions and solution‐based steps represents an efficient synthetic pathway toward praziquantel. Furthermore, the developed mechanochemical asymmetric aza‐Henry reaction might be of interest for the synthesis of other chiral building blocks in the future.

### 5.3 Paracetamol

In recent years, a few mechanochemical approaches for the synthesis of the analgesic and antipyretic drug paracetamol have been reported, for example, by Geib et al. via Beckmann rearrangement from 4‐hydroxyacetophenon,^[^ ^164^ ^]^ or by Portada et al. via reduction and acetylation of 4‐nitrophenol.^[^ ^165^ ^]^ However, both approaches are operated in a consecutive manner, requiring isolation of the oxime or 4‐aminophenol intermediate, respectively.

**Scheme 36:**
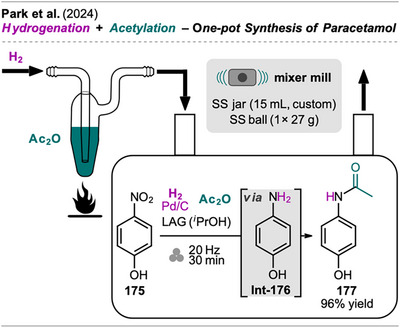
One‐pot synthesis of the analgesic and antipyretic drug paracetamol via one‐pot hydrogenation and acetylation of 4‐nitrophenol.

Park et al. presented an innovative mechanochemical approach for the synthesis of the analgesic drug paracetamol (**177**) from 4‐nitrophenol (**175**) through one‐pot hydrogenation and acetylation (Scheme 36).^[^ ^166^ ^]^ Park et al.’s work focused on achieving high selectivity for paracetamol while minimizing byproduct formation (e.g., by over‐acetylation and condensation) and avoiding the use of expensive reagents or large quantities of additives. At first, catalytic transfer hydrogenation was explored for the reduction of **175** to the corresponding amine **Int‐176**. This approach was based on Portada et al.’s protocol,^[^ ^165^ ^]^ who reported quantitative yield for **Int‐176**, however, these results could not be successfully reproduced by Park et al., resulting in unsatisfactory selectivity for the amine (83%). Efforts to improve the selectivity of the hydrogenation were taken and selectivity was increased under LAG conditions, but combination with acetylation in one‐pot again resulted in selectivity problems for paracetamol (**177**). Drawing their attention to one‐pot elemental hydrogenation and acetylation, the selectivity could be improved, and the best yield and selectivity for the desired product **177** was obtained when acetic anhydride was “fed batch” to the jar, which contained nitrophenol **175**, Pd on charcoal, and isopropanol for LAG. A unique reactor setup was employed: hydrogen gas was flowed through a heated bubbler containing acetic anhydride, and the stream of hydrogen and gaseous anhydride passed through the jar which was shaken at 20 Hz frequency. The flow rate of the hydrogen gas was set to match the conversion rate of aminophenol intermediate **Int‐176**, ensuring precise control over the amount of gaseous Ac₂O introduced to prevent over‐acetylation. Under these optimized conditions, paracetamol was obtained with 96% yield and 99% selectivity. Assessing sustainability, recyclability of the Pd catalyst was investigated as well as the effect of ball milling on its activity. Remarkably, four consecutive reaction cycles could be performed by introducing additional **175** without exposure to air with no loss in catalytic activity.

For comparison, the authors also performed the reaction in a solution‐based one‐pot setup using a Parr apparatus, and calculated the RME and PMI for the investigated methods. While the solution‐based process showed a slightly better RME (33% for SC vs. 28% for MC), owed to reduced hydrogen gas amount, the mechanochemical protocol exhibited a significantly better PMI (6.4 for MC vs. 20–80 for SC) due to the substantial reduction of solvent usage.

### 5.4 Rasagiline

**Scheme 37:**
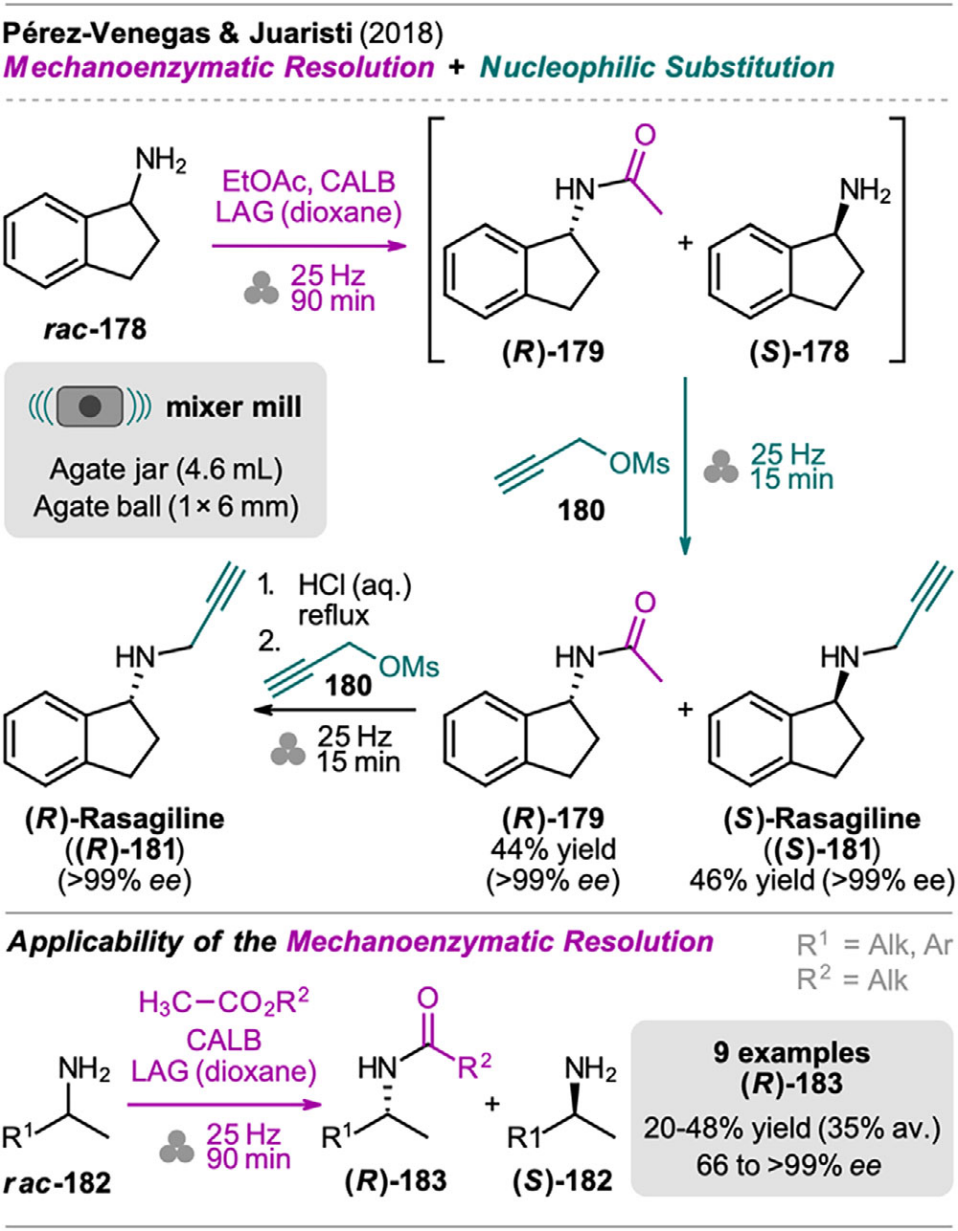
Synthesis of (*S*)‐rasagiline via sequential mechanoenzymatic resolution and nucleophilic substitution including the straightforward synthesis of (*R*)‐rasagiline.

Rasagiline in its *R*‐enantiomer form is a drug that acts as an irreversible inhibitor of mitochondrial monoamine oxidase B and is widely used in the management of Parkinson's disease.^[^ ^167^ ^]^ Pérez‐Venegas and Juaristi reported a one‐pot, mechanochemical synthesis of (*S*)‐rasagiline (**(*S*)‐181**) which also facilitated the straightforward synthesis of (*R*)‐rasagiline (**(*R*)‐181**) through combination of mechanochemical and solution‐based steps (Scheme 37, top).^[^ ^168^ ^]^ The approach started from racemic indanylamine ***rac*‐178** and mechanoenzymatic resolution was performed as the first step. Prior investigations on this transformation, which included a substrate scope of various alkyl and aryl substituted primary amines ***rac*‐182** (Scheme 37, bottom), resulted in the presented reaction conditions: Racemic indanylamine ***rac*‐178** was milled with immobilized *Candida anatarctica* Lipase B (CALB, immobilized on acrylic resin), ethyl acetate as an acetyl source, and dioxane as a LAG agent, giving a mixture of amide **(*R*)‐179** and unreacted **(*S*)‐178**. Upon complete conversion, propargyl mesitylene **180** was added to the jar, and further milling resulted in alkylation of **(*S*)‐178**. The obtained mixture of **(*R*)‐179** and **(*S*)‐181** could be separated via CC, affording both compounds in high yield and excellent enantiopurity (>99% *ee*). The pharmacologically more relevant **(*R*)‐181** was obtained via consecutive deprotection in solution and a final mechanochemical alkylation step with **180**.

Addressing the sustainability of the process, the authors evaluated the recyclability of the used catalyst with phenylethylamine as a model substrate. Although the recovered catalyst retained activity and continued to deliver the product in high enantiopurity (>99% *ee* for **(*R*)‐183a**, R^1^ = Ph, R^2^ = Me), a reduction in yield was observed, indicating diminished catalytic efficiency upon reuse. Additionally, the scalability of the process was explored with the same substrate. Notably, dioxane was not required as a LAG additive during scale‐up, and **(*R*)‐183a** was obtained without significant decrease in yield. However, the authors did not specify the exact scale of this experiment.

Compared to similar solution‐based methods, such as the protocol reported by Thalén et al.,^[^ ^169^ ^]^ this mechanoenzymatic protocol offers several advantages, including drastically shorter reaction times (MC: 90 minutes, SC: 3 days), a simple set‐up that does not require inert conditions or high temperatures, and a significant reduction of solvent use, highlighting the potential of the mechanoenzymatic strategy for the preparation of biologically active molecules.

### 5.5 Antipsychotic Agent PZ‐1190

**Scheme 38:**
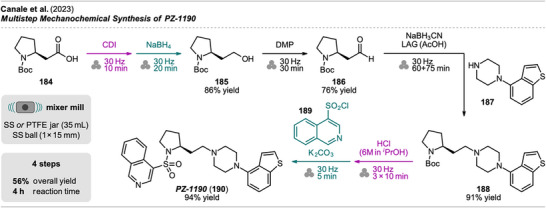
Mechanochemical synthesis of the antipsychotic agent PZ‐1190 including a one‐pot amine deprotection and sulfonylation sequence.

PZ‐1190 is a potential antipsychotic agent that was identified as a multitarget ligand for serotonin and dopamine receptors, showing potential for the treatment of schizophrenia and other central nervous system diseases as, for example, anxiety or depression.^[^ ^170^ ^]^ Canale and coworkers developed a mechanochemical multistep synthesis for PZ‐1190 (**190**), achieving a fast and efficient process through sequential one‐pot transformations and minimal intermediate isolation (Scheme 38).^[^ ^171^ ^]^ In their approach, the authors adapted the synthetic route reported by Zajdel, Popik, and coworkers,^[^ ^170^ ^]^ translating each step into a more sustainable mechanochemical protocol. The synthesis started from *N*‐Boc protected l‐β‐homoproline **184**. Using a two‐stage procedure involving activation of **184** with *N*,*N*'‐carbonyldiimidazole (CDI) followed by reduction with NaBH_4_, the reaction was found to be fast and efficient resulting in complete conversion in 30 minutes. In contrast, the solution‐based approach required lithium aluminum hydride, a strong reducing reagent, and the reaction had to be performed with exclusion of moisture at elevated temperature and took 4 hours. The next two steps were the oxidation of **185** to aldehyde **186** and reductive amination with **187** to obtain **188**. Since these two steps were performed sequentially with isolation of **186**, we do not want to go into detail here, however, a few adaptations compared to the solution‐based approach can be highlighted: For the oxidation, DMP allowed efficient conversion of **185** into **186** within 30 minutes using only 0.5 equivalents of oxidizing agent, compared to 3 equivalents of IBX with 24 hours reaction time in solution. The reaction time for the reductive amination could be shortened to 2 hours (vs. 4 hours), however, still required a toxic reducing agent.

For the final steps ‐ the amine deprotection and subsequent sulfonylation ‐ a sequential one‐pot protocol was developed. After milling of **188** with hydrochloric acid in isopropanol, potassium carbonate, and azinesulfonyl chloride **189** were added, affording product **190** in excellent yield and enantiomeric purity (>99% *ee*). This last transformation was clearly superior to the solution‐based approach in terms of reaction time (MC: 35 minutes vs. SC: 10 hours) and overall yield (MC: 94% vs. SC: 72% over 2 steps). Furthermore, toxic triethylamine was replaced by nontoxic potassium carbonate.

With the mechanochemical approach, PZ‐1190 (**190**) was synthesized in gram‐scale quantities (1.24 g) with just 4 hours of total milling time, achieving an impressive overall yield of 56% over 4 steps, clearly outperforming the five‐step synthesis in solution (32% overall yield, 42 hours total reaction time). Notably, the entire mechanochemical process eliminated the need for chromatography or recrystallization steps, and all intermediates and the final product were obtained through simple extraction, thereby minimizing waste and enhancing the sustainability of the process. This is further evidenced by comparison of the E‐factors: the mechanochemical route achieved an overall E‐factor of 228, in clear contrast to 2578 for the solution‐based process.

### 5.6 Oligonucleotide Synthesis

Within therapeutics, oligonucleotide‐based APIs have made rapid progress and several oligonucleotide therapeutics for the treatment of various diseases have been approved by the US FDA.^[^ ^172^ ^]^ While unmodified oligonucleotides possess poor drug‐like properties, modified oligonucleotides (base, sugar, and backbone modifications; conjugates with proteins) have been found to be valuable therapeutics due to enhanced stability, RNA affinity, and pharmacokinetic properties.^[^ ^173^ ^]^ Regarding the synthesis of oligonucleotides, two prominent strategies have to be named: phosphoramidite and *H*‐phosphonate chemistry.^[^ ^174^, ^175^ ^]^ However, the state‐of‐the‐art production of oligonucleotides via phosphoramidite chemistry remains challenging in terms of sustainability, with large amounts of waste as a major challenge.^[^ ^176^ ^]^

**Scheme 39:**
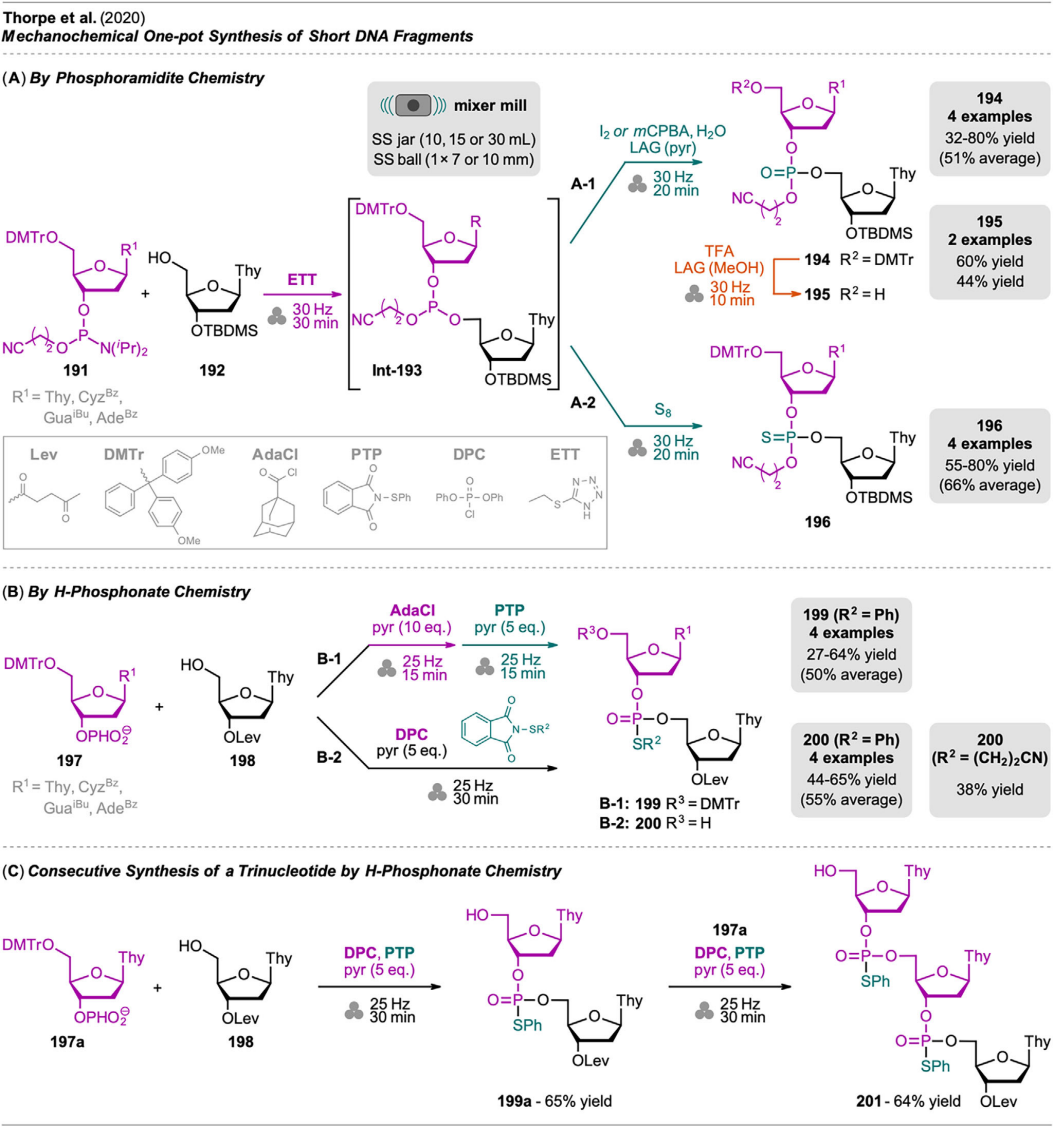
Mechanochemical one‐pot synthesis of dinucleotides (A) by phosphoramidite and (B) *H*‐phosphonate chemistry; (C) Exemplified consecutive synthesis of a trinucleotide by *H*‐phosphonate chemistry.

Contributing to a greener, more sustainable direction for oligonucleotide synthesis, Thorpe et al. developed a mechanochemical protocol for the one‐pot synthesis of short DNA fragments, 3′‐5′‐linked dinucleotides, presenting two approaches: via phosphoramidite and *H*‐phosphonate chemistry (Scheme 39).^[^ ^177^ ^]^ Mimicking the solution‐phase phosphoramidite synthesis cycle, the mechanochemical synthesis was started with the coupling of nucleoside phosphoramidite derivatives **191** with TBDMS‐protected nucleoside **192** 5‐(ethylthio)‐1*H*‐tetrazole (ETT) as coupling agent (Scheme 39A). The resulting dinucleotide **Int‐193** was subsequently oxidized to the more stable phosphate triester species **194** in one‐pot by the addition of solid iodine, water, and pyridine as a LAG agent, and further milling for 20 minutes (Scheme 39A‐1). Under these conditions, dinucleotides **194** were obtained in moderate to good yields, although the isolated yield for **194** with R^1^ = Gua^iBu^ was very low due to premature loss of the DMTr protecting group, leading to difficulties in the isolation. However, this issue was resolved by using *meta*‐chloroperoxybenzoic acid (*m*CPBA) as an oxidant instead of iodine, allowing the isolation of the protected dinucleotide. Aiming for the 5′‐OH dinucleotides **195**, the authors demonstrated that deprotection under acidic conditions (TFA) could also be performed sequentially in one‐pot in addition to the coupling and oxidation steps. This approach yielded dinucleotides **195** (R^1^ = Thy or Gua^iBu^) in 60% or 44%, respectively, over three steps.

In an additional variation of the protocol, **Int‐193** was oxidized by the one‐pot treatment with sulfur powder to obtain the corresponding phosphorothioate dinucleotides **196** in good yields (Scheme 39A‐2), which are broadly applied in the area of oligonucleotide therapeutics.^[^ ^172^, ^178^, ^179^, ^180^ ^]^

The authors furthermore turned their attention to *H*‐phosphonate coupling (Scheme 39B). Using adamantoyl chloride (AdaCl) as the activator for the condensation, milling of equimolar amounts of phosphonate **197** and nucleoside **198** in the presence of pyridine gave the intermediate *H*‐phosphonate diester, which could not be isolated efficiently due to its low stability under basic conditions. However, by sequential addition of a sulfur transfer reagent, *N*‐(phenylthiol)phthalimide (PTP), along with additional pyridine, the stable, protected dinucleotides **199** were isolated after further milling (Scheme 39B‐1). Investigations on various activators revealed that replacement of AdaCl with diphenyl phosphoryl chloride (DPC) allowed the coupling and sulfurization step in a single operation step, without the need for sequential addition of reagents (Scheme 39B‐2). Under these conditions, fine‐tuning of the pyridine amount enabled simultaneous cleavage of the 5′‐DMTr protecting group, resulting in dinucleotides **200**.

Noteworthy, the consecutive synthesis of a trinucleotide **201** was also demonstrated, exemplified with dinucleotide **200a**, which was reacted with nucleoside **197a** under the conditions of Path B‐2 (Scheme 39C).

This study highlights the feasibility of using mechanochemical one‐pot multistep reactions to efficiently assemble oligonucleotide structures and suggests that these dimers and trimers could potentially be applied as precursors to traditional solid‐phase oligonucleotide synthesis, streamlining the process by reducing the number of synthesis cycles.

## 6 Summary and Outlook

Mechanochemical one‐pot multistep reactions have emerged as a powerful and sustainable approach in modern organic synthesis. By the combination of multiple steps in one reaction vessel, this strategy offers distinct advantages over consecutive reaction operations, including reduced operation times, solvent‐free or solvent‐minimized conditions, less waste‐intensive purification steps, and often improved overall efficiency. The reviewed literature highlights the broad applicability of this technique across diverse transformations, ranging from heterocycle formation and functional group interconversions to the mechanochemical one‐pot synthesis of APIs. The growing interest of research in this field demonstrates its capacity to facilitate more environmentally benign and resource‐efficient chemical processes.

Despite these advantages, several challenges remain. The combination of multiple steps in one pot can be challenging and often not simply realized by linear combination of optimized reactions. Logical changes in the reaction conditions are necessary to achieve compatibility, ensure the desired reactivity, and circumvent the formation of side products which also requires a deep understanding of mechanistic pathways in the solid state. It has to be mentioned that mechanistic investigations in the solid state are of greater difficulty as compared to the solution phase. In the latter, many analytical methods are established, and even theoretical methods, such as DFT‐calculations are focused on solution‐phase organic synthesis. Hence, further development of analytical methods or adjustment of existing methods for solid‐state organic synthesis is highly desired.

Additionally, mechanochemical processes require fine‐tuning of the milling conditions, including frequency, ball‐to‐powder ratio,^[^ ^21^ ^]^ and the use of solid and liquid grinding additives,^[^ ^24^, ^25^ ^]^ to achieve optimal yields and selectivity. Therefore, numerous factors must be considered alongside the choice of suitable reactants and reagents. Furthermore, the scale‐up of mechanochemical multistep reactions also remains an area requiring further attention, as batch‐to‐batch reproducibility and process control are crucial for industrial applications.^[^ ^181^ ^]^

Future developments in this field are expected to focus on expanding the reaction scope, particularly in asymmetric synthesis^[^ ^182^, ^183^, ^184^, ^185^ ^]^ and late‐stage functionalization of APIs,^[^ ^108^, ^186^, ^187^, ^188^ ^]^ especially valuable for medicinal chemistry, where mechanochemistry has shown promising success. Additionally, the integration of mechanochemical techniques with automation and continuous‐flow processing provides new opportunities for large‐scale applications.^[^ ^189^, ^190^, ^191^ ^]^ In particular, extrusion‐based mechanochemical processes hold great opportunity for the scale up of solvent‐free or ‐minimized one‐pot multistep synthesis. Advancing mechanochemical synthesis in general, significant potential also lies in the combination of milling with other energy sources, such as light irradiation, sound agitation, or electrical impulses, to enable reactions that are not feasible by conventional mechanochemical processing.^[^ ^192^ ^]^

Overall, mechanochemical one‐pot multistep reactions offer great potential to contribute to greener, more environmentally friendly, and efficient organic synthesis, with significant implications for green chemistry, pharmaceutical development, and material science. As the field continues to evolve, further advances in methodology, mechanistic understanding, and practical implementation will likely establish one‐pot multistep mechanochemistry as a valuable tool for sustainable and efficient chemical synthesis.

## Conflict of Interests

The authors declare no conflict of interest.

## Data Availability

Data sharing not applicable ‐ no new data generated
